# Genomic Drivers of Pyrethroid Resistance Escalation in the Malaria Vector *Anopheles funestus* Across Africa

**DOI:** 10.1093/molbev/msaf251

**Published:** 2025-10-24

**Authors:** Mahamat Gadji, Hervé Raoul Tazokong, Mersimine F M Kouamo, Magellan Tchouakui, Murielle Wondji, Leon M J Mugenzi, Helen Irving, Jack Hearn, Sulaiman S Ibrahim, Charles S Wondji

**Affiliations:** Department of Vector Genomics, Centre for Research in Infectious Diseases (CRID), P.O. BOX 13591, Yaoundé, Cameroon; Department of Microbiology, University of Yaoundé 1, P.O. BOX 812, Yaoundé, Cameroon; Department of Vector Genomics, Centre for Research in Infectious Diseases (CRID), P.O. BOX 13591, Yaoundé, Cameroon; Department of Microbiology, University of Yaoundé 1, P.O. BOX 812, Yaoundé, Cameroon; Department of Vector Genomics, Centre for Research in Infectious Diseases (CRID), P.O. BOX 13591, Yaoundé, Cameroon; Department of Vector Genomics, Centre for Research in Infectious Diseases (CRID), P.O. BOX 13591, Yaoundé, Cameroon; Department of Vector Genomics, Centre for Research in Infectious Diseases (CRID), P.O. BOX 13591, Yaoundé, Cameroon; Vector Biology Department, Liverpool School of Tropical Medicine, Liverpool L3 5QA, UK; Syngenta Crop Protection, Werk Stein, Stein, Switzerland; Vector Biology Department, Liverpool School of Tropical Medicine, Liverpool L3 5QA, UK; Centre for Epidemiology and Planetary Health, Scotland's Rural College (SRUC), RAVIC, Inverness, UK; Department of Vector Genomics, Centre for Research in Infectious Diseases (CRID), P.O. BOX 13591, Yaoundé, Cameroon; Department of Biochemistry, Bayero University, PMB 3011, Kano, Nigeria; Department of Vector Genomics, Centre for Research in Infectious Diseases (CRID), P.O. BOX 13591, Yaoundé, Cameroon; Vector Biology Department, Liverpool School of Tropical Medicine, Liverpool L3 5QA, UK

**Keywords:** *Anopheles funestus*, RNA-Seq, Pool-Seq, Africa, *CPR*, MultiOmics

## Abstract

Aggravation of pyrethroid resistance threatens malaria control; yet, its molecular basis remains elusive. This study used a comprehensive multi-omics framework integrating 7-year gap temporal RNA-Seq, PoolSeq Whole Genome, and functional analyses, to uncover resistance escalation mechanisms in *Anopheles funestus* Africa-wide. Spatiotemporal analyses (2014–2021) reveal massive overexpression of novel genes (V-ATPase, tubulin alpha-1, transposase), alongside canonical resistance genes (P450s, cuticular proteins, chemosensory). Epigenetic regulators (histone H3/4, glycine N-methyltransferase) were greatly overexpressed in highly resistant mosquitoes, suggesting resistance modulation. P450-based signatures of selective sweep were detected with a drastic change in the *rp1* and the P450 *CYP9K1* in Central Africa. Noticeably, genomic variations at the cytochrome P450 reductase (*CPR*) gene were selected including a N70I mutation in Malawi [0% (2009)–80% (2021)] and a 5.9 kb promoter duplication in Ghana. Transgenic expression in *Drosophila* confirmed *CPR*-70I enhances pyrethroid resistance when co-expressed with P450-*CYP6P9a*, uncovering a novel *CPR*-mediated mechanism in intensely resistant mosquitoes. This study highlights novel candidate genes for marker development to track the spread of intensely resistant mosquitoes across Africa.

## Introduction

Malaria control relies heavily on insecticide-based interventions, particularly long-lasting insecticidal nets (LLINs) treated with pyrethroid insecticides, which have contributed to over 68% of the reduction in malaria burden (Bhatt et al. 2015). However, the effectiveness of these tools is increasingly threatened by the rapid rise and spread of insecticide resistance. Worrying cases of high resistance levels to insecticides are increasingly discovered in major malaria vectors such as *Anopheles funestus* leading to extensive loss of efficacy of LLINs including PBO-pyrethroid nets (a P450s-based metabolic resistance inhibitor) (Mugenzi et al. 2019; Weedall et al. 2019). As a result, there are concerns about the potential impact that such intense resistance could have even on the efficacy of novel LLINs, such as Interceptor® G2 (IG2) and Royal Guard®. Intense resistance is the ability of malaria vectors to survive higher doses of insecticides recommended by the WHO, such as 5x and 10x DC (WHO 2016, 2024). Insecticide resistance in malaria vectors is a multifactorial and still not fully understood phenomenon, involving several distinct mechanisms. One such mechanism is cuticular resistance, which reduces insecticide penetration through modifications in the mosquito cuticle's thickness or chemical composition. This has been demonstrated in *Anopheles gambiae*, where changes in cuticular hydrocarbons significantly lower the rate of insecticide absorption (Balabanidou et al. 2016, 2018). Another major mechanism is target-site resistance, often referred to as knockdown resistance (kdr), which results from point mutations in the voltage-gated sodium channel (VGSC) gene. These mutations, such as L1014F/S, interfere with the ability of pyrethroids and DDT to bind to their target, reducing the insecticides’ effectiveness. While these kdr mutations are widespread in *An. gambiae* (Martinez-Torres et al. 1998), they have been broadly absent in *An. funestus*, although recent findings have reported a novel DDT-linked kdr mutation (L976F) in *An. funestus* populations in Tanzania (Odero et al. 2024). The most dominant resistance mechanism in *An. funestus*, however, is metabolic resistance, which is mediated by the overexpression of detoxification enzymes, particularly cytochrome P450 monooxygenases (Wondji et al. 2009; Riveron et al. 2019). Sub-Saharan African countries are facing a problem of intense resistance with malaria vectors increasingly reported as surviving even 10× the diagnostic insecticide concentrations (Tchouakui et al. 2021; Menze et al. 2022; Mugenzi et al. 2022; Nguiffo-Nguete et al. 2023; Tazokong et al. 2024; Gadji et al. 2025). Unless such intense resistance is well managed, recent gains in reducing malaria burden could be lost with dire consequences (Hemingway 2017). Unfortunately, the complex molecular drivers of this resistance escalation remain unknown hindering the design of robust and sustainable resistance management strategies to eradicate and/or eliminate malaria (Hemingway 2017).

Efforts to map the spread of this resistance escalation, especially toward pyrethroids, have revealed that it is now in all African regions albeit at different intensity notably in *An. funestus* and *An. gambiae* (Tchouakui et al. 2021; Menze et al. 2022; Mugenzi et al. 2022; Nguiffo-Nguete et al. 2023; Wangrawa et al. 2024). However, preliminary analyses of over-expression patterns of key resistance genes such as the major P450 genes did not link their over-expression to the increased ability to survive higher doses of pyrethroid insecticides suggesting that the molecular mechanisms driving intense resistance could be more complex (Tchouakui et al. 2021; Menze et al. 2022; Mugenzi et al. 2022). Additional studies employing various omics approaches have documented distinct patterns of gene expression, population structure, selective sweeps, and genetic diversity spanning key genomic loci within both the *An*. *gambiae* complex and the *An*. *funestus* group across Africa suggesting that underlying mechanisms of resistance aggravation could also vary according to geographical regions (Weedall et al. 2020; Ibrahim et al. 2023; Lucas et al. 2024; Nagi et al. 2024). Elucidating mechanisms of super-resistance will provide a deep insight into molecular processes driving the escalation of resistance in mosquitoes notably the role of alternative resistance mechanisms and help design suitable DNA-based diagnostic assays to track and assess the impact of resistance exacerbation in the field. Recent studies of metabolic resistance to insecticides have proven the possibility to develop such molecular markers allowing monitoring the spread of resistance in *An. funestus* across its range (*GSTe2*-L119F, *CYP6P9a*-R, 6.5 kb structural variant (SV), 4.3 kb SV, *CYP9K1*-G454A, *CYP6P4a*-M220I and *CYP6P4b*-D284E) (Riveron et al. 2014; Mugenzi et al. 2019; Mugenzi et al. 2020; Mugenzi et al. 2024; Tatchou-Nebangwa et al. 2024; Djoko Tagne et al. 2025).

Here, we employed a comprehensive omics analysis, combining RNA sequencing (RNA-Seq), Pool sequencing (Pool-Seq), and transgenic expression (in *Drosophila melanogaster* flies) techniques to perform a spatiotemporal genome-wide survey of gene expression and genetic diversity profiles. This allowed to capture major genomic changes and to functionally validate a key candidate gene associated with resistance escalation across four main regions of Sub-Saharan Africa. Our findings reveal that the molecular drivers of resistance escalation are complex, involving significant over-expression of novel genes not previously related with resistance. Other overexpressed genes included detoxification genes, cuticular resistance, and chemosensory adaptations genes, including sensory appendage proteins (SAPs). Additionally, we identified signatures of epigenetic regulation linked to histone genes. Furthermore, we uncovered strong evidence of key genetic variations in the P450s redox partner, the NADPH cytochrome P450 reductase (*CPR-N70I*) and demonstrated that the mutant allele functionally enhances pyrethroid resistance in *An. funestus*.

## Results

### Temporally Differentially Expressed Genes Associated With Resistance Escalation in *Anopheles funestus* Across Africa

We conducted a spatiotemporal transcriptomic analysis of *An. funestus* populations from four African regions (Cameroon, Ghana, Uganda, and Malawi) over a 6- to 7-year period to identify temporal changes in gene expression linked to pyrethroid resistance escalation. The dramatic change in phenotypic resistance during this period directly correlated with a significant drop in pyrethroid susceptibility, making this a particularly interesting time scale to study.

### Genes Commonly and Temporally Overexpressed Across Africa

**Fig. 1.**
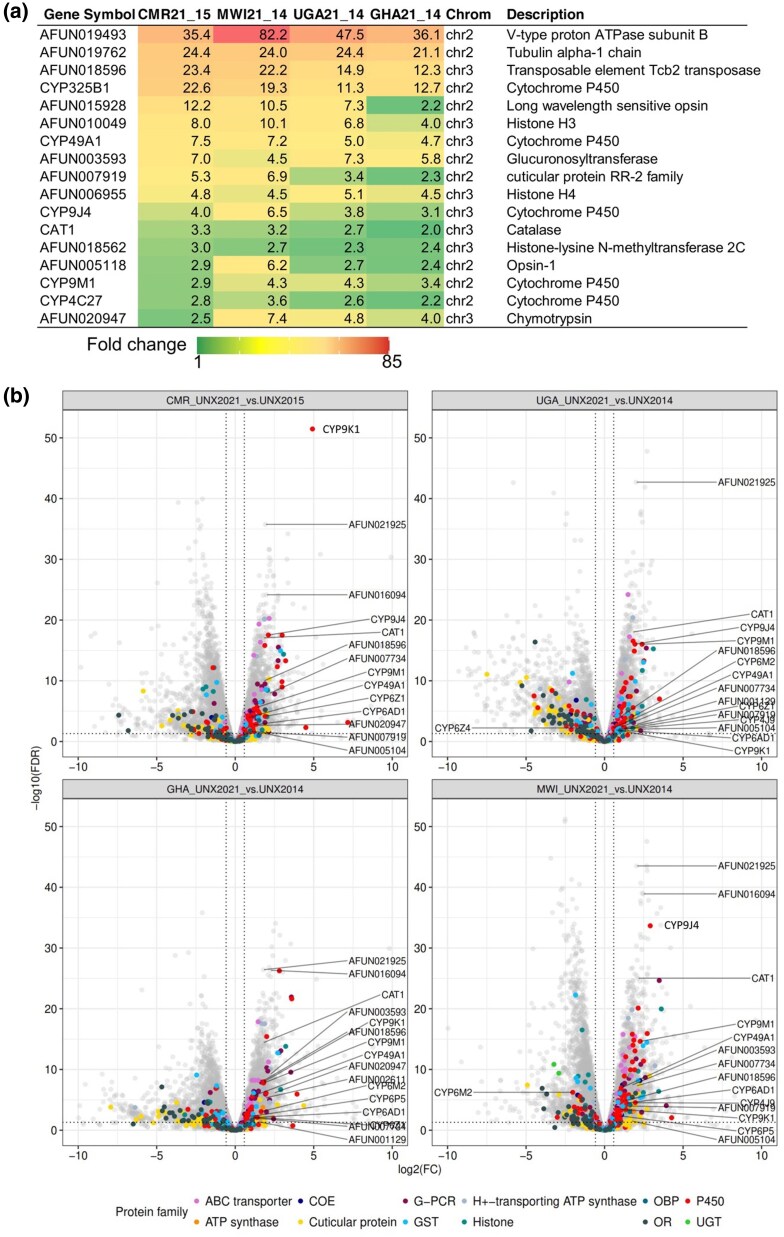
Temporarily and contemporary overexpressed genes in *An. funestus* in the four countries. (a) Heatmap of top genes commonly overexpressed (FC ≥1.5 and FDR <0.05) across the four countries when comparing 2021 against 2014 populations from each country; numbers on the heatmap represent fold change (FC) values. The variation in fold change across countries is depicted using a green-yellow-red color scale, where green indicates low FC (≤4.5), yellow represents moderate FC (5–25), and red denotes high FC (>26). (b) Volcano plots showcasing genes differentially expressed temporarily when comparing 2021 against 2014 populations from each country. The genes highlighted in color belong to known protein families and some putative genes known to be involved in insecticide resistance. Other gene families are depicted in gray. Only top genes belonging to category depicted in protein family caption are annotated. Genes above the horizontal dotted line passed our threshold for significance (FDR <0.05). Genes on the right of the dotted vertical line (log2FC ≥0.58) were up-regulated in the 2021 than in the 2014, whereas genes on the left of the vertical dotted line (log2FC ≤0.58) were down-regulated in 2021 than in 2014. UNX: Unexposed, ABC: ATP-binding cassette; COE: Carboxylesterases, GST: Glutathione S-Transferases, UGT: UDP-glucuronosyltransferases; P450: Cytochrome P450s; OBP: Odorant-binding protein, OR: Odorant receptor, G-PCR: G-Protein Coupled Receptors. CMR, MWI, UGA and GHA represent Cameroon, Malawi, Uganda and Ghana, respectively.

Between 2014 and 2021, a set of 17 genes showed a marked increase in expression (FC ≥ 2 and FDR < 0.05) across the four countries, with fold changes ranging from 2.0 to 82.2 (Fig. 1a). The gene with the highest fold change is V-type proton ATPase subunit B (*AFUN019493*), with fold changes from 35.4 in Cameroon to 82.2 in Malawi, and high read counts (Fig. 1a). Another gene, Tubulin alpha-1 chain (*AFUN019762*), exhibited fold changes of 21.1 in Ghana and 24.4 in Cameroon and Uganda.

A set of the cytochrome P450 genes known as phase I xenobiotic metabolisers was also temporally over-expressed including *AFUN019268* (*CYP325B1*), with fold-change values ranging from 11.3 in Uganda to 22.6 in Cameroon, *CYP49A1* (FC: 4.7 to 7.5), *CYP9J4* (FC 3.1 to 6.5), *CYP9M1* (FC: 2.9 to 4.3), *CYP4C27* (FC: 2.2 to 3.6). However, none of the major P450s previously linked with resistance were included because their expression is specific to one, two or three countries and not continent-wide (Fig. 1a). Interestingly, a signature of epigenetic contribution in the resistance escalation was detected with the increased expression of three histones related genes: histone H3 (*AFUN010049*; FC 4.0 to 10.1), histone H4 (*AFUN006955*; FC: 4.5 to 5.1), and histone lysine N-methyltransferase (*AFUN018562*; FC: 2.3 to 3).

Other genes commonly over-expressed represent key resistance contributors including a cuticular protein RR-2 family, *AFUN007919* (FC: 2.3 to 6.9) indicating a role of cuticular resistance; one catalase, *AFUN005054* (FC: 2.0 to 3.3) and one chymotrypsin, *AFUN020947* (FC: 2.5 to 7.4) representing a role of oxidative stress and digestive genes (Fig. 1a); one glucuronosyltransferase (UGT), *AFUN003593* (FC: 4.5 to 7.3) suggesting contribution from other detoxification gene families and two opsin genes *AFUN015928* (FC: 2.2 to 12.2) and *AFUN005118* (FC: 2.4 to 6.2) highlighting the association of G-protein coupled receptors (GPCRs) known to modulate the expression of cytochrome P450s conferring insecticide resistance (Fu et al. 2024) (Fig. 1). A notable finding from this study is the identification of a transposable element, *AFUN018596* (Tcb transposase) with fold-changes (FC) ranging from 12.3 in Ghana to 23.4 in Cameroon. As transposases are known to catalyze movement of transposons to another part of the genome, Tcb over-expression suggests a role of transposable elements into the resistance escalation (Chung et al. 2007; Rostant et al. 2012; Mugenzi et al. 2024).

Analysis of the sets of genes temporally overexpressed in two or three countries included several detoxification genes among which cytochrome P450s were predominant (table S1, Supplementary material). The P450 *CYP9K1* gene on X Chromosome exhibited the highest fold-change with 25-time increased expression in Cameroon in 2021 and 4.9-time in Ghana. It is to be noted that *CYP9K1* was already overexpressed in Uganda in 2014 and remains high in 2021. Another P450 with major increased expression is *CYP6P4b* with FC of 12.9 in Cameroon and 4.6 in Uganda. This gene had higher expression in Ghana in 2014 and remained so in 2021. Three P450 genes, *CYP6M2*, *CYP4J9,* and *CYP6N1*, are equally significantly over-expressed in 2021 in three countries with highest level in Cameroon (*CYP4J9* (FC: 6.5); *CYP6M2* [FC: 6.4]). *CYP6M2* is a common detoxification gene in *An. gambiae* but has so far not been significantly associated with resistance in *An. funestus*.

Several cuticular proteins are more overexpressed in 2021 compared to 2014–2015 mosquitoes suggesting a significant contribution of the reduced penetration resistance mechanism in the aggravation of resistance to bed net insecticides. These genes belong to many families including cuticular protein RR, CPLC, TWDL (table S1, Supplementary material). Other detoxification genes associated with resistance escalation although with moderate fold change include carboxylesterases (*AFUN000775, AFUN016265, AFUN016050, AFUN007734*), ABC transporters (*AFUN016633, AFUN020240, AFUN002834, AFUN015978*), UGTs (*AFUN019845, AFUN004354 and AFUN016158*). Interestingly, we observed increased expression of other non-detoxifying genes, including a chemosensory protein (CSP3) and glycine N-methyltransferase (GNMT: *AFUN005104*) in populations from Cameroon and Malawi (table S1, Supplementary material).

### Genes Overexpressed Temporally in Each Country Relative to FANG 2014 and FANG 2023 Colony

**Fig. 2.**
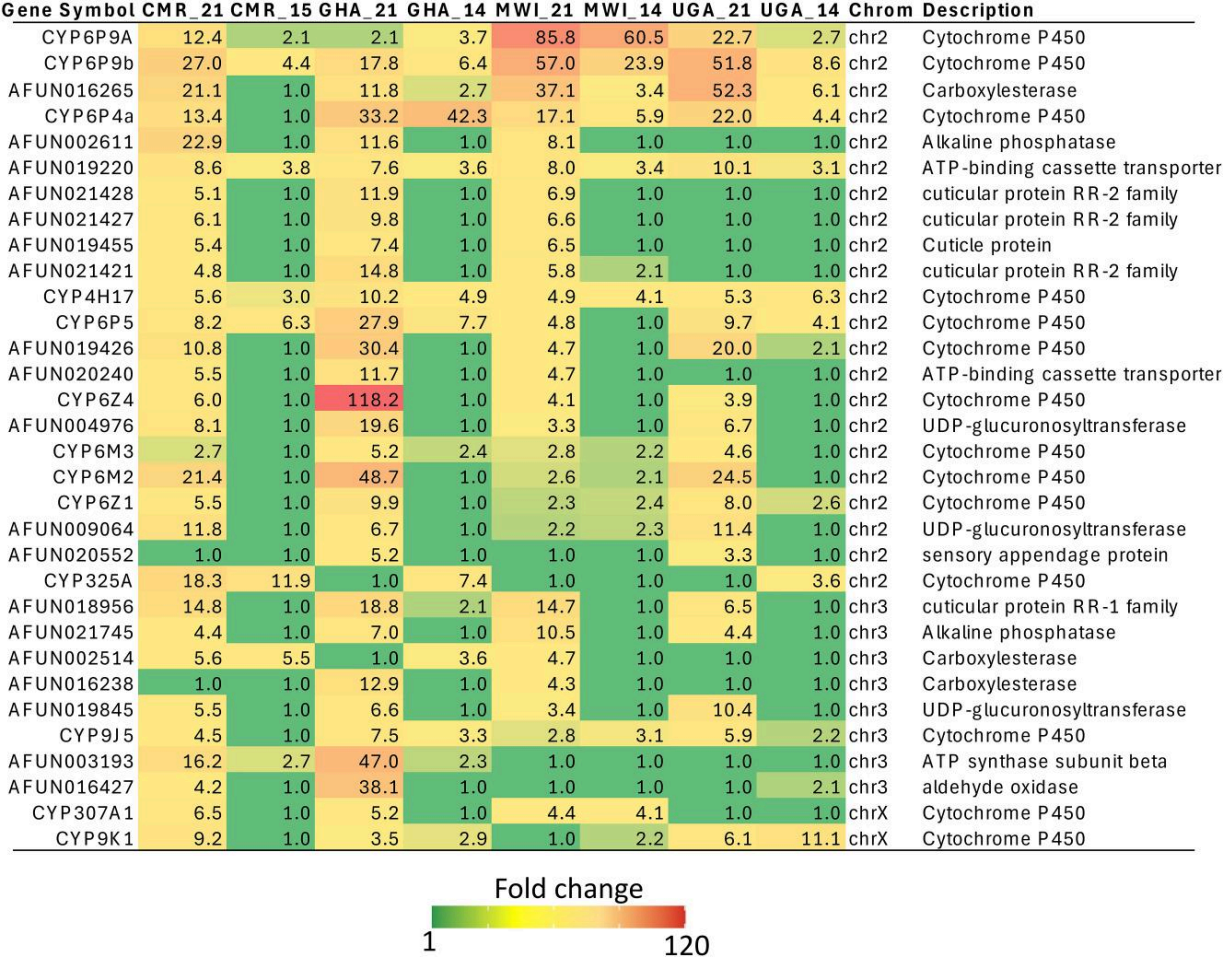
Heatmap showing trends of top genes with significant increase expression (FC ≥2 and FDR <0.05) when considering country_2021 vs FANG_2023 as compared to country_2014 vs FANG_2014; Numbers on the heatmaps represent fold change (FC) values. The variation in fold change across countries is depicted using a green-yellow-red color scale, where green indicates low FC (<3), yellow represents moderate FC (3–30), and red denotes high FC (>31). CMR, MWI, UGA and GHA represent Cameroon, Malawi, Uganda and Ghana, respectively while 21 indicates pairwise comparison between Unexposed 2021 vs FANG 2023 and 14 for Unexposed 2014 vs FANG 2014.

To further confirm the transcriptional change observed between 2021 and 2014 populations in temporal comparison from each location, we performed a second analysis by contrasting 2014 populations to FANG 2014 and 2021 populations to FANG 2023 from each location. This was done to account for batch effect and to remove any bias/noise induced by the sequencing technology or read depth. The principal component analysis supported this approach with four distinct groupings observed: while the different batch of FANG was separated (introduced by sequencing bias) but remained closer due to similar genetic background, the 2014 population was clearly distant from 2021 population as explained by difference in resistance phenotype (Fig. S1). All the detoxification and resistance-related genes with FC ≥ 2 and FDR < 0.05 indicative of increased expression and common in at least two countries were selected (Fig. 2).

#### Transcription Evolution in Cameroon

A greater shift in expression was observed in Cameroon with genes significantly over-expressed and at higher fold changes (Fig. 2). The top over-expressed gene is now the P450 *CYP6P9b* with FC of 27 in contrast to only 4.4 in 2015 (Fig. 2, Fig. S4), likely associated with the selection and fixation of the 4.3 kb enhancer structural variant in this location (Mugenzi et al. 2024). The second differentially expressed detoxification gene was an alkaline phosphatase (*AFUN002611*; FC: 22.9). A remarkable change is the 21.4-fold over-expression of the P450 *CYP6M2* P450 in 2021 (Read count >13 K) whereas it was not even differentially expressed in 2015 (Fig. 2, Figs. S2–S4). This gene is located on the 2L chromosome within the resistance to pyrethroid_2 (rp2) QTL locus as previously described (Wondji et al. 2009). A similar increase in expression is seen for other P450s including *CYP6P4a* (FC: 13.4 vs 1) previously detected in West Africa (Ghana), *CYP6P9a* (FC: 12.4 vs 2.1) and *CYP9K1* (FC: .2 vs 1 with the highest read count of this detox gene at 70 K), *CYP6Z1* (FC: 5 vs 1). Over-expression of *CYP9K1*, located on chromosome X correlated with near fixation of the G454A mutation in this Cameroonian location (Djoko Tagne et al. 2025). The P450 *CYP325A* previously shown to be over-expressed in Cameroon remains so (FC: 18 vs 11 in 2015) although with low read count (221 reads) (Wamba et al. 2021). A carboxylesterase (*AFUN016265*) now exhibits a 21-fold over-expression vs 1 in 2015 although with lower read count compared to P450s (476 reads).

#### Transcription Evolution in Uganda

The top upregulated genes belonging to detoxification enzymes in Uganda were closely the *AFUN016265* carboxylesterase (FC: 52.3 vs 6.1 in 2014; read count <1.2 K) and the P450 *CYP6P9b* (FC: 51.8 vs 8.6 in 2014); read count >23 K) both exhibiting a significant increased expression in 2021 than 2014 (Fig. 2, Figs S2–S4). Similar to Cameroon, significant increased expression was observed for other P450s including on *rp1* linked with the 4.3 kb structural variant (Mugenzi et al. 2024) such as *CYP6P9a* (FC: 22.7 vs 2.7 in 2014), *CYP6P4a* (FC: 22 vs 4.4). The massive increased expression of other P450s from the rp2 locus on 2L chromosome, as seen above in Cameroon, was also observed in Uganda including for *CYP6M2* (FC: 24.5 vs 1 in 2014), *CYP6Z1* (FC: 8 vs 2.6), *CYP6M3* (FC: 4.0 vs 1) and *CYP6Z4* (FC: 3.9 vs 1). In contrast to pattern noticed in Cameroon above, no increased in expression was observed between 2021 and 2014 for the P450 *CYP9K1* potentially due to the fixation of the G454A allele. A significant increased expression of the sensory appendage protein 2 gene (*SAP2*) (*AFUN020552*; FC: 3.9 vs 1 in 2014) was also reported suggesting that this gene family contributes to resistance escalation in Uganda. Increased expression was also observed for other gene families including several cuticular proteins, UDP-glucuronosyltransferases, aquaporin, aldehyde oxidase revealing a diversity of mechanisms driving the aggravation of resistance (table S1).

#### Transcription Evolution in Malawi

The top detoxification genes in Malawi in 2021 were still the duplicated P450 *CYP6P9a/b* but with an increased expression from FC of 60 to 85 for *CYP6P9a* and a doubling of the value for *CYP6P9b* from FC of 23.9 to 57 between 2014 and 2021 (Fig. 2, Figs. S2–S4). A drastic change is noticeable for a carboxylesterase *AFUN016265*, with 37.1-fold expression in 2021 vs 3.4-fold in 2014 (although with lower read counts than P450s: 706 reads). Another carboxylesterase *AFUN002514* previously reported in Cameroon in 2014 is now also over-expressed in Malawi (FC: 4.1 vs 1 in 2014). The P450 *CYP6P4a* has also significantly increased its expression with FC of 17.1 in 2021 vs 5.9 in 2014 as well as *CYP6P5* (FC: 4.8 vs 1), all located on the same rp1 QTL region on 2R chromosome. But contrary to Cameroon, no change was observed for other major P450s such as *CYP9K1* (FC: 2), *CYP6M2*, *CYP6Z1* suggesting that P450 drivers in Malawi are mostly monofactorial on the rp1 locus in line with the 87% genetic variance of pyrethroid resistance explained by rp1 in southern African *An. funestus* (Wondji et al. 2009). Other differentially expressed genes include UDP-glucuronosyltransferases, ABC transporter (*AFUN019220*), several cuticular proteins (table S1).

#### Transcription Evolution in Ghana

*CYP6P4a* remains among the top differentially expressed genes in Ghana (FC: 33.2) although with a slightly reduced expression (FC: 42.1 in 2014) (Fig. 2, Figs. S2–S4). As seen in East and Central Africa, the P450 *CYP6M2* is now massively over-expressed with a FC of 48.1 whereas it was not differentially expressed in 2014 (Fig. 2). Moreover, *CYP6M2* exhibited a high read count (>16,000 reads) suggesting its strong correlation with resistance escalation in Ghana. Other P450 genes from the rp2 locus are similarly over-expressed as seen in 2021 in Central and East Africa including *CYP6Z1*, *CYP6M3*, and *CYP6Z4*. Other gene families associated with escalation in other regions are also detected (Table S1, Supplementary material). These includes *SAP2* (FC: 5.2), epigenetic genes (Histone H3), V-ATPase proton (*AFUN019493*), long wavelength sensitive opsin *AFUN015928* (FC: 32; read count >30 K) (Table S1).

Overall, key P450s, which are well-studied drivers of resistance, were mostly already over-expressed in 2014 and are still (or even stronger) over-expressed in 2021 from each African region indicative of their major contribution to resistance (Fig. S4). But while the duplicated *CYP6P9a*/*b* remain dominant in Malawi, new P450s have been significantly selected in other 3 regions with genes on the *rp2* QTL locus on chromosome 2 exhibiting the most drastic shift such as for *CYP6M2*, which is now among the top over-expressed detoxification genes in West, East, and Central Africa from no significant over-expression in 2014 (Fig. S4). Similarly, the *CYP6P4a* previously over-expressed in 2014, mainly in West Africa (Ghana) has now significantly increased in expression in the other 3 regions although this is likely driven by different haplotypes as it was shown that Ghana haplotypes remain specific to this region (Tatchou-Nebangwa et al. 2024).

### Identification of Genes Linked With Intensification of Permethrin Resistance (Dose Response) in Malawi

**Fig. 3.**
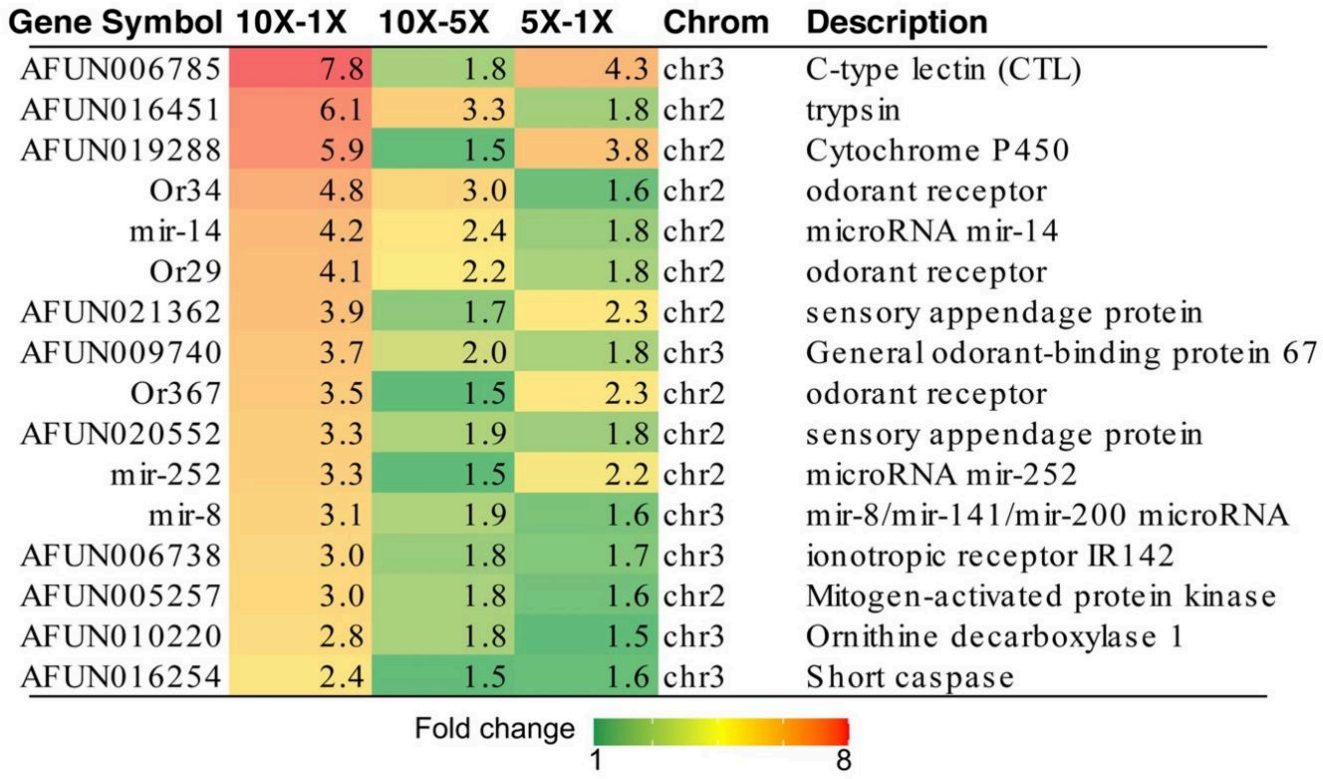
Heatmap of genes commonly overexpressed (FC ≥1.5 and FDR <0.05) in Malawian mosquitoes in the dose response assay (1X, 5X and 10X Diagnostic concentration of permethrin). Contrasts were performed between survivors at 5X vs 1X, 10X vs 1X, and 10X vs 5X pyrethroid doses to identify genes associated with intense pyrethroid resistance in Malawi. Numbers on the heatmaps represent fold change (FC) values. The variation in fold change across different contrast is depicted using a green-yellow-red color scale, where green indicates low FC (<2.1), yellow represents moderate FC (2.2–4.8), and red denotes high FC (>5).

The transcriptional profile of *An. funestus* mosquitoes from Malawi, survivors after exposure to permethrin at diagnostic concentrations of 1X, 5X, and 10X, alongside unexposed controls, was characterized to uncover the molecular mechanisms driving pyrethroid resistance escalation in southern Africa (Fig. 3, Table S2).

### Gene Commonly Upregulated in the Three Permethrin Doses (1X, 5X, and 10X DCs)

To identify genes that are specifically involved in dosing-dependent resistance, priority was given to 10X-1X comparison. This approach allowed us to identify dose-sensitive genes, which are most critical for survival under high selection pressure. This design was intentionally chosen to minimize confounding factors and noise from general stress response and basal resistance coming from using either unexposed or susceptible FANG as comparator. A comparison of 10X, 5X, and 1X survivors to an unexposed mosquito or a susceptible strain FANG would show all genes activated by general stress, not just those related to insecticide resistance. By focusing on 10X versus 1X, we were able to filter out genes that may be constitutively over-expressed in resistant population but not directly tied to the magnitude of resistance. The additional 10X-5X and 5X-1X comparisons were used to further validate this profile by confirming that the identified genes show a proportional increase in expression with the dose of permethrin insecticide (Fig. 3, Table S2).

Special attention was given to the set of genes consistently over-expressed (FC ≥ 1.5 and FDR <0.05) across all three comparisons, highlighting their potential association with the mosquito's ability to survive higher doses of permethrin. This analysis identified 16 genes, including a C-type lectin gene (*AFUN006785*) showing the highest fold-change (FC: 7.8) in the 10X-1X comparison, although read counts were low (<35 reads) (Fig. 3, Table S2). The second most highly over-expressed gene was a trypsin gene (*AFUN016451*). Additional commonly expressed genes belong to gene families associated with olfactory perception, including three odorant receptors (*AFUN007941, AFUN002901, AFUN015900*) and one odorant-binding protein (*AFUN009740*) (Fig. 3). Moreover, two sensory appendage proteins (SAPs)-*AFUN021362* (SAP2) and *AFUN020552* (SAP3) were consistently over-expressed in all three resistance levels, with the highest read counts observed in SAP3: 20,794 reads in the 10X dose, 10,488 in 5X, and 4,190 in 1X. Other commonly over-expressed genes included three microRNAs (mir-14, mir-8, and mir-252), a mitogen-activated protein kinase (MAPK, *AFUN005257*), an ornithine decarboxylase (*AFUN0102220*), and a caspase (*AFUN016254*). The over-expression of these microRNAs highlights the potential role of post-transcriptional regulation in driving resistance escalation. MAPK genes, which are serine/threonine-specific protein kinases, are well-known mediators of cellular responses to various stress stimuli, including insecticide exposure.

Among the genes commonly over-expressed in mosquitoes that survived higher doses of insecticides (5X and 10X) compared to those at 1X, several stress response genes were identified, including two alpha-crystallin B chain genes (*AFUN019875* and *AFUN008656*). Alpha-crystallins have previously been associated with insecticide resistance in mosquitoes (Kwiatkowska et al. 2013). Additionally, several immune response genes, such as C-type lectins, C-type lysozymes, and CLIP-domain serine proteases, were significantly over-expressed (Table S2).

A substantial number of detoxification genes also showed elevated expression levels (Fig. 3). This included three carboxylesterases, with *AFUN002514* demonstrating more than a two-fold increase in expression at 10X and 5X, consistent with patterns observed in Malawi from 2014 to 2021. Several cytochrome P450 genes were highly expressed at 10X and 5X compared to 1X, including *CYP6Z1* located on the rp2 locus, with read counts of 25,800 (10x), 16.600 (5X), and 7.600 (1X). *CYP6M2* also exhibited increased expression with read counts of 8,400 (10X), 4,400 K (5X), and 2,200 (1X), supporting evidence of the temporal rise in the expression of these genes across Africa. Other detoxification genes included members of the glutathione S-transferase family (*GSTe1*, *GSTe8*, and *GSTD3*), sulfotransferases, and UDP-glucuronosyltransferases.

Genes associated with reduced penetration resistance, including various cuticular protein genes, were also over-expressed. Furthermore, 26 genes related to olfaction and taste perception such as odorant receptors and odorant binding proteins (*OBPs*) were detected. Lastly, five microRNA genes were also over-expressed, potentially indicating regulatory roles in resistance mechanisms.

Analysis of genes exclusively over-expressed in mosquitoes that survived 10X insecticide doses compared to those that survived 1X revealed a predominance of detoxification genes, particularly cytochrome P450s, with ten identified. The duplicated P450 gene *CYP6P9b*, which was already one of the most over-expressed genes when comparing unexposed mosquitoes to the FANG strain, showed significant over-expression in the 10X vs 1X comparison. Its read counts were 136,000 in 10X, 94,500 in 5X, and 45,000 in 1X, further supporting the hypothesis that increased metabolic resistance likely drives resistance escalation. Similar trends were observed for other duplicated P450 genes, including *CYP6P4a*, *CYP6P4b*, and *CYP6M3*, all located on the *rp1* and *rp2* regions, suggesting that multiple resistance loci contribute to this escalation (Table S2).

Other detoxification genes, such as *GSTe2* previously implicated in conferring resistance to DDT and pyrethroids in West and Central Africa (Riveron et al. 2014) also showed elevated expression, with 4,600 reads in 10X, 3,300 in 5X, and 1,600 in 1X. Furthermore, three ABC transporters and three solute carrier transporters were identified, suggesting a role for enhanced excretion in resistance escalation.

The over-expression of chemosensory protein 3 (*CSP3*) gene further underscores the involvement of chemosensory pathways, consistent with the consistent over-expression of *SAP2* and *SAP3* observed across all comparisons. Finally, the role of reduced penetration resistance is reinforced by the over-expression of several cuticular protein genes (Table S2). The Gene Ontology enrichment analysis performed confirmed the pattern of gene expression (supplementary text, Fig. S5).

### PoolSeq Whole Genome Spatiotemporal Evolution of *An. funestus* Across Africa

#### Quality Control and Descriptive Statistics of PoolSeq Data

Alignment of PoolSeq data, consisting of 150 bp paired-end reads, yielded between 170 million reads for the Mayuge (Uganda) 2021 population and 184 million reads for the Ghana 2021 population, with an average of 177 million reads (Table S3). After filtering for properly paired reads (retaining only reads that mapped in pairs), sequence quality (Phred score > 10), and mapping quality (*q* > 10), successful alignment was achieved against the *An. funestus* FUMOZ assembly sourced from VectorBase (www.vectorbase.org). Mapping rates ranged from 58.14% for the Uganda 2021 population to 87% for the Malawi 2021 population, with an overall mean of 77%. Among the mapped reads, >97% were properly paired, while <7% were singletons across all samples (Table S3). Coverage metrics showed that more than 98% of the genome was covered in all populations during the sequencing process (Table S4). The mean sequencing depth ranged from 80.25× in the Cameroon 2021 population to 80.47× in the Malawi 2021 population, with an overall mean depth of 70.45× (Table S4), indicating that, on average, each genomic locus was covered by at least 70 reads.

### Population Structure Analysis Among *An. funestus* Populations Across Africa

**Fig. 4.**
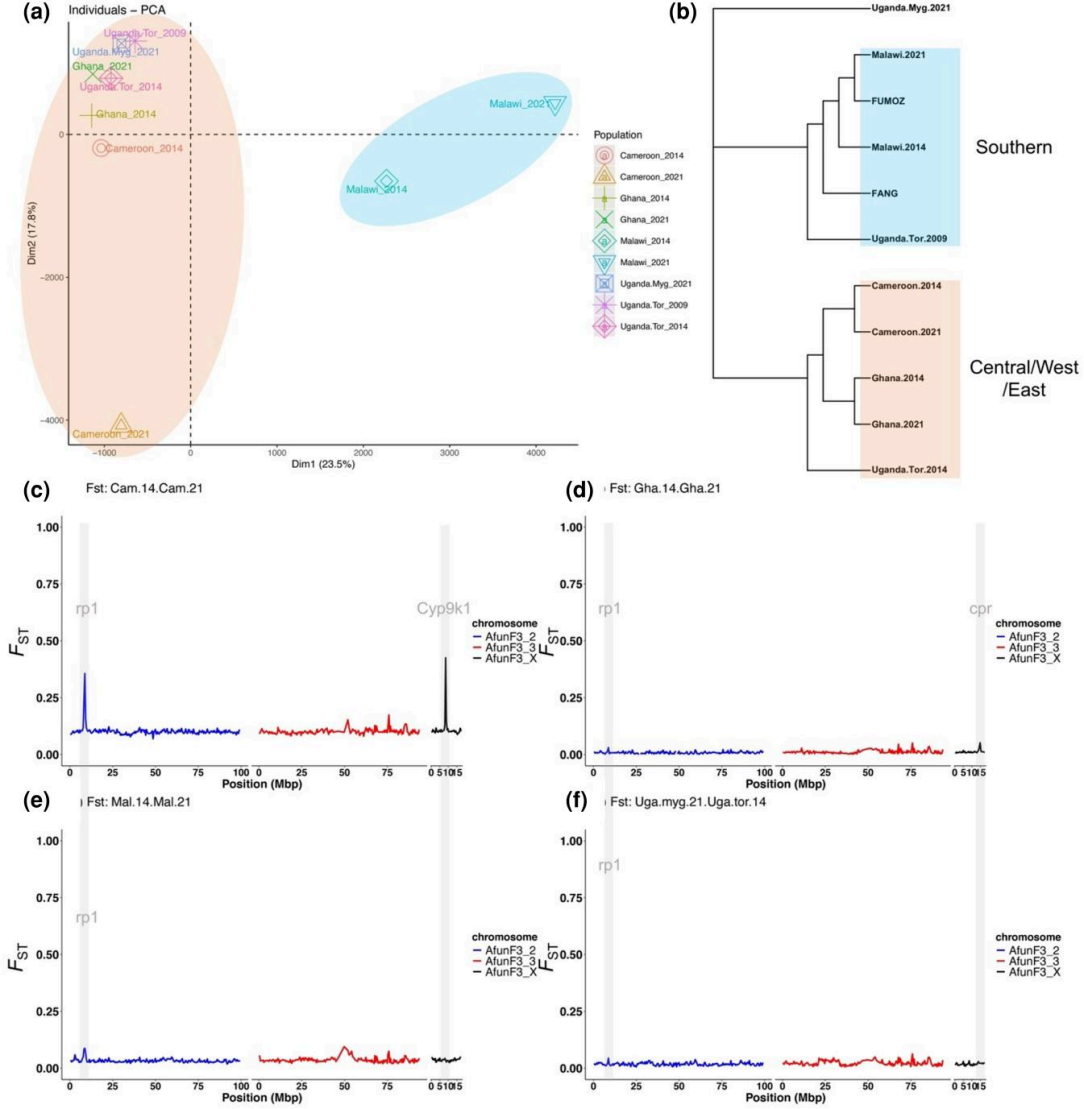
Principal component analysis (PCA); (a) neighbor joining (NJ) tree (b) of *An. funestus* (with 2014 samples form Weedall et al article (Weedall et al. 2020) included for temporal comparison), (c, d, e and f) temporal variation in genetic differentiation in *An. funestus* populations across Africa. The *F*_ST_ genetic differentiation analysis of *An. funestus* mosquitoes between 2014 and 2021 from Cameroon (Cam.14, Cam.21), Ghana (Gha.14, Gha.21), and Malawi (Mal.14, Mal.21). *F*_ST_ values were summarised in non-overlapping windows of 50,000 SNPs, considering only bi-allelic variants. F_ST_ values range from 0 to 1, with higher values indicating greater genetic divergence between populations. In the plot, the y-axis represents *F*_ST_ values (0 to 1), while the x-axis displays genomic positions in megabase pairs (Mbp).

Principal component analysis (PCA) revealed genetic variation in *An. funestus* populations across Africa, with DIM1 and DIM2 explaining 23.5% and 17.8% of the variation, respectively (Fig. 4 and Fig. S6). Populations clustered broadly by geography, with Western (Ghana), Central (Cameroon), and Eastern (Uganda) populations closer to each other, while Southern (Malawi, FANG, and FUMOZ) populations formed a separate cluster (Fig. 4a and Fig. S6). Temporal changes were suggested by the distinct clustering of Cameroon and Malawi 2014 samples from 2021 populations, which could be due to distinct regional genomic changes over time. A Neighbour Joining tree showed similar patterns but inconsistencies, with Uganda Mayuge 2021 forming its own cluster (Fig. 4b).

### Temporal Variation in Genetic Differentiation Associated With Major Insecticide Resistance Loci in *An. funestus* Populations Across Africa

We conducted a temporal genomic analysis at two evolutionary time points (2014–2015 and 2021) to capture significant genomic changes linked with resistance escalation in *An. funestus* populations across Africa. *F_ST_* values (50,000 SNP windows) revealed two major resistance signals: the rp1 locus on chromosome 2R and the CYP9 locus on the X chromosome (Fig. 4c), both stronger in Cameroon (*F_ST_* ∼ 0.35 and *F_ST_* ∼ 0.4, respectively, (Fig. 4c). In contrast, weaker rp1 signals appeared in Ghana (*F_ST_* ∼ 0.04), Uganda (*F_ST_* ∼ 0.03 to 0.05), and Malawi (*F_ST_* ∼ 0.15; Fig. 4d, e). Ghana also showed a weak *CPR*-associated signal (∼14Mbp, X chromosome) (Fig. 4d).

### Contemporary Variation in Genetic Differentiation Associated With Insecticide Resistance Escalation in *An. funestus* Across Africa

**Fig. 5.**
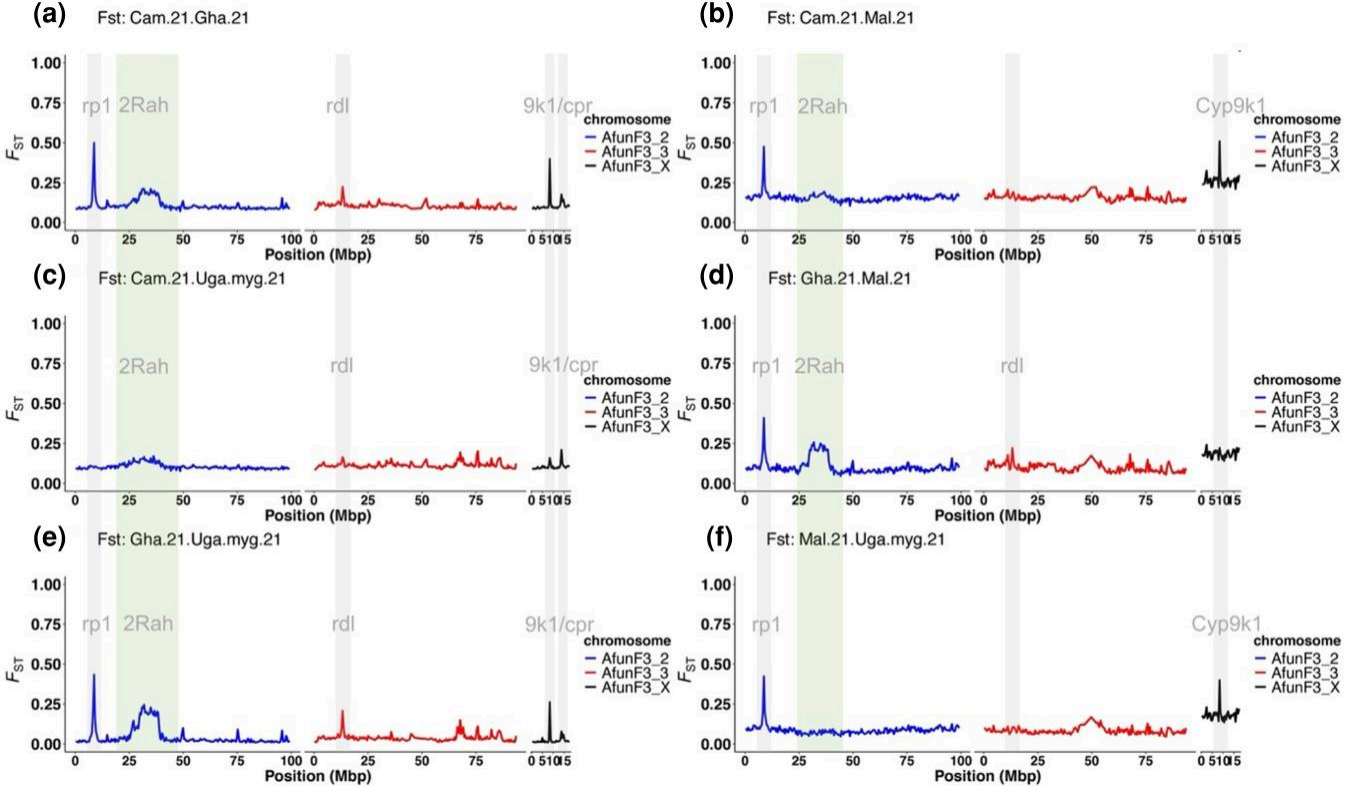
Contemporary variation in genetic differentiation of *An. funestus* populations across Africa. The plot presents a pairwise *F_ST_* genetic differentiation analysis of *An. funestus* mosquitoes collected in 2021 from Cameroon (Cam.21), Ghana (Gha.21), Uganda, and Malawi (Mal.21). *F_ST_* values were summarised in non-overlapping windows of 50,000 SNPs, considering only bi-allelic variants. F_ST_ values range from 0 to 1, with higher values indicating greater genetic divergence between populations. In the plot, the y-axis represents *F_ST_* values (0 to 1), while the x-axis displays genomic positions in megabase pairs (Mbp).

The intra-population genomic analysis, alongside temporal genomic variation, assessed samples from 2021 to evaluate current genetic variation associated with intense resistance across Africa. Six pairwise *F_ST_* comparisons in 50,000 SNP windows revealed four differentiated blocks (Fig. 5, highlighted in gray), centered around insecticide resistance loci *rp1*, *Cyp9*, *rdl*, and *CPR* shared across populations (Fig. 5). The *rp1* locus is a major genomic region associated with pyrethroid resistance in *An. funestus*, accounting for ∼87% of the resistance phenotype (Wondji et al. 2007). The *rdl* gene confers resistance to dieldrin, an insecticide that targets the GABA receptor, its primary site of action. *CYP9K1* is another important locus implicated in pyrethroid resistance, while *CPR* (Cytochrome P450 Reductase) encodes an electron donor that is essential for the activity of cytochrome P450 enzymes involved in metabolic resistance.

Additionally, we detected a 2Rah chromosomal inversion block on chromosome 2 (highlighted in green), as previously identified by (Boddé et al. 2025). This inversion is likely linked with behavioural and ecological adaptations.

At *rp1*, genetic differentiation was observed in all comparisons except Cameroon and Uganda (Mayuge), suggesting shared genetic material. *F_ST_* values ranged from ∼0.4 (Ghana vs. Malawi) to ∼0.5 (Cameroon vs. Ghana) (Fig. 5a, b, d–f). Signals around *Cyp9* locus on the X chromosome varied (*F_ST_*: 0.2 to 0.5) but were weak or absent in Ghana vs Malawi (Fig. 5d). A shared low-to-moderate signal in Cameroon, Ghana, and Uganda (*F_ST_* < 0.25) overlapped with *CPR*. Notably, a moderate signal (*F_ST_* ∼0.25) at *rdl* locus on chromosome 3 (∼13 Mbp) suggested resistance to dieldrin insecticide (Fig. 5a, d–e), even though dieldrin was banned decades ago. This persistence is likely explained by the role of *rdl* (GABA receptor) as a secondary target site for other insecticides, such as pyrethroids, which have been extensively used for vector control in the past decades (Bhatt et al. 2015; Taylor-Wells et al. 2015).

### Selective Sweeps Around Resistant-associated Loci in *An. funestus* Across Africa

**Fig. 6.**
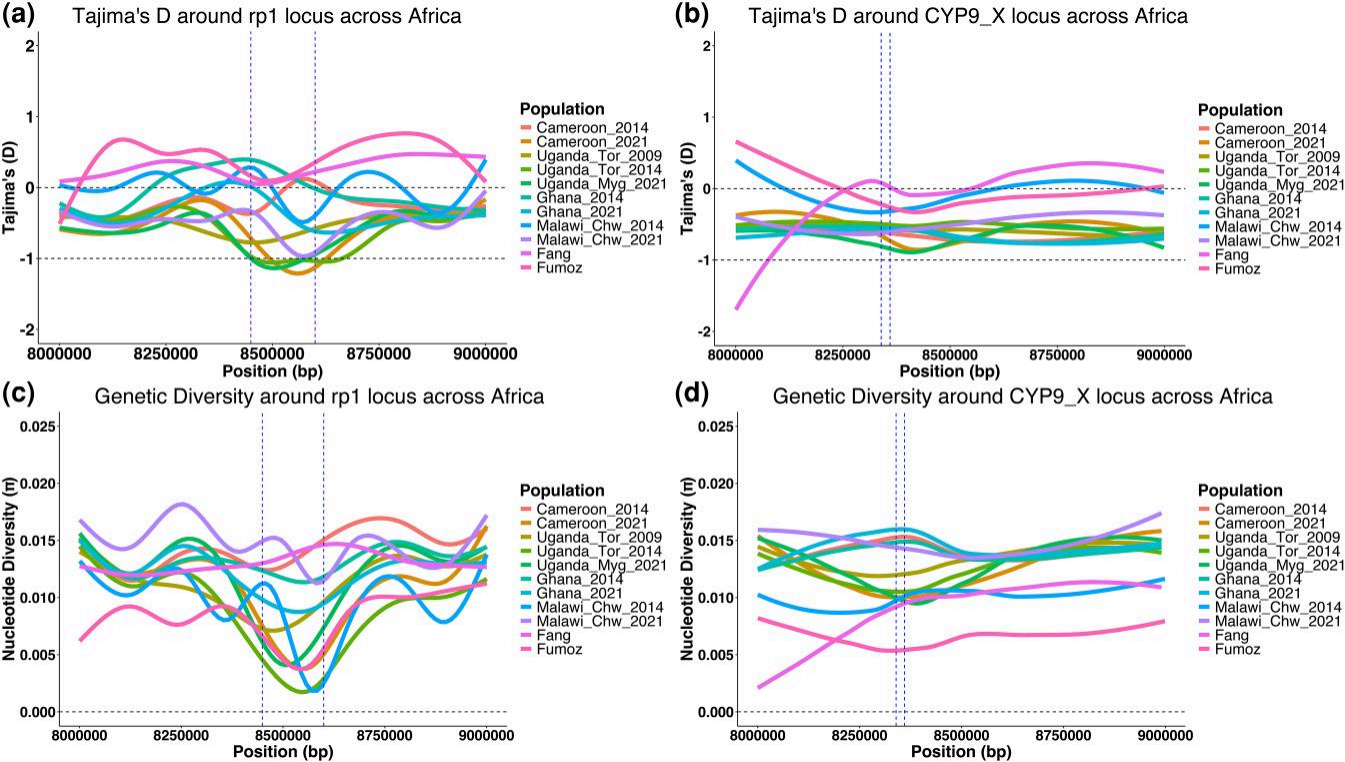
Selective sweeps around CYP6 and CYP9 clusters in *An. funestus* across Africa. (a) shows Tajima's D distribution across the CYP6 cluster with major selective sweep at the *rp1* in between the blue line, (b) shows Tajima's D distribution across the CYP9 cluster with major selective sweep around the *CYP9K1* gene in between the blue line, (c) shows nucleotide diversity distribution across the CYP6 cluster with major selective sweep at the rp1 in between the blue line and (d) shows nucleotide diversity distribution across the CYP9 cluster with major selective sweep where is located *CYP9K1* gene in between the blue line.

We focused our analysis on the differentiated *CYP6* and *CYP9* loci, calculating Tajima's D and nucleotide diversity to pinpoint selective sweeps. Subsampling at 20x coverage was performed across all populations to uniform coverage. In Cameroon, temporal divergence was linked to a decline in Tajima's D (Fig. 6a) and reduced genetic diversity at the *rp1* locus (Fig. 6c), likely indicating a positive selection and population expansion. Evidence of selection was also detected in Uganda (Mayuge 2021), Ghana (2021), and Malawi (2021), though weaker than in Cameroon. The *CYP9* locus showed similar patterns, with Cameroon 2021 and Uganda Mayuge 2021 exhibiting lower Tajima's D values around *CYP9K1* (Fig. 6b), leading to reduced diversity (Fig. 6c, d).

### Polymorphisms Associated With Resistant Loci in *An. funestus* Populations Across Africa

The SnpEff was used to annotate genetic variations in selective sweep regions (CYP6, CYP9, CPR, and GABA loci). Additionally, mutations in putative resistance genes (ace1, VGSC, GSTs cluster) were monitored, focusing on non-synonymous SNPs located (ns-SNPs) within the catalytic/active sites and/or substrate binding pocket.

### *CYP6* Locus

**Fig. 7.**
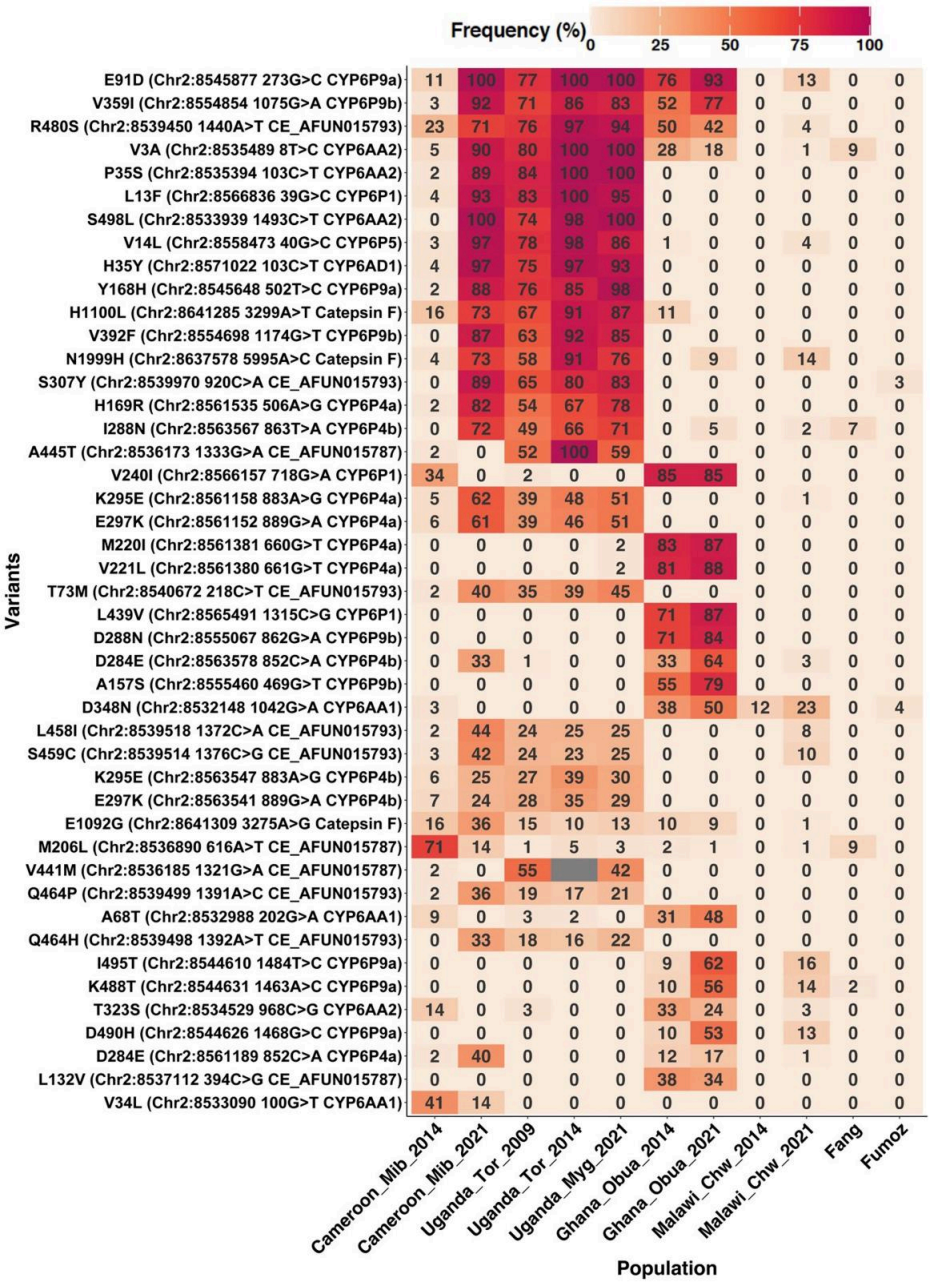
Mutations in the CYP6 locus potentially associated with resistance escalation in *an. funestus* across Africa. The heatmap displays key SNPs potentially associated with the escalation of insecticide resistance in *An. funestus* across Africa between 2014 and 2021. The numbers shown within the heatmap represent SNP allele frequencies for each population and year. Temporal variation in allele frequency across different countries is visualized using a yellow/white to dark red/purple color scale where yellow/white indicates low to moderate allele frequencies, and dark red/purple represents high frequencies, approaching or at fixation. The x-axis represents *An. funestus* populations sampled between 2009 and 2021, while the y-axis lists SNPs along with relevant annotations, including their genomic positions, corresponding chromosomes, associated genes, and the nucleotide and protein-level changes.

Within the *CYP6* locus, we observed regional variations in allelic frequencies of novel SNPs, including *CYP6P9a* (E91D, Y168), *CYP6P9b* (V359I, V392F), *CYP6AA2* (S498L), and others P450s-based SNPs (Fig. 7). These mutations approached fixation in Central, Eastern, and Western Africa but were absent in Southern Africa (Fig. 7). In Ghana, four mutations were identified, with *CYP6P9a* (E91D) and *CYP6P9b* (V359I) increasing over time (Fig. 7). Eight novel SNPs, including *CYP6P1* (V240I, L439V) and *CYP6P9b* (D288N, A157S), were unique to Ghana, suggesting specificity to Western *An. funestus*. Additionally, novel *CYP6P4a/b* SNPs (K295E, E297K) increased in frequency in Central (Cameroon) and Eastern Africa (Uganda) but were absent in Western and Southern Africa (Fig. 7). The lack of polymorphism in Malawi may have resulted from the use of the Southern African strain (FUMOZ) as the reference genome in this study. However, three amino acid changes specific to the Southern population (V109I, D335E, and N384S) were identified in the resistant *CYP6P9b* gene, representing key pyrethroid resistance mutations that conferred high metabolic efficiency (Ibrahim et al. 2015).

### *CYP9* Locus

Within the *CYP9* locus, the observed selection/differentiation was linked to the emergence of a unique SNP in the major cytochrome P450 gene, *CYP9K1* (G454A), recently associated with insecticide resistance across Africa (Djoko Tagne et al. 2025). This single amino acid change *CYP9K1* (G454A) was found at increasing frequencies over time, reaching fixation in Central and Eastern populations only (Table S5 and Fig. S8). This SNP was at lower frequency in Southern (Malawi) population, moving from 25% to 29% between 2014 and 2021, respectively. Several other SNPs were found at low frequencies but increasing over time in Western (Ghana) and Southern (Malawi) populations, (V142M, V327I, I242V, I242M, I110V).

### Cytochrome P450 Reductase (*CPR*)

We search SNPs around the *CPR* region found to be moderately differentiated in Ghana and Malawi populations from the *F_ST_* estimates. We found a major SNP (N70I) at higher frequencies specific only to Southern population but not elsewhere. This SNP, located near the active site of *CPR* has been found to have significantly increased between 2002 and 2021 in the Malawian *An. funestus* population (Table S5, Fig. S8). Many other mutations were found at increasing allelic frequencies though none exceeded 36% in any population, except in Uganda (Tororo), where SNP frequencies remained consistently low (ranging from 0% to 3%) (Table S5).

### Signatures of Complex Genomic Rearrangement Associated With Insecticide Resistance Escalation in *An. funestus* Across Africa

Structural variants calling around differentiated regions detected two mobile element (ME) insertions unique to Central and Eastern Africa (Figs. S8 and S9). The first is a 4.3 kb variant on chromosome 2R near *CYP6P9b*, was stable in Uganda (2014 to 2021) but emerged in Cameroon over time (2021) (Fig. S9**)**. A second ME, upstream of *CYP9K1* gene, showed similar patterns, but larger compared to the 4.3 kb (Fig. S10). At the *CYP6* locus, we identified seven duplications (DUP1-DUP7; Table S6). DUP5 (6.9 kb) and DUP3 (6.2 kb), spanning *CYP6AA1* and *CYP6P9b*, were specific to Ghana, with DUP5 increasing heterozygosity and DUP3 fixed since 2014. In Cameroon, DUP6 (16.9 kb) increased to fixation by 2021, while DUP4 (6.4 kb) remained stable. DUP1 (2.5 kb) and DUP2 (3.3 kb), common across Central, Eastern, and Western Africa, increased in Ghana and Cameroon but remained stable in Uganda (Figs. S11–S14). At the *CPR* locus, a novel 5.9 kb duplication spanning the 5′ region of the *CPR* gene emerged exclusively in Ghana. Absent in 2014, increased by 2021, indicating a rising genotype frequency (Table S6, Fig. S10).

### Targeted Analysis of Cytochrome P450 Reductase in *An. funestus* Across Africa Using PoolSeq, SureSelect, and Individual WGS

**Fig. 8.**
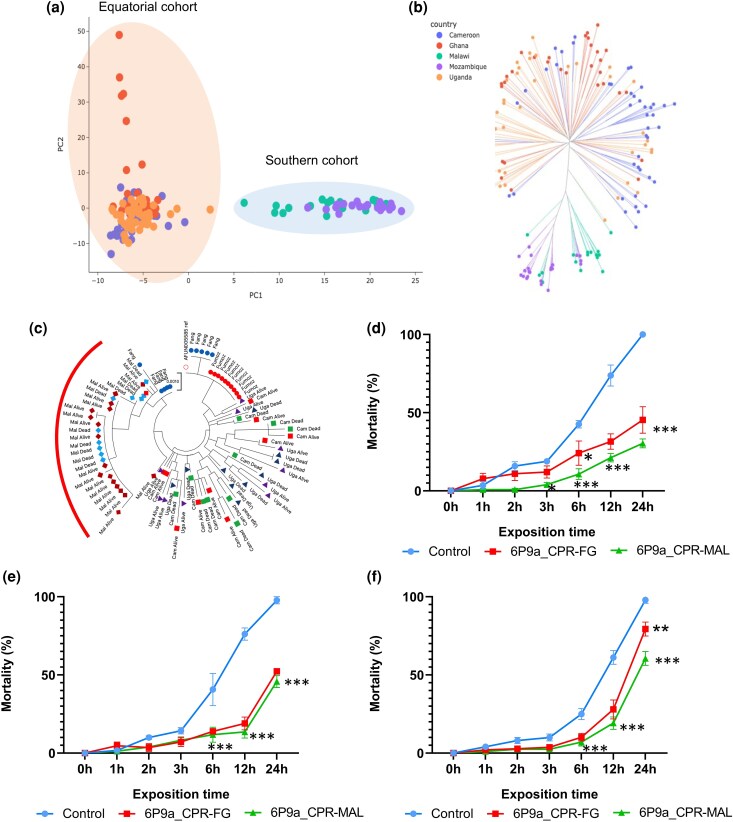
Diversity of the *CPR* gene in *An. funestus* across Africa and validation of the *CPR* (N70I) variant in insecticide resistance escalation. (a) Population structure analysis of *An. funestus* populations across Africa based on the *CPR* gene using iWGS data; (b) Neighbor-joining tree illustrating the genetic relationships among *An. funestus* populations across Africa; (c) Phylogenetic tree constructed from SureSelect data for *An. funestus* populations across the continent; (d, e, and f) are results from insecticide susceptibility bioassays using transgenic flies co-expressing *CYP6P9a* and the *CPR* (N70I) variant. These bioassays evaluate resistance to (d) permethrin, (e) deltamethrin, and (f) α-cypermethrin. Significance. codes: “***’ = *P* < 0.001; “**’ = *P* < 0.01; “*’ = *P* < 0.05.

Targeted analysis of the *CPR* gene was conducted using individual whole genome sequencing (iWGS) data obtained from the MalariaGEN data repository, as well as SureSelect data. The iWGS dataset included samples from Cameroon, Uganda, Malawi, Ghana (all from 2014), and Mozambique (2016), while the SureSelect dataset comprised sequences from alive and dead *Anopheles funestus* mosquitoes collected in 2014 from Cameroon, Uganda, Malawi, Fang, and FUMOZ (Supplementary text). PCA of *CPR* gene variants using iWGS using revealed distinct genetic backgrounds, with Malawi and Mozambique populations clustering together, while Cameroon, Ghana, and Uganda populations shared a separate genetic background (Fig. 8a, b; MalariaGEN *CPR* notebook). Phylogenetic analysis of the *CPR* gene using both iWGS and SureSelect data showed that the Malawi and Mozambique populations (iWGS data) were genetically distinct from the other populations. All Malawi samples (alive and dead) and the Mozambique samples clustered closely together, in broad agreement with their geographical origin, whereas the other more genetically diverse populations tended to cluster together with overlap (Fig. 8b, c). Genome-wide *H_12_* scans of samples collected between 2014 and 2018 identified strong selection signals at the *CYP9K1* gene in Uganda and the diacylglycerol kinase (*DGK*) gene across all populations except Malawi (Fig. S15) while weak selection at the *CPR* gene was observed in 2014 in Ghana and Malawi but not elsewhere (Fig. S15, purple block, Supplementary material). Using the 2014 Cameroon sample as a negative control, Pool-seq and RNA-seq analyses identified five divergent regions of varying intensity: *GSTT2*, *G-PCR*, *CYP9K1*, *DGK*, and *CPR* (Figs. S16–S18). Despite low divergence at the *CPR* locus across comparisons, a pronounced peak suggests starting or emerging selection, consistent with *H_12_* weak signals. However, RNA-seq analyses showed no significant differentiation in pairwise comparisons of alive at 1x, 5x, and 10x likely due to stronger field resistance (Fig. S19c–e).

Diplotype clustering at *CYP9K1*, *CPR*, and *DGK* genes suggests that SNPs (G454A in *CYP9K1*) and CNVs drive resistance in East Africa (Uganda and Kenya 2014), though the functional role of CNVs remains elusive (Figs. S20b and S21). In contrast, no CNV was found around *CPR* and *DGK* in all 2014 populations, but *CPR* contains a SNP (N70I) that drives a minor selective sweep observed in the Malawi population in 2014 (Figs. S20a and S22).

Genetic diversity analysis using SureSelect data showed high haplotype diversity (H_d_ ≈ 1) in most populations, except Malawi (H_d_ = 0.77–0.80) and FANG (H_d_ = 0.64). Nucleotide diversity (Pi) was low (≈0.004) across populations, with Malawi showing the lowest diversity (Pi = 0.0008). Segregating sites (S) were moderate for Cameroon and Uganda (27–41), minimal in Malawi (S = 4), and absent in FUMOZ (Tables S7 and S8). Neutrality tests suggested population expansion or purifying selection while phylogenetic analysis separated Malawi populations from others, highlighting their distinct genetic structure (Fig. 8c).

### Transgenic co-expression of *CPR* Alleles With the P450 *CYP6P9a*

To investigate whether the allelic variation observed between the mutant-type (*70I-CPR*) and wild-type (*N70-CPR*) alleles of *CPR* could influence insecticide resistance, both alleles were co-expressed with *CYP6P9A* in transgenic Drosophila using the GAL4/UAS system. Transgenic flies expressing *CYP6P9A-70I-CPR* (*CYP6P9A-MAL-CPR*) and *CYP6P9A-N70-CPR* (*CYP6P9A-FG-CPR*) were successfully generated under the control of the GAL4-Actin2 driver (Fig. S23).

### Co-expression of *CYP6P9A*/*CPR* and the Presence of 70I-*CPR* Increased Resistance to Pyrethroid in Transgenic Flies

#### Bioassays With Type I Pyrethroid

Bioassays with transgenic *Drosophila* expressing the mutant-type *CYP6P9A-MAL-CPR* allele revealed lower mortality rates (21% and 30% at 12 and 24 h, respectively) compared to flies expressing the wild-type *CYP6P9A-FG-CPR* allele (31% and 45% at 12 and 24 h, respectively). Control flies showed significantly higher mortality rates (73% and 100% at 12 and 24 h, respectively) (Fig. 8c). Notably, flies co-expressing *CYP6P9A* and the MAL-*CPR* mutant allele exhibited significantly lower mortality (30%) compared to those co-expressing *CYP6P9A* and the FG-*CPR* wild type allele (45%, *P* < 0.05) after 24 h of permethrin exposure (Fig. 8c). These findings demonstrate that cytochrome activity and resistance to permethrin are enhanced by the presence of the N70I*-CPR* mutation.

#### Bioassays With Type II Pyrethroids

Similar to permethrin, bioassay results with alpha-cypermethrin showed lower mortality in flies expressing the mutant *CYP6P9A-MAL-CPR* allele compared to those expressing the wild-type *CYP6P9A-FG-CPR* allele and control flies (Fig. 8f).

Indeed, transgenic flies expressing the *CYP6P9A*-70I-*CPR* allele exhibited significantly lower mortality rates following exposure to alpha-cypermethrin, with mortality rates of 20%, 22%, 26%, and 46% at 3, 6, 12, and 24 h, respectively. In comparison, flies expressing the *CYP6P9A-*N70*-CPR* allele had mortality rates of 29%, 34%, 35%, and 60% at the same time points, while control flies displayed significantly higher mortality rates of 41%, 46%, 55%, and 87% (Fig. 8f).

Statistical analysis revealed significant differences in mortality rates between flies expressing the wild-type *CYP6P9A-N70-CPR* allele and control flies (*P* < 0.05, *P* < 0.05, and *P* < 0.01 at 6, 12, and 24 h, respectively) (Fig. 8f). Furthermore, flies expressing the mutant *CYP6P9A-70I-CPR* allele showed even greater resistance, with mortality rates significantly lower than control flies (*P* < 0.01, *P* < 0.01, and *P* < 0.001 at 6, 12, and 24 h, respectively) (Fig. 8f).

Exposure of transgenic *Drosophila* to deltamethrin revealed significantly lower mortality rates in flies co-expressing the *CYP6P9A-70I-CPR* allele (mortality rates: 8%, 11%, 13%, and 45% at 3, 6, 12, and 24 h, respectively) and those co-expressing the *CYP6P9A-N70-CPR* allele (7%, 13%, 18%, and 52% at 3, 6, 12, and 24 h, respectively), compared to the control group (14%, 40%, 76%, and 97% at the same time points) (Fig. 8e).

Unlike permethrin and alpha-cypermethrin, no significant differences in mortality rates were observed between *Drosophila* expressing the *CYP6P9A-70I-CPR* allele and those expressing the *CYP6P9A-N70-CPR* allele when exposed to deltamethrin. This result suggests that the *N70I-CPR* allelic variation does not influence resistance to deltamethrin in *Drosophila*.

## Discussion

The rapid exacerbation of pyrethroid resistance poses a serious threat to current goals to reduce malaria burden by 50% in 2030 notably as all insecticide-treated nets still contain at least one pyrethroid insecticide. Deciphering the molecular factors driving this escalated resistance is crucial to manage and overcome its negative impact. In the context of intense resistance, integrating omics approaches is crucial, as it enhances the discovery of novel resistance mechanisms, variants and enables effective resistance management across the continent. Using this approach, we captured spatiotemporal signatures of evolutionary selection associated with insecticide resistance aggravation in wild-caught *Anopheles funestus* mosquitoes and functionally validated the role of a key *CPR* gene in resistance escalation across Africa.

### Novel Genes are Driving the Temporal Resistance Escalation

Our study identified several overexpressed genes in *Anopheles funestus* that were previously unlinked to insecticide resistance across Africa. Notably, the V-type proton ATPase subunit B emerged as the most consistently overexpressed gene. This gene, highly conserved in eukaryotes (Nelson et al. 2000), plays roles in disease processes (Collins and Forgac 2020), such as pathogen entry and cancer invasiveness in humans, and in processes like cuticle formation in insects (Zeng et al. 2021). It is an ortholog of *An. gambiae*'s V-type ATPase subunit B, linked to insecticide resistance in *An. coluzzii*. Post-transcriptional silencing of this gene significantly increased vector susceptibility (Ingham et al. 2018). Other overexpressed genes include Tubulin alpha-1 chain (*AFUN019762*), transposable element Tcb2, *CYP325B1*, Histone H3/4. Additionally, several putative insecticide resistance genes were overexpressed such as cytochrome P450s conferring resistance through metabolization of active ingredients (Mugenzi et al. 2019; Riveron et al. 2019; Wondji et al. 2022; Tatchou-Nebangwa et al. 2024), esterases (Nagi et al. 2024), odorant binding proteins, cuticular proteins, gustatory receptor genes. (Mugenzi et al. 2019). These findings highlight the importance of further validating the role of these novel targets such as the V-type ATPase B in insecticide resistance escalation across Africa.

### Chemosensory Proteins are Associated With Intense Resistance

Chemosensory proteins (CSPs) and sensorial appendage proteins (SAPs) exhibit increased expression in mosquitoes from Ghana and Uganda and those that survive 10 X pyrethroid across Africa, consistent with findings in *An. gambiae* and *An. coluzzii* from West Africa (Ingham et al. 2020). CSPs are directly implicated in pyrethroid resistance through transgenic studies and binding assays across arthropods (Xu et al. 2022). Similarly, overexpression of odorant-binding proteins (OBPs) has been linked to resistance in *An. gambiae* (Messenger et al. 2021). Although direct evidence for OBPs’ role in *Anopheles* resistance is lacking, knockdown of OBP28 in *Cx. quinquefasciatus* increases susceptibility to deltamethrin (Shen et al. 2022). These findings suggest that CSPs and SAPs play a critical role in driving behavioural and physiological adaptations that enhance insecticide resistance in *Anopheles* mosquitoes, particularly under extreme pyrethroid exposure. Functional validation of their role could provide new insights into combating pyrethroid-resistant malaria vectors. Increased expression of other genes such as MicroRNAs suggest post-transcriptional regulation, while MAPK mediates stress responses, and ornithine decarboxylase is linked to polyamine biosynthesis and resistance mechanisms, including resistance to anticancer drugs (Jang et al. 2017). Caspases have been associated with insecticide resistance in *Plutella xylostella* (Zhuang et al. 2011).

### Increased Expression of Cytochrome P450s is a Major Driver of Intense Resistance

Temporal transcriptomic analysis revealed a significant rise in *CYP9K1* expression from 2015/2014 to 2021 in Cameroon and Ghana, correlating with the emergence of the *CYP9K1* resistance locus in Cameroon. *CYP9K1* overexpression was more pronounced in Cameroon and linked to insecticide resistance escalation. A single missense variant (G454A) emerged and became fixed within 7 years, reducing diversity around *CYP9K1* in Central (Cameroon) and East (Uganda) Africa, likely spreading via gene flow. These findings emphasize *CYP9K1*'s role in pyrethroid resistance, driven by both overexpression and allelic variation (Djoko Tagne et al. 2025). Previously underexpressed P450 genes, such as *CYP6M2*, showed a temporal increase in expression, with significant overexpression observed across three regions and in mosquitoes surviving 10X insecticide doses. Moreover, the *CYP6P9a* tandem displayed exceptionally high expression in mosquitoes exposed to 10X the active ingredient. These findings suggest that increased overexpression of P450 genes is significantly contributing in the mosquitoes’ ability to survive extreme pyrethroid doses (Nguiffo-Nguete et al. 2023).

### Signatures of Selective Sweeps are Associated With Intense Resistance Escalation

Selection for insecticide resistance (IR) loci is widespread in contemporary *An. funestus* populations but appears more pronounced in Cameroon, showing significant temporal genomic changes between 2014 and 2021. This suggests the emergence of shared or independent haplotypes across regions. Nucleotide diversity (π) and Tajima's D analyses indicate selective sweeps and reduced genetic diversity, with some populations experiencing selection since 2014. Four key genomic regions, often associated with IR, were identified, including *CYP6 rp1*, *CYP9*, *GABA*, and *CPR* loci, all linked to insecticide resistance in *An. funestus* and *An. gambiae* (Dennis et al. 2024a). Genetic differentiation across regions reflects strong evolutionary selection, particularly in populations outside Cameroon, where resistance alleles or haplotypes may be nearing fixation. Temporal trends in genetic variations, including novel missense variants and structural variants were observed at the *rp1* and *CYP9* loci. These region-specific patterns of allele frequency variation suggest distinct local selective pressures. The identification of new and known candidate genes for pyrethroid resistance underscores the urgent need for genomic surveillance and alternative vector control strategies to prevent the spread of resistance, particularly in light of the 4.3 kb transposon-based structural variant in the *rp1* locus, which confers resistance across Central and East Africa (Mugenzi et al. 2024).

### Markers of Complex Evolution of Resistance Linked With Resistance Exacerbation

Our study identified signatures of complex genomic duplications within key cytochrome P450 genes within *rp1* locus. While two duplications (DUP1 and DUP2) were common across all populations except those from Southern Africa, most duplications were region-specific and have been previously described (Weedall et al. 2020). Some of these duplications, including DUP1 and DUP2, which span the 2X carboxylesterases and *CYP6P4a/b* paralog genes, show increasing frequencies over time, suggesting an ongoing selection process. Notably, the increase in the number of supportive reads over time suggests potential geographic genetic divergence and evolutionary history, as well as an increase in metabolic activity, as indicated by RNAseq findings.

These results highlight the challenge of linking these duplications directly to resistance phenotypes, though they provide strong evidence for their role in resistance escalation. This finding is consistent with previous reports on the importance of copy number variations (CNVs) in *An. gambiae*, *An. coluzzii*, and *An. arabiensis* populations across sub-Saharan Africa, where CNVs in genes like *CYP6AA1*/*CYP6AA2*, *GSTe*, *Cyp6z*, *CYP9K1*, and several esterases are associated with resistance to pyrethroids, carbamates, and organophosphates (Nagi et al. 2024). Further studies using long read sequencing (LRS) are necessary to fully decipher the mechanistic role of these duplications in insecticide resistance escalation.

### Allelic Variation of *CPR* is Increasing Pyrethroid Resistance Escalation

The *CPR* gene plays a pivotal role in the detoxification pathway by providing the necessary electrons to cytochrome P450 enzymes, which are directly involved in the metabolic breakdown of pyrethroid insecticides (Wang et al. 2025). Our findings demonstrate that allelic variation in *CPR*, specifically the N70I polymorphism, significantly enhances the metabolic capacity of P450s such as *CYP6P9a*, as evidenced by functional assays in transgenic *Drosophila*. This interaction underscores the synergistic effect between *CPR* and P450s, where changes in *CPR* can amplify the resistance phenotype conferred by P450s alone. The emergence and fixation of the 70I allele in Malawi populations, alongside a novel 5.9 kb promoter duplication in Ghana that likely upregulates *CPR* expression, suggest that both coding and regulatory changes in *CPR* can drive rapid resistance escalation. This polymorphism significantly emerged in the Malawi population *of An. funestus* between 2002 and 2021, initially absent. Recently, functional validation has been used to show that the presence of endogenous *An. funestus CPR* results in higher pyrethroid metabolism in vitro when co-expressed with *CYP6P9a* and *CYP6P9b*, in contrast to when these P450s are expressed with a surrogate *An. gambiae CPR* (Ibrahim et al. 2024). These findings are consistent with previous studies showing that *CPR* is associated with insecticide resistance in several insects (Gong et al. 2022 ; Wang et al. 2025). These molecular mechanisms highlight the complexity of resistance evolution and indicate that targeting *CPR*, in addition to P450s, may be necessary to reverse or attenuate resistance. From a policy perspective, these insights stress the urgency for genomic surveillance of both *CPR* and P450 loci and support the deployment of next-generation LLINs that combine multiple active ingredients or synergists to mitigate resistance spread and preserve vector control efficacy.

Additionally, a novel 5.9 kb duplication was observed spanning the promoter region of the *CPR* gene, specific to the Ghanaian population. This duplication was absent in the 2014 samples but consistently emerged in 2021. The duplication is believed to increase the activity of gene regulatory elements or transcription factor binding sites in the promoter region, potentially upregulating *CPR* expression and contributing to increased resistance in Ghana population. *CPR* overexpression has been previously implicated in insecticide resistance in other species, such as *Culex quinquefasciatus* and *An. gambiae* (Gong et al. 2022), as well as in *Triatoma infestans* (Varela et al. 2024). Future studies examining the entire *CPR* gene will help elucidate the relationship between promoter duplication and gene regulation in intense resistance.

## Conclusion

Our study provides the first comprehensive spatiotemporal analysis of intense resistance in *An. funestus* across Africa, identifying novel overexpressed genes, variants, and a *CPR*-mediated intense resistance mechanism in Southern African. Regional DNA markers and novel variants were detected, highlighting their potential for genomic surveillance and resistance management.

## Materials and Methods

### Study Site and Mosquito Collections

Blood-fed, wild female *Anopheles* mosquitoes, resting indoor on the walls and roofs of houses, were collected in 2021 using an electric Prokopack aspirator (John W. Hock co., USA), in four countries spanning 4 distinct regions of sub-Saharan Africa: Cameroon (Central Africa, Mibellon: 6°46′N, 11°70′E), Uganda (East Africa, Tororo: 0°41′N, 34°10′E and Mayuge: 0°23′10.8″ N, 33°37′16.5″ E), Ghana (West Africa, Atatam: 06° 17.377′′ N, 001° 27.545′′ W), and Malawi (Southern Africa, Chikwawa: 16° 2′ 8′′S, 34° 50′ 21′′E) (Fig. S24 and Table 1).

**Table 1.** Study design for the RNAseq experiments.

RNAseq temporal analysis design			RNAseq dose response analysis design ^a^		
Uganda 2014	vs.	FANG 2014	Unexposed	vs.	FANG 2023
Ghana 2014	vs.	FANG 2014	Perm 1x	vs.	FANG 2023
Malawi 2014	vs.	FANG 2014	Perm 5x	vs.	FANG 2023
Cameroon 2015	vs.	FANG 2014	Perm 10x	vs.	FANG 2023
Uganda 2021	vs.	FANG 2023	Perm 1x	vs.	Unexposed
Ghana 2021	vs.	FANG 2023	Perm 5x	vs.	Unexposed
Malawi 2021	vs.	FANG 2023	Perm 10x	vs.	Unexposed
Cameroon 2021	vs.	FANG 2023	Perm 10x	vs.	Perm 1x
Uganda 2021	vs.	Uganda 2014	Perm 10x	vs.	Perm 5x
Ghana 2021	vs.	Ghana 2014	Perm 5x	vs.	Perm 1x
Malawi 2021	vs.	Malawi 2014			
Cameroon 2021	vs.	Cameroon 2015			

^a^Malawian mosquito samples (susceptibility profiling can be found in Menze et al., 2022).

All collected *Anopheles* mosquitoes were morphologically identified as belonging to either the *An. funestus* group or *An. gambiae* complex using morphological keys (Coetzee 2020). Blood-fed mosquitoes were kept in paper cups and fed with 10% sugar solution for 4 to 5 d to allow them to become fully gravid. Subsequently, they were induced to lay eggs in 1.5 ml Eppendorf tubes as previously described (Morgan et al. 2010) and were reared till adult (F_1_ generation) stage for WHO bioassay testing (WHO 2016). WHO tube assays showing consistent and temporal increases in resistance to pyrethroids including permethrin, deltamethrin and alphacypermethrin in 2021 have been documented across all study sites (Mulamba et al. 2014; Riveron et al. 2019; Tchouakui et al. 2021; Menze et al. 2022; Mugenzi et al. 2022; Tazokong et al. 2024) with mortalities ranging from <64% to <90% at 10x diagnostic concentration.

A subset of non-oviposited F_0_ females from each country was preserved for subsequent spatiotemporal genomic studies in 1.5 ml Eppendorf tubes containing either silica gel or RNA later. *Anopheles funestus* was molecularly identified as the major malaria vector in all the study sites across the four countries (Mulamba et al. 2014; Riveron et al. 2015, 2016; Menze et al. 2018; Tchouakui et al. 2021; Menze et al. 2022; Mugenzi et al. 2022).

### RNA and gDNA Extraction, Library Preparation and Sequencing

Two RNA-Seq experiments were conducted: one with unexposed F_1_ samples from four countries, and the other with Malawi F_1_ samples which survived permethrin exposure at 1X, 5X, and 10X concentration (<69% mortality), reflecting the highest resistance intensity (Menze et al. 2022). RNA isolation included 3–4 biological replicates of 10 individuals per pool from each country. RNA was extracted from 3–5-d-old unfed female *An. funestus* using the Arcturus PicoPure RNA Isolation Kit (Life Technologies, Carlsbad, CA, USA). Four pools of the multiple insecticide susceptible lab strain FANG (2023 batch) were included as well. RNA was purified with DNase I, quantified using Nanodrop (Thermo Fisher, MA, USA) and Qubit 4 fluorometer (Invitrogen^TM^, Thermo fisher scientific) while the quality check was assessed using Tape Station 4150 (Agilent technologies). For Pool-Seq GWAS, 40 *An. funestus* F_0_ mosquitoes were pooled per country to capture genomic changes even at low coverage (Boitard et al. 2012; Barnes et al. 2017; Weedall et al. 2020; Dennis et al. 2024b). Individual genomic DNA was extracted with the DNeasy kit (Qiagen 2020), pooled, purified with RNase A and quantified. Both RNA and DNA from 2021 populations including FANG 2023 were subjected to library preparation, quality control, and 2 × 150 bp pair-end sequencing by Novogene (Cambridge, UK) using NovaSeq 6000.

RNAseq and PoolSeq data generated previously from samples collected back to 2009 and 2014–2015 (table S9) were obtained from all four countries (Weedall et al. 2019; Weedall et al. 2020), facilitating temporal analyses of transcriptional and genomic changes in *An. funestus* across its range. Mosquitoes from this temporal data were collected, reared and extracted as described above but sequenced on Illumina Hiseq2500 (2 × 125-bp paired-ends) by the Centre for Genomic Research (CGR), University of Liverpool (Weedall et al. 2019, 2020).

### Analysis of RNAseq Data

The de-multiplexed fastq files were trimmed using Cutadapt (version 1.2.1) (Martin 2011) and low-quality bases were trimmed with Sickle (version 1.2) (Joshi and Fass 2011). RNA-Seq data was processed as previously described (Wondji et al. 2022) with Strand NGS (version 3.4) (Strand Life Sciences, Bangalore, India), aligning reads to the *An. funestus* reference (Afun 3.1) and raw reads counts table generated using featurecounts. Normalization and differential gene expression (DGE) analysis were conducted following DESeq2 pipeline (Love et al. 2014).

Two experiments were conducted including multiple pairwise comparisons (Table 1). In the first experiment, intra-population pairwise DGE analysis was performed across temporal unexposed samples from different countries, including FANG replicates. To account for batch effect and noise introduced by sequencing technology, sequencing and read depths comparison were made between each population and FANG sequenced at the same time. The log_2_ fold-change (log2FC) for each gene was calculated using a moderated *t*-test. *P*-values were adjusted for multiple testing using the false discovery rate (FDR, Benjamini Hochberg) (Benjamini and Hochberg 1995). Genes were considered as differentially expressed if the FDR was <0.05 and |FC| ≥ 2 for pairwise between each population (2014–2015 and 2021) versus its respective batch of susceptible FANG (2014 and 2023) while cutoff of FDR < 0.05 and FC ≥ 1.5 was set for 2021 versus 2014–2015 populations (Table 1). This enables the identification of gene temporally, uniquely and commonly overexpressed across the four countries.

To further elucidate the molecular mechanisms by which *Anopheles* mosquitoes respond and adapt to increasing insecticide pressure, we focused on the transcriptional profiles of mosquitoes surviving exposure to permethrin 5X and 10X the diagnostic dose (DD) against those surviving the 1X DD.

In the second experiment with Malawi samples, three approaches were used to identify differentially expressed genes (DEGs) (Table 1). The first compared all alive phenotypes (permethrin 1X, 5X, and 10X) to the susceptible FANG strain (R vs S) with |FC| ≥ 2 and FDR < 0.05. The second compared all alive phenotypes to unexposed samples (R vs C) with cutoff of FC ≥ 1.5 and FDR < 0.05. The third involved pairwise comparisons of resistant 1X, 5X and 10X exposed samples (R_5X vs R_1X, R_10X vs R_1X, and R_10X vs R_5X) with DEGs filtered at FC ≥ 1.5 and FDR < 0.05 (Table 1). Beside these thresholds for upregulated genes, the normalized raw reads counts were considered to strengthen the pattern observed.

To have a better insight of the metabolic processes or metabolic pathways that are activated due to the insecticide exposure, a gene ontology (GO) pathway enrichment analysis was performed using gene commonly overexpressed across temporal and dose response analyses. This list of upregulated genes was subjected to gprofiler (https://biit.cs.ut.ee/gprofiler/gost) (Reimand et al. 2007) with enrichment cutoff set at FDR < 0.05 and the resulting GO table was downloaded to generate bar plot of term enrichment from the three categories (molecular function, biological process and cellular component). All the plots from RNAseq analysis were generated and visualized in R version 4.4.2 with the ggplot2 package.

### Pool-Seq Whole Genome Sequencing Data Analysis

Quality assessment, mapping, and filtering were performed using the Pool-Seq pipeline as previously described (Gadji et al. 2024) and available on GitHub (https://github.com/Gadji-M/PoolSeq_OMIcsTouch). Fastq files quality was assessed with FastQC and Multiqc (Ewels et al. 2016). Reads were trimmed with Trimmomatic (Bolger et al. 2014) and aligned based on *An. funestus* reference genome (release 61) sourced from VectorBase (www.vectorbase.org) using BWA via the “Alignment.sh’ script (Li 2013). The aligned BAM files were sorted and duplicates marked using Picard tools (https://broadinstitute.github.io/picard/). A synchronized file for PoPoolation2 (Kofler et al. 2011b) analysis was generated with “mpileup2sync.jar’ after creating a pileup file using “samtools mpileup’ (Danecek et al. 2021) command.

### Population Structure and Genomic Analysis

A PCA analysis was conducted to infer the population structure of *An. funestus* populations across the four countries representing Central, Western, Eastern and Southern Africa. Allele frequency was estimated using the “snp-frequency-diff.pl’ at each locus and was used to analyse population history (Kofler et al. 2011b). The parameters used were: –min-count 2 –min-coverage 10 –max-coverage 5% to ignore the highest coverages, independently estimated for every pooled population. Moreover, correlation plot and Neighbor-Joining tree were generated using genome-wide pairwise *F_ST_* values between each pooled population in Poolfstat and ape packages (Gautier et al. 2022). The custom script “Fst_sliding_windows.sh’, which implements features of Popoolation2 such as fst-sliding.pl (Kofler et al. 2011b), was utilized to calculate intra- and inter-population pairwise *F_ST_* genetic differentiation across the genome in non-overlapping windows ranging from 5 to 50 kb using the synchronized file as input. Furthermore, the *F_ST_* files were formatted and visualized in R using the ggplot2 package (Wickham and Wickham 2016).

### Variant Calling and Diversity Analysis

Variant calling was performed using VarScan, (Koboldt et al. 2009, 2013) then annotated and filtered with SnpEff (Cingolani et al. 2012) and VCF file was filtered using SnpSift and bcftools (Cingolani et al. 2012; Danecek et al. 2021). To determine possible selective sweeps in our populations, genome and loci-wide Tajima's D and nucleotide diversity (π) were computed for each population in Popoolation1 (Kofler et al. 2011a) after subsampling without replacement to uniform the coverage to 20x for all populations. This was done in overlapping windows of 50 kb moving in steps of 25 kb, then by zooming into differentiated loci using windows of 1 kb, as indicated by prominent peaks from the *F_ST_* plots.

### Detection of Signatures of Complex Genomic Rearrangements

Comprehensive analyses of genomic rearrangements were conducted using INSurVeyor for large insertions (>1 kb) (Rajaby et al. 2023) and Smoove for duplications, deletions, and inversions calling (https://github.com/brentp/smoove). The “python3 insurveyor.py’ command was run on each BAM file, and VCF files were merged with SurVClusterer (https://github.com/Mesh89/SurVClusterer). Additional variant calling in Smoove used the “smoove call’ command, and variants were annotated with “smoove annotate’. Structural variants were filtered with “bcftools filter’ (Danecek et al. 2021). Structural variants (SVs) and BAM files were visualized in Integrative Genomic Viewer (IGV) for confirmation of key SVs and quality assessment and detection of complex variants and breakpoints. Key metrics for identifying genomic anomalies included coverage depth, insert sizes, read pair orientations, and chimeric reads.

### Targeted Analysis of a Temporally Selected Variant of the Cytochrome P450 Reductase (*CPR*) in Southern Africa

#### Polymorphisms Analysis of *CPR* Gene Across Africa Using MalariaGEN Data

Targeted analysis of the *CPR* gene was conducted to capture genetic diversity and identify major variants associated with resistance escalation in *An. funestus* across five countries (Cameroon, Uganda, Malawi, Ghana, and Mozambique). This analysis was performed using the MalariaGEN Python package on the Colab platform, leveraging the Af1 API documentation. Principal Component Analysis (PCA) was carried out across these five populations based on the *CPR* gene, followed by identification of genomic regions on the X chromosome undergoing recent positive selection. These regions were further characterized through genome-wide *H_12_* scans and pairwise *F_ST_* statistics in non-overlapping windows of 1,000 SNPs (5,000 SNPs for the Malawi population). Additionally, diplotype clustering analysis was performed within these loci to determine whether copy number variations (CNVs), single nucleotide polymorphisms (SNPs), or both contribute to resistance escalation (Nagi et al. 2024).

### *In Vivo* Functional Validation of the Role of Malawi CPR Using Transgenic Flies

#### Cloning and Construction of Dual Transgenic Expression Plasmids

To investigate the role of the N70I-*CPR* mutation on insecticide resistance, transgenic Drosophila melanogaster flies co-overexpressing *An. funestus CYP6P9a* together with Malawi *CPR* (70I mutant) and *CYP6P9a* together with lab susceptible FANG *CPR* (wild type N70) alleles were generated using a GAL4-actinII-UAS system. Briefly, co-expression of *CYP6P9a*-N70-*CPR* and *CYP6P9a*-70I-*CPR* in *Drosophila* was achieved by using short viral peptide sequences (P2A) that mediated a ribosomal skipping event allowing multiple separate peptide products to be generated from a single expression vector, as previously described (Daniels et al. 2014). The CYP6P9a-P2A-MAL-*CPR* and *CYP6P9a*-P2A-FG-*CPR* were purified using the Qiagen gel extraction kit (Qiagen, Hilden, Germany) and cloned into the Drosophila expression vector pUAS-C5-attb, which was pre-digested with the same restriction enzyme to generate pUAS-C5-attb::*CYP6P9a*-P2A-MAL-*CPR* and pUAS-C5-attb::*CYP6P9a*-P2A-FG-CPR as previously describe (González et al. 2011; Riveron et al. 2014). Briefly, *CYP6P9a*-P2A-MAL-*CPR* and *CYP6P9a*-P2A-FG-*CPR* were ligated into the pUASC5-attb vector using the T4 ligase kit. After ligation, the constructs were transformed in *E. coli* DH for 16 h at 37 °C. To confirm the co-expression of *CYP6P9a* and Mal-*CPR*/FG-*CPR* in the same construct with factor P2A, positive colonies were purified after screen colony PCR and sent for sequencing. The resulting pUASC5-attb::*CYP6P9a*-P2A-MAL-*CPR* and pUASC5-attb::*CYP6P9a*-P2A-FG-*CPR* constructs were purified using the Midipprep kit (Qiagen, Hilden, Germany) and sent to the Cambridge flies facility (https://www.flyfacility.gen.cam.ac.uk/) for injection into the germ-line of *D. melanogaster* carrying the attP40 docking site on chromosome 2 (y1 w67c23; P (CaryP) attP40,1;2) using the PhiC31 system (Markstein et al. 2008). Ubiquitous co-expression of UAS::*CYP6P9A*-P2A-MAL-*CPR* and UAS::*CYP6P9a*-P2A-FG-*CPR*, were obtained in the flies by crossing them with the driver line, Act5C-GAL4 strain (y1 w*; P (Act5C-GAL4-w) E1/CyO,1;2) (Bloomington Stock Center, IN, USA). Flies without UAS insert (white eyes) were also crossed with the Act5C-GAL4 line for the control line.

### Validation of co-expression of Transgenes in *Drosophila*

The expression of recombinant *CYP6P9a-70I-CPR* and *CYP6P9a-N70-CPR* in the experimental flies was confirmed through real time semi-quantitative PCR as described previously (Ibrahim et al. 2015; Kouamo et al. 2025). Total RNA was extracted from tree pools of five flies from each transgenic line and control from F_1_ generation before insecticide bioassays, as previously described (Kouamo et al. 2021) and the cDNA was synthetized. Semi-quantitative qRT-PCR was conducted using *CYP6P9a* and *CPR* primers (primers listed in table S10, Supplementary material) to evaluated co-expression of *CYP6P9a* and *CPR* in both *CYP6P9a*-Mal-CPR and *CYP6P9a*-FG-*CPR* and the absence of the expression of those gene in control flies.

### Determination of Insecticides Susceptibility Using Contact Bioassays

F_1_ progenies (2–4-d old females) overexpressing *CYP6P9a-MAL-CPR* and *CYP6P9a-FG-CPR* were exposed to insecticides as described in (Riveron et al. 2014). The transgenic and control flies were exposed to permethrin (2%), alpha-cypermethrin (0.0007%) and deltamethrin (0.2%) following the protocol in (Riveron et al. 2013). Bioassays included five replicates of 20–25 flies each, with mortality and knockdown scored at 1, 2, 3, 6, 12, and 24 h. Mortality and knockdown rates were compared between experimental and control groups using Student's *t*-test. When the mortality rate of transgenic flies is significantly lower than that of the control line, we can conclude that the transgenic line is more resistant to insecticide. This suggests that the expression of the candidate gene, or the presence of allelic variation, contributes to enhance insecticide resistance.

## Supplementary Material

msaf251_Supplementary_Data

## Data Availability

The datasets derived from the 2021 PoolSeq and RNAseq sequencing are accessible on the European Nucleotide Archive under the accession numbers PRJEB84919 and PRJEB84920, respectively. Pooled template whole genome sequencing and RNAseq data from 2014 used for temporal analyses are available under study accessions PRJEB13485 (Malawi 2014) and PRJEB24384 (Cameroon, Ghana and Uganda 2014) and RNAseq data (PRJEB24351 and PRJEB10294). iWGS data can be accessed through the malariaGEN_data python package at https://malariagen.github.io/malariagen-data-python/v15.3.0/Af1.html. All analysis codes utilized in this study are described and accessible within the GitHub repository via https://github.com/Gadji-M/PoolSeq_OMIcsTouch.

## Data Availability

The datasets derived from the 2021 PoolSeq and RNAseq sequencing are accessible on the European Nucleotide Archive under the accession numbers PRJEB84919 and PRJEB84920, respectively. Pooled template whole genome sequencing and RNAseq data from 2014 used for temporal analyses are available under study accessions PRJEB13485 (Malawi 2014) and PRJEB24384 (Cameroon, Ghana and Uganda 2014) and RNAseq data (PRJEB24351 and PRJEB10294). iWGS data can be accessed through the malariaGEN_data python package at https://malariagen.github.io/malariagen-data-python/v15.3.0/Af1.html. All analysis codes utilized in this study are described and accessible within the GitHub repository via https://github.com/Gadji-M/PoolSeq_OMIcsTouch.

## References

[msaf251-B1] Balabanidou V et al Cytochrome P450 associated with insecticide resistance catalyzes cuticular hydrocarbon production in Anopheles gambiae. Proc Natl Acad Sci. 2016:113:9268–9273. 10.1073/pnas.1608295113. 27439866 PMC4995928

[msaf251-B2] Balabanidou V, Grigoraki L, Vontas J. Insect cuticle: a critical determinant of insecticide resistance. Curr Opin Insect Sci. 2018:27:68–74. 10.1016/j.cois.2018.03.001. 30025637

[msaf251-B3] Barnes KG et al Genomic footprints of selective sweeps from metabolic resistance to pyrethroids in African malaria vectors are driven by scale up of insecticide-based vector control. PLoS Genet. 2017:13:e1006539. 10.1371/journal.pgen.1006539. 28151952 PMC5289422

[msaf251-B4] Benjamini Y, Hochberg Y. Controlling the false discovery rate: a practical and powerful approach to multiple testing. J R Stat So: Ser B (Methodological). 1995:57:289–300. 10.1111/j.2517-6161.1995.tb02031.x.

[msaf251-B5] Bhatt S et al The effect of malaria control on Plasmodium falciparum in Africa between 2000 and 2015. Nature. 2015:526:207–211. 10.1038/nature15535. 26375008 PMC4820050

[msaf251-B6] Boddé M et al Genomic diversity of the African malaria vector *Anopheles funestus*. Science. 2025:389:eadu3596. 10.1126/science.adu3596. 40966334

[msaf251-B7] Boitard S, Schlötterer C, Nolte V, Pandey RV, Futschik A. Detecting selective sweeps from pooled next-generation sequencing samples. Mol Biol Evol. 2012:29:2177–2186. 10.1093/molbev/mss090. 22411855 PMC3424412

[msaf251-B8] Bolger AM, Lohse M, Usadel B. Trimmomatic: a flexible trimmer for Illumina sequence data. Bioinformatics. 2014:30:2114–2120. 10.1093/bioinformatics/btu170. 24695404 PMC4103590

[msaf251-B9] Chung H et al Cis-regulatory elements in the accord retrotransposon result in tissue-specific expression of the Drosophila melanogaster insecticide resistance gene Cyp6g1. Genetics. 2007:175:1071–1077. 10.1534/genetics.106.066597. 17179088 PMC1840086

[msaf251-B10] Cingolani P et al A program for annotating and predicting the effects of single nucleotide polymorphisms, SnpEff: SNPs in the genome of Drosophila melanogaster strain w1118; iso-2; iso-3. Fly (Austin). 2012:6:80–92. 10.4161/fly.19695. 22728672 PMC3679285

[msaf251-B11] Coetzee M . Key to the females of Afrotropical Anopheles mosquitoes (Diptera: Culicidae). Malar J. 2020:19:70. 10.1186/s12936-020-3144-9. 32054502 PMC7020601

[msaf251-B12] Collins MP, Forgac M. Regulation and function of V-ATPases in physiology and disease. Biochim Biophys Acta Biomembr. 2020:1862:183341. 10.1016/j.bbamem.2020.183341. 32422136 PMC7508768

[msaf251-B13] Danecek P et al Twelve years of SAMtools and BCFtools. GigaScience. 2021:10:giab008. 10.1093/gigascience/giab008. 33590861 PMC7931819

[msaf251-B14] Daniels RW, Rossano AJ, Macleod GT, Ganetzky B. Expression of multiple transgenes from a single construct using viral 2A peptides in Drosophila. PLoS One. 2014:9:e100637. 10.1371/journal.pone.0100637. 24945148 PMC4063965

[msaf251-B15] Dennis TPW et al Signatures of adaptation at key insecticide resistance loci in Anopheles gambiae in Southern Ghana revealed by reduced-coverage WGS. Sci Rep. 2024a:14:8650. 10.1038/s41598-024-58906-x. 38622230 PMC11018624

[msaf251-B16] Dennis TPW et al Cryptic population structure and insecticide resistance in Anopheles gambiae from the southern Democratic Republic of Congo. Sci Rep. 2024b:14:21782. 10.1038/s41598-024-70885-7. 39294180 PMC11410927

[msaf251-B17] Djoko Tagne CS et al A single mutation G454A in the P450 CYP9K1 drives pyrethroid resistance in the major malaria vector Anopheles funestus reducing bed net efficacy. Genetics. 2025:229:1–40. 10.1093/genetics/iyae181. PMC11708915 39509710

[msaf251-B18] Ewels P, Magnusson M, Lundin S, Käller M. MultiQC: summarize analysis results for multiple tools and samples in a single report. Bioinformatics. 2016:32:3047–3048. 10.1093/bioinformatics/btw354. 27312411 PMC5039924

[msaf251-B19] Fu B et al GPCR-MAPK signaling pathways underpin fitness trade-offs in whitefly. Proc Natl Acad Sci U S A. 2024:121:e2402407121. 10.1073/pnas.2402407121. 38959045 PMC11252912

[msaf251-B20] Gadji M et al Genome-wide association studies unveil major genetic loci driving insecticide resistance in Anopheles funestus in four eco-geographical settings across Cameroon. BMC Genomics. 2024:25:1202. 10.1186/s12864-024-11148-7. 39695386 PMC11654272

[msaf251-B21] Gadji M et al 2025. PoolSeq genome-wide association studies and microbial signature analyses identify novel candidates associated with pyrethroid resistance evolution in Anopheles funestus in Cameroon [version 1]. VeriXiv 2.

[msaf251-B22] Gautier M, Vitalis R, Flori L, Estoup A. f-Statistics estimation and admixture graph construction with Pool-Seq or allele count data using the R package poolfstat. Mol Ecol Resour. 2022:22:1394–1416. 10.1111/1755-0998.13557. 34837462

[msaf251-B23] Gong Y, Li T, Li Q, Liu S, Liu N. The central role of multiple P450 genes and their co-factor CPR in the development of permethrin resistance in the mosquito *Culex quinquefasciatus*. Front Physiol. 2022:12:802584. 10.3389/fphys.2021.802584. 35095564 PMC8792746

[msaf251-B24] González M et al Generation of stable Drosophila cell lines using multicistronic vectors. Sci Rep. 2011:1:75. 10.1038/srep00075. 22355594 PMC3216562

[msaf251-B25] Hemingway J . The way forward for vector control. Science. 2017:358:998–999. 10.1126/science.aaj1644. 29170222

[msaf251-B26] Ibrahim SS et al Allelic variation of cytochrome P450s drives resistance to bednet insecticides in a Major malaria vector. PLoS Genet. 2015:11:e1005618. 10.1371/journal.pgen.1005618. 26517127 PMC4627800

[msaf251-B27] Ibrahim SS et al Molecular drivers of insecticide resistance in the Sahelo-Sudanian populations of a major malaria vector Anopheles coluzzii. BMC Biol. 2023:21:125. 10.1186/s12915-023-01610-5. 37226196 PMC10210336

[msaf251-B28] Ibrahim SS et al Functional validation of endogenous redox partner cytochrome P450 reductase reveals the key P450s CYP6P9a/-b as broad substrate metabolizers conferring cross-resistance to different insecticide classes in *Anopheles funestus*. Int J Mol Sci. 2024:25, 8092. 10.3390/ijms25158092. 39125661 PMC11311542

[msaf251-B29] Ingham VA et al A sensory appendage protein protects malaria vectors from pyrethroids. Nature. 2020:577:376–380. 10.1038/s41586-019-1864-1. 31875852 PMC6974402

[msaf251-B30] Ingham VA, Wagstaff S, Ranson H. Transcriptomic meta-signatures identified in Anopheles gambiae populations reveal previously undetected insecticide resistance mechanisms. Nat Commun. 2018:9:5282. 10.1038/s41467-018-07615-x. 30538253 PMC6290077

[msaf251-B31] Jang W-J et al Multi-omics analysis reveals that ornithine decarboxylase contributes to erlotinib resistance in pancreatic cancer cells. Oncotarget. 2017:8:92727–92742. 10.18632/oncotarget.21572. 29190951 PMC5696217

[msaf251-B32] Joshi NA, Fass J. Sickle: A sliding-window, adaptive, quality-based trimming tool for FastQ files. [Software]. Version 1.33. 2011.

[msaf251-B33] Koboldt DC et al VarScan: variant detection in massively parallel sequencing of individual and pooled samples. Bioinformatics. 2009:25:2283–2285. 10.1093/bioinformatics/btp373. 19542151 PMC2734323

[msaf251-B34] Koboldt DC, Larson DE, Wilson RK. Using VarScan 2 for germline variant calling and somatic mutation detection. Curr Protoc Bioinformatics. 2013:44:15.4.1–15.4.17. 10.1002/0471250953.bi1504s44. PMC4278659 25553206

[msaf251-B35] Kofler R et al Popoolation: a toolbox for population genetic analysis of next generation sequencing data from pooled individuals. PLoS One. 2011a:6:e15925. 10.1371/journal.pone.0015925. 21253599 PMC3017084

[msaf251-B36] Kofler R, Pandey RV, Schlötterer C. Popoolation2: identifying differentiation between populations using sequencing of pooled DNA samples (Pool-Seq). Bioinformatics. 2011b:27:3435–3436. 10.1093/bioinformatics/btr589. 22025480 PMC3232374

[msaf251-B37] Kouamo MFM et al Genome-wide transcriptional analysis and functional validation linked a cluster of epsilon glutathione S-transferases with insecticide resistance in the Major malaria vector *Anopheles funestus* across Africa. Genes (Basel). 2021:12, 561. 10.3390/genes12040561. 33924421 PMC8069850

[msaf251-B38] Kouamo MFM et al Allelic variation in a cluster of epsilon glutathione S-transferase genes contributes to DDT and pyrethroid resistance in the major African malaria vector Anopheles funestus. BMC Genomics. 2025:26:452. 10.1186/s12864-025-11637-3. 40335906 PMC12057082

[msaf251-B39] Kwiatkowska RM et al Dissecting the mechanisms responsible for the multiple insecticide resistance phenotype in *Anopheles gambiae* s.s., M form, from Vallée du Kou, Burkina Faso. Gene. 2013:519:98–106. 10.1016/j.gene.2013.01.036. 23380570 PMC3611593

[msaf251-B40] Li H. Aligning sequence reads, clone sequences and assembly contigs with BWA-MEM. 2013. Available from: http://arxiv.org/abs/1303.3997

[msaf251-B41] Love MI, Huber W, Anders S. Moderated estimation of fold change and dispersion for RNA-seq data with DESeq2. Genome Biol. 2014:15:550. 10.1186/s13059-014-0550-8. 25516281 PMC4302049

[msaf251-B42] Lucas ER et al Copy number variants underlie major selective sweeps in insecticide resistance genes in Anopheles arabiensis. PLoS Biol. 2024:22:e3002898. 10.1371/journal.pbio.3002898. 39636817 PMC11620391

[msaf251-B43] Markstein M, Pitsouli C, Villalta C, Celniker SE, Perrimon N. Exploiting position effects and the gypsy retrovirus insulator to engineer precisely expressed transgenes. Nat Genet. 2008:40:476–483. 10.1038/ng.101. 18311141 PMC2330261

[msaf251-B44] Martin M . Cutadapt removes adapter sequences from high-throughput sequencing reads. EMBnet J. 2011:17, 10. Next Generation Sequencing Data Analysis. https://journal.embnet.org/index.php/embnetjournal/article/view/200 10.14806/ej.17.1.200.

[msaf251-B45] Martinez-Torres D et al Molecular characterization of pyrethroid knockdown resistance (kdr) in the major malaria vector Anopheles gambiae s.s. Insect Mol Biol. 1998:7:179–184. 10.1046/j.1365-2583.1998.72062.x. 9535162

[msaf251-B46] Menze BD et al Bionomics and insecticides resistance profiling of malaria vectors at a selected site for experimental hut trials in central Cameroon. Malar J. 2018:17:317. 10.1186/s12936-018-2467-2. 30165863 PMC6117958

[msaf251-B47] Menze BD et al Marked aggravation of pyrethroid resistance in major malaria vectors in Malawi between 2014 and 2021 is partly linked with increased expression of P450 alleles. BMC Infect Dis. 2022:22:660. 10.1186/s12879-022-07596-9. 35907831 PMC9338535

[msaf251-B48] Messenger LA et al A whole transcriptomic approach provides novel insights into the molecular basis of organophosphate and pyrethroid resistance in Anopheles arabiensis from Ethiopia. Insect Biochem Mol Biol. 2021:139:103655. 10.1016/j.ibmb.2021.103655. 34562591 PMC11705372

[msaf251-B49] Morgan JC, Irving H, Okedi LM, Steven A, Wondji CS. Pyrethroid resistance in an Anopheles funestus population from Uganda. PLoS One. 2010:5:e11872. 10.1371/journal.pone.0011872. 20686697 PMC2912372

[msaf251-B50] Mugenzi LMJ et al Cis-regulatory CYP6P9b P450 variants associated with loss of insecticide-treated bed net efficacy against Anopheles funestus. Nat Commun. 2019:10:4652. 10.1038/s41467-019-12686-5. 31604938 PMC6789023

[msaf251-B51] Mugenzi LMJ et al A 6.5-kb intergenic structural variation enhances P450-mediated resistance to pyrethroids in malaria vectors lowering bed net efficacy. Mol Ecol. 2020:29:4395–4411. 10.1111/mec.15645. 32974960

[msaf251-B52] Mugenzi LMJ et al Escalating pyrethroid resistance in two major malaria vectors Anopheles funestus and Anopheles gambiae (s.l.) in Atatam, Southern Ghana. BMC Infect Dis. 2022:22:799. 10.1186/s12879-022-07795-4. 36284278 PMC9597992

[msaf251-B53] Mugenzi LMJ et al Association of a rapidly selected 4.3 kb transposon-containing structural variation with a P450-based resistance to pyrethroids in the African malaria vector Anopheles funestus. PLoS Genet. 2024:20:e1011344. 10.1371/journal.pgen.1011344. 39074161 PMC11309504

[msaf251-B54] Mulamba C et al Widespread pyrethroid and DDT resistance in the major malaria vector Anopheles funestus in East Africa is driven by metabolic resistance mechanisms. PLoS One. 2014:9:e110058. 10.1371/journal.pone.0110058. 25333491 PMC4198208

[msaf251-B55] Nagi SC et al Parallel evolution in mosquito vectors—A duplicated esterase locus is associated with resistance to pirimiphos-methyl in *Anopheles gambiae*. Mol Biol Evol. 2024:41. 10.1093/molbev/msae140. PMC11267716 38985692

[msaf251-B56] Nelson N et al The cellular biology of proton-motive force generation by V-ATPases. Journal of Experimental Biology. 2000:203:89–95. 10.1242/jeb.203.1.89. 10600677

[msaf251-B57] Nguiffo-Nguete D et al Evidence of intensification of pyrethroid resistance in the major malaria vectors in Kinshasa, Democratic Republic of Congo. Sci Rep. 2023:13:14711. 10.1038/s41598-023-41952-2. 37679465 PMC10484898

[msaf251-B58] Odero JO et al Discovery of knock-down resistance in the Major African malaria vector anopheles funestus. Mol Ecol. 2024:33:e17542. 10.1111/mec.17542. 39374937 PMC11537839

[msaf251-B59] Qiagen . 2020. *DNeasy® blood & tissue handbook*. Qiagen. Available from: http://www.bea.ki.se/documents/EN-DNeasy%20handbook.pdf.

[msaf251-B60] Rajaby R et al INSurVeyor: improving insertion calling from short read sequencing data. Nat Commun. 2023:14:3243. 10.1038/s41467-023-38870-2. 37277343 PMC10241795

[msaf251-B61] Reimand J, Kull M, Peterson H, Hansen J, Vilo J. G:Profiler—a web-based toolset for functional profiling of gene lists from large-scale experiments. Nucleic Acids Res. 2007:35:W193–W200. 10.1093/nar/gkm226. 17478515 PMC1933153

[msaf251-B62] Riveron JM et al Directionally selected cytochrome P450 alleles are driving the spread of pyrethroid resistance in the major malaria vector Anopheles funestus. Proc Natl Acad Sci U S A. 2013:110:252–257. 10.1073/pnas.1216705110. 23248325 PMC3538203

[msaf251-B63] Riveron JM et al A single mutation in the GSTe2 gene allows tracking of metabolically based insecticide resistance in a major malaria vector. Genome Biol. 2014:15:R27. 10.1186/gb-2014-15-2-r27. 24565444 PMC4054843

[msaf251-B64] Riveron JM et al Rise of multiple insecticide resistance in anopheles funestus in Malawi: a major concern for malaria vector control. Malar J. 2015:14:344. 10.1186/s12936-015-0877-y. 26370361 PMC4570681

[msaf251-B65] Riveron JM et al Multiple insecticide resistance in the major malaria vector *Anopheles funestus* in southern Ghana: implications for malaria control. Parasit Vectors. 2016:9:504. 10.1186/s13071-016-1787-8. 27628765 PMC5024453

[msaf251-B66] Riveron JM et al Escalation of pyrethroid resistance in the malaria vector *Anopheles funestus* induces a loss of efficacy of piperonyl butoxide-based insecticide-treated nets in Mozambique. J Infect Dis. 2019:220:467–475. 10.1093/infdis/jiz139. 30923819 PMC6603977

[msaf251-B67] Rostant WG, Wedell N, Hosken DJ. Chapter 2—Transposable elements and insecticide resistance. In: Goodwin SF, Friedmann T, Dunlap JC, editors. Advances in genetics. Vol. 78: Academic Press; 2012. p. 169–201. Available from: https://www.sciencedirect.com/science/article/pii/B978012394394100002X 10.1016/B978-0-12-394394-1.00002-X 22980922

[msaf251-B68] Shen R et al Deltamethrin interacts with Culex quinquefasciatus odorant-binding protein: a novel potential resistance mechanism. Parasit Vectors. 2022:15:2. 10.1186/s13071-021-05041-5. 34980219 PMC8725534

[msaf251-B69] Tatchou-Nebangwa NMT et al Two highly selected mutations in the tandemly duplicated CYP6P4a and CYP6P4b genes drive pyrethroid resistance in Anopheles funestus in West Africa. BMC Biol. 2024:22:286. 10.1186/s12915-024-02081-y. 39696366 PMC11657943

[msaf251-B70] Taylor-Wells J, Brooke BD, Bermudez I, Jones AK. The neonicotinoid imidacloprid, and the pyrethroid deltamethrin, are antagonists of the insect Rdl GABA receptor. J Neurochem. 2015:135:705–713. 10.1111/jnc.13290. 26296809

[msaf251-B71] Tazokong HR et al 2024. Nationwide assessment of pyrethroid resistance escalation and investigation of its molecular basis in Anopheles funestus from Cameroon. Available from: https://www.researchsquare.com/article/rs-4710893/v1

[msaf251-B72] Tchouakui M et al Pyrethroid resistance aggravation in Ugandan malaria vectors is reducing bednet efficacy. Pathogens. 2021:10, 415. 10.3390/pathogens10040415. 33915866 PMC8065452

[msaf251-B73] Varela GM, García BA, Stroppa MM. RNA interference of NADPHcytochrome P450 increased deltamethrin susceptibility in a resistant strain of the Chagas disease vector triatoma infestans. Acta Trop. 2024:252:107149. 10.1016/j.actatropica.2024.107149. 38360259

[msaf251-B74] Wamba ANR et al The cytochrome P450 CYP325A is a major driver of pyrethroid resistance in the major malaria vector Anopheles funestus in Central Africa. Insect Biochem Mol Biol. 2021:138:103647. 10.1016/j.ibmb.2021.103647. 34530119

[msaf251-B75] Wang X, Chen X, Zhou T, Dai W, Zhang C. NADPH-cytochrome P450 reductase mediates resistance to neonicotinoid insecticides in *Bradysia odoriphaga*. Pestic Biochem Physiol. 2025:211:106406. 10.1016/j.pestbp.2025.106406. 40350226

[msaf251-B76] Wangrawa DW, Odero JO, Baldini F, Okumu F, Badolo A. Distribution and insecticide resistance profile of the major malaria vector *Anopheles funestus* group across the African continent. Med Vet Entomol. 2024:38:119–137. 10.1111/mve.12706. 38303659

[msaf251-B77] Weedall GD et al A cytochrome P450 allele confers pyrethroid resistance on a major African malaria vector, reducing insecticide-treated bednet efficacy. Sci Transl Med. 2019:11:eaat7386. Available from 10.1126/scitranslmed.aat7386. 30894503

[msaf251-B78] Weedall GD et al An Africa-wide genomic evolution of insecticide resistance in the malaria vector Anopheles funestus involves selective sweeps, copy number variations, gene conversion and transposons. PLoS Genet. 2020:16:e1008822. 10.1371/journal.pgen.1008822. 32497040 PMC7297382

[msaf251-B80] WHO . Test procedures for insecticide resistance monitoring in malaria vector mosquitoes. 2nd ed World Health Organization; 2016. Available from: https://apps.who.int/iris/handle/10665/250677

[msaf251-B79] WHO . 2024. *World malaria report*. Available from: https://www.who.int/teams/global-malaria-programme/reports/world-malaria-report-2024.

[msaf251-B81] Wickham H, Wickham H. Data analysis. Springer; 2016.

[msaf251-B82] Wondji CS et al Mapping a quantitative trait locus (QTL) conferring pyrethroid resistance in the African malaria vector *Anopheles funestus*. BMC Genomics. 2007:8:34. 10.1186/1471-2164-8-34. 17261170 PMC1790900

[msaf251-B83] Wondji CS et al Two duplicated P450 genes are associated with pyrethroid resistance in anopheles funestus, a major malaria vector. Genome Res. 2009:19:452–459. 10.1101/gr.087916.108. 19196725 PMC2661802

[msaf251-B84] Wondji CS, Hearn J, Irving H, Wondji MJ, Weedall G. RNAseq-based gene expression profiling of the *Anopheles funestus* pyrethroid-resistant strain FUMOZ highlights the predominant role of the duplicated CYP6P9a/b cytochrome P450s. G3 (Bethesda). 2022:12:jkab352. 10.1093/g3journal/jkab352. PMC8727960 34718535

[msaf251-B85] Xu H et al Chemosensory proteins confer adaptation to the ryanoid anthranilic diamide insecticide cyantraniliprole in *Aphis gossypii* glover. Pestic Biochem Physiol. 2022:184:105076. 10.1016/j.pestbp.2022.105076. 35715031

[msaf251-B86] Zeng J, Kang W-N, Jin L, Anjum AA, Li G-Q. Knockdown of vacuolar ATPase subunit G gene affects larval survival and impaired pupation and adult emergence in *Henosepilachna vigintioctopunctata*. Insects. 2021:12, 935. 10.3390/insects12100935. 34680704 PMC8538789

[msaf251-B87] Zhuang HM et al Identification and expression of caspase-1 gene under heat stress in insecticide-susceptible and -resistant *Plutella xylostella* (Lepidoptera: Plutellidae). Mol Biol Rep. 2011:38:2529–2539. 10.1007/s11033-010-0391-9. 21086181

