# Supplementary material for: Genomic Drivers of Pyrethroid Resistance Escalation in the Malaria Vector Anopheles funestus Across Africa
Source: Mol Biol Evol. 2025 Oct 24;42(10):msaf251. doi: 10.1093/molbev/msaf251 (PMC12550562; doi:10.1093/molbev/msaf251)
Supplement: msaf251_Supplementary_Data [file msaf251_supplementary_data.zip › Suppementary_Materials.pdf]

Supplementary Figures

Supplementary figure 1: Principal component analysis of gene expression profiling. 2
Supplementary figure 2: Volcano plots showing the expression profile of *An. funestus* for 2021 cohort against FANG 2023 in each country. 3
Supplementary figure 3: Volcano plots showing the expression profile of *An. funestus* for 2014 cohort against FANG 2014 in each country. 4
Supplementary figure 4: Trend of top 20 known detoxification genes previously linked with insecticide resistance in 2021 cohort and 2014 cohort relative to the respective batch of FANG. 5
Supplementary figure 5: Gene ontology (GO) of the commonly overexpressed genes in *An. funestus* across Africa (A) and dose response in Malawian mosquitoes (B). 6
Supplementary figure 6: Population structure and correlation plots of *An. funestus* across Africa...... 8
Supplementary figure 7: Genome-wide patterns of genetic diversity and Tajima's D across Anopheles funestus populations in Africa. 8
Supplementary figure 8: Temporal evolution of key variants in *An. funestus* across Africa. 9
Supplementary figure 9: IGV screenshot of the alignment around the CYP6 region showing a pattern characteristic of a transposon insertion of 4.3kb located in the intergenic region of *CYP6P9b* and *CYP6P5*. 10
Supplementary figure 10: IGV screenshot of the alignment around the CYP9 region showing a pattern characteristic of a transposon insertion of unknown size located upstream *CYP9K1* gene. 10
Supplementary figure 11: Schematic representation of the duplication events around the CYP6 locus on chromosome 2R and X in *An. funestus* across Africa. 11
Supplementary figure 12: IGV screenshot of the alignment around the CYP6 region showing DUP4, DUP6 and DUP7 spanning *CYP6AA1*, *CYP6AA2*, 2x carboxylesterases and P450 *AFUN008357*. .11
Supplementary figure 13: IGV screenshot of the alignment around the CYP6 region showing DUP2 spanning 2x carboxylesterases. 12
Supplementary figure 14: IGV screenshot of the alignment around the CYP9 region showing *CPR* duplication spanning the upstream promoter region. 12
Supplementary figure 15: H_{12} signal of recent selection spanning the X chromosome in *An. funestus* across Africa. 13
Supplementary figure 16: Temporal F_{ST} signal of genetic differentiation spanning the X chromosome in An. funestus PoolSeq data across Africa. 13
Supplementary figure 17: F_{ST} signal of genetic differentiation spanning the X chromosome in An. funestus RNAseq data across Africa. 14
Supplementary figure 18: Temporal F_{ST} signal of genetic differentiation spanning the X chromosome in An. funestus RNAseq data across Africa. 15

Supplementary figure 19: F_{ST} signal of genetic differentiation spanning the X chromosome in *An. funestus* RNAseq data across Africa. 16
Supplementary figure 20: Diplotype clustering at *CPR* and *CYP9K1* loci in An. funestus across Africa. 16
Supplementary figure 21: Frequency of *CYP9K1* duplication in An. funestus across Africa. 17
Supplementary figure 22: CNV HMM plot showing copy number state in *An. funestus* population from Ghana. The vertical color bar represents discrete copy number states used in CNV (Copy Number Variation) heatmaps. 18
Supplementary figure 23: Confirmation of *CYP6P9a* and *CPR* genes expression in 6P9a-N70-CPR and 6P9a-70I-*CPR* transgenic *Drosophila*, and absence of expression in control flies, trough semiquantitative PCR. 19
Supplementary figure 24: Study sites map. 19

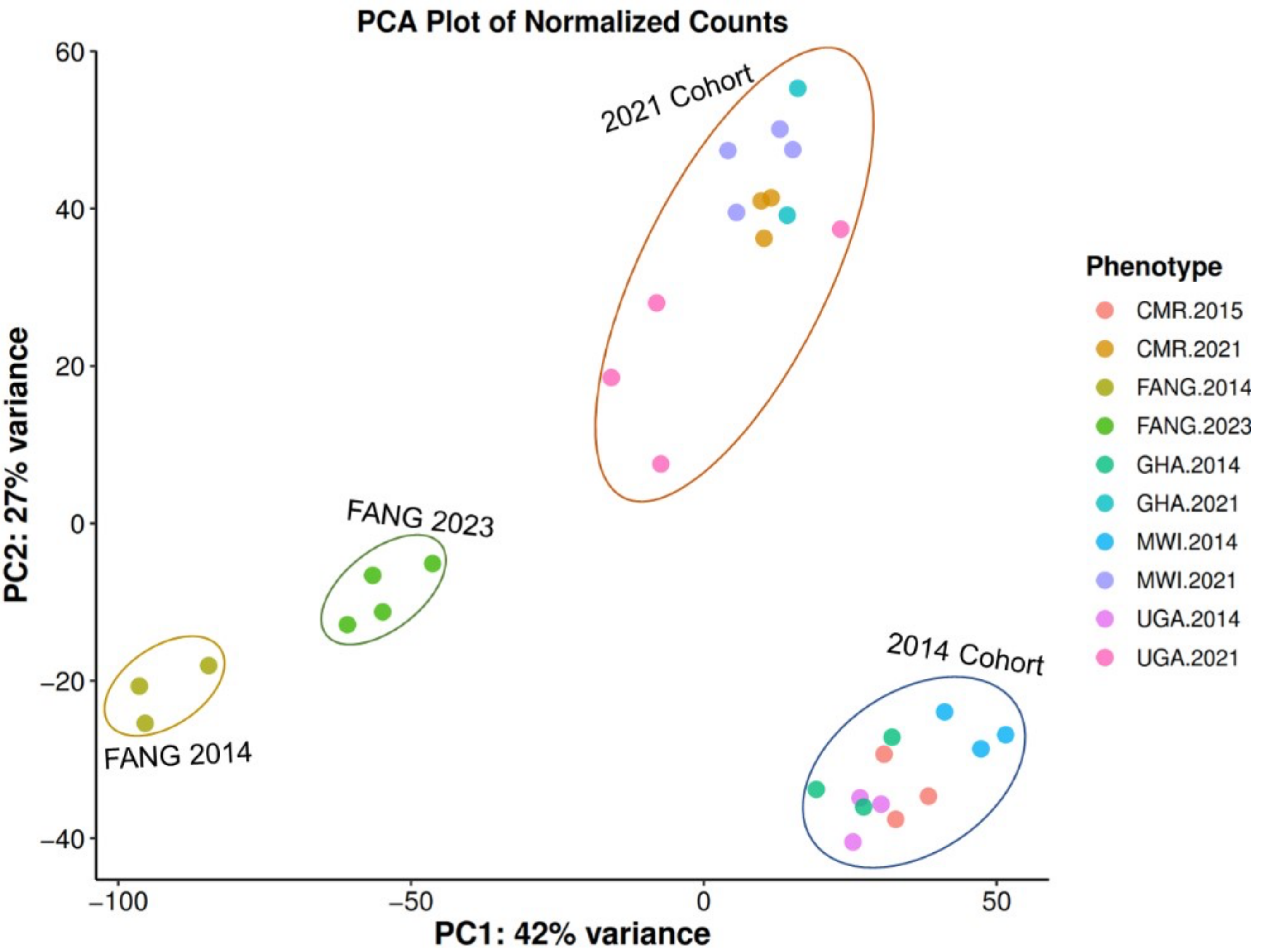

Supplementary figure 1: Principal component analysis of gene expression profiling.

The PC1 and PC2 explained in total 69% variance with 27% and 42% variance explained by PC1 and PC2, respectively.

Four distinct clusters were observed: the two batches of FANG formed two clusters separated by PC1 but were closed indicating shared similar genetic background but different due to noise introduced by sequencing bias, the two other clusters were clearly distant from each other by PC2 suggesting mainly difference in resistance phenotype between 2014 cohort and 2021 cohort.

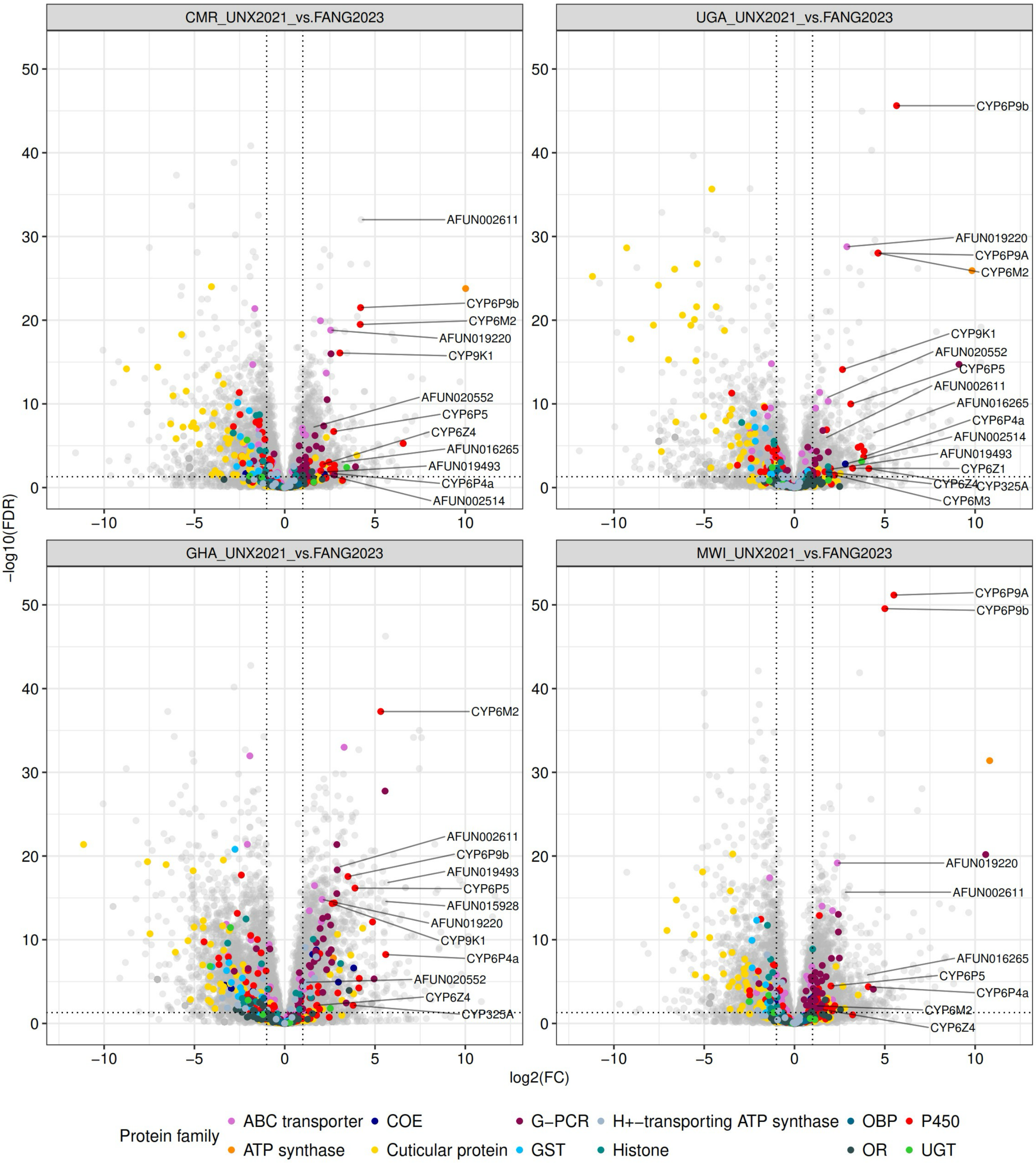

Supplementary figure 2: Volcano plots showing the expression profile of *An. funestus* for 2021 cohort against FANG 2023 in each country.

The genes highlighted in colour belong to known protein families and some putative genes to be involved in insecticide resistance. Only top genes belonging to category depicted in protein family caption are annotated. Genes above the horizontal dotted line passed our threshold for significance (FDR <0.05). Genes on the right of the dotted vertical line (log2FC >1) were up-regulated in the 2021 cohort than in the susceptible FANG 2023, whereas genes on the left of the vertical dotted line (log2FC <1) were down-regulated in 2021 than in susceptible FANG 2023. UNX: Unexposed.

ABC=ATP-binding cassette; COE: Carboxylesterases, GST: Glutathione S-Transferases, UGT: UDP-glucuronosyltransferases; P450: Cytochrome P450s; OBP: Odorant-binding protein, OR: Odorant receptor, G-PCR: G-Protein Coupled Receptors. CMR, MWI, UGA and GHA represent Cameroon, Malawi, Uganda and Ghana, respectively.

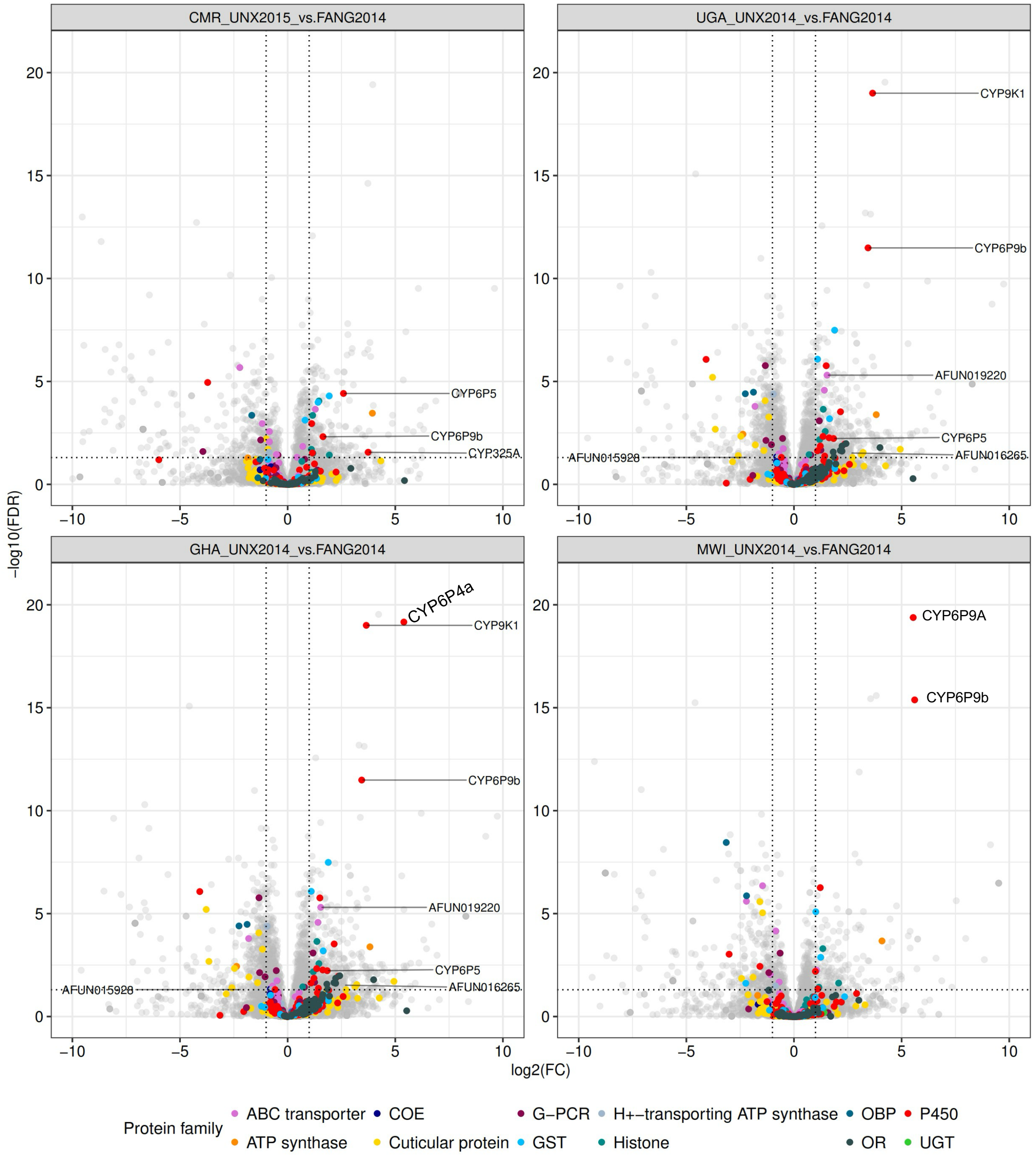

Supplementary figure 3: Volcano plots showing the expression profile of *An. funestus* for 2014 cohort against FANG 2014 in each country.

The genes highlighted in colour belong to known protein families and some putative genes to be involved in insecticide resistance. Other gene families are depicted in gray. Top genes previously linked to insecticide resistance belonging to category depicted in protein family caption are annotated. Genes above the horizontal dotted line passed our threshold for significance (FDR <0.05). Genes on the right of the dotted vertical line (log2FC ≥1) were up-regulated in the 2014

cohort than in the susceptible FANG 2014, whereas genes on the left of the vertical dotted line (log2FC ≤1) were down-regulated in 2014 than in susceptible FANG 2014. UNX: Unexposed. ABC=ATP-binding cassette; COE: Carboxylesterases, GST: Glutathione S-Transferases, UGT:

UDP-glucuronosyltransferases; P450: Cytochrome P450s; OBP: Odorant-binding protein, OR: Odorant receptor, G-PCR: G-Protein Coupled Receptors. CMR, MWI, UGA and GHA represent Cameroon, Malawi, Uganda and Ghana, respectively.

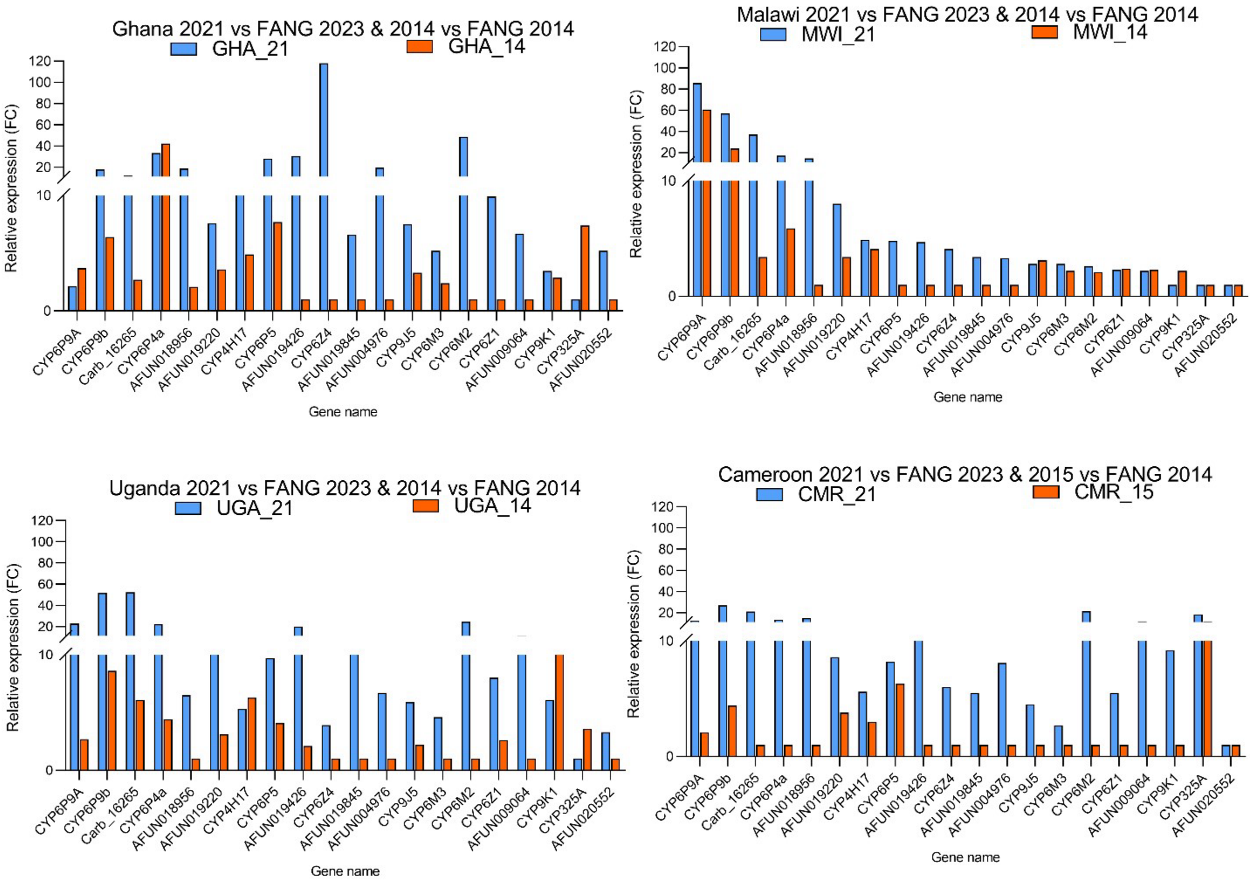

Supplementary figure 4: Trend of top 20 known detoxification genes previously linked with insecticide resistance in 2021 cohort and 2014 cohort relative to the respective batch of FANG.

The blue bar plot represents the pairwise comparison between 2021 vs FANG 2023 while the orange bar plot represents the pairwise comparison between 2014 cohort vs FANG 2014. The cutoff of differential expression was FDR <0.05 and fold change ≥2. FC: fold change. The break on the yaxis allows better visualization, the bottom indicates fold change ranging from 0 to 10 and the top indicates fold change ranging from 11 to 120.

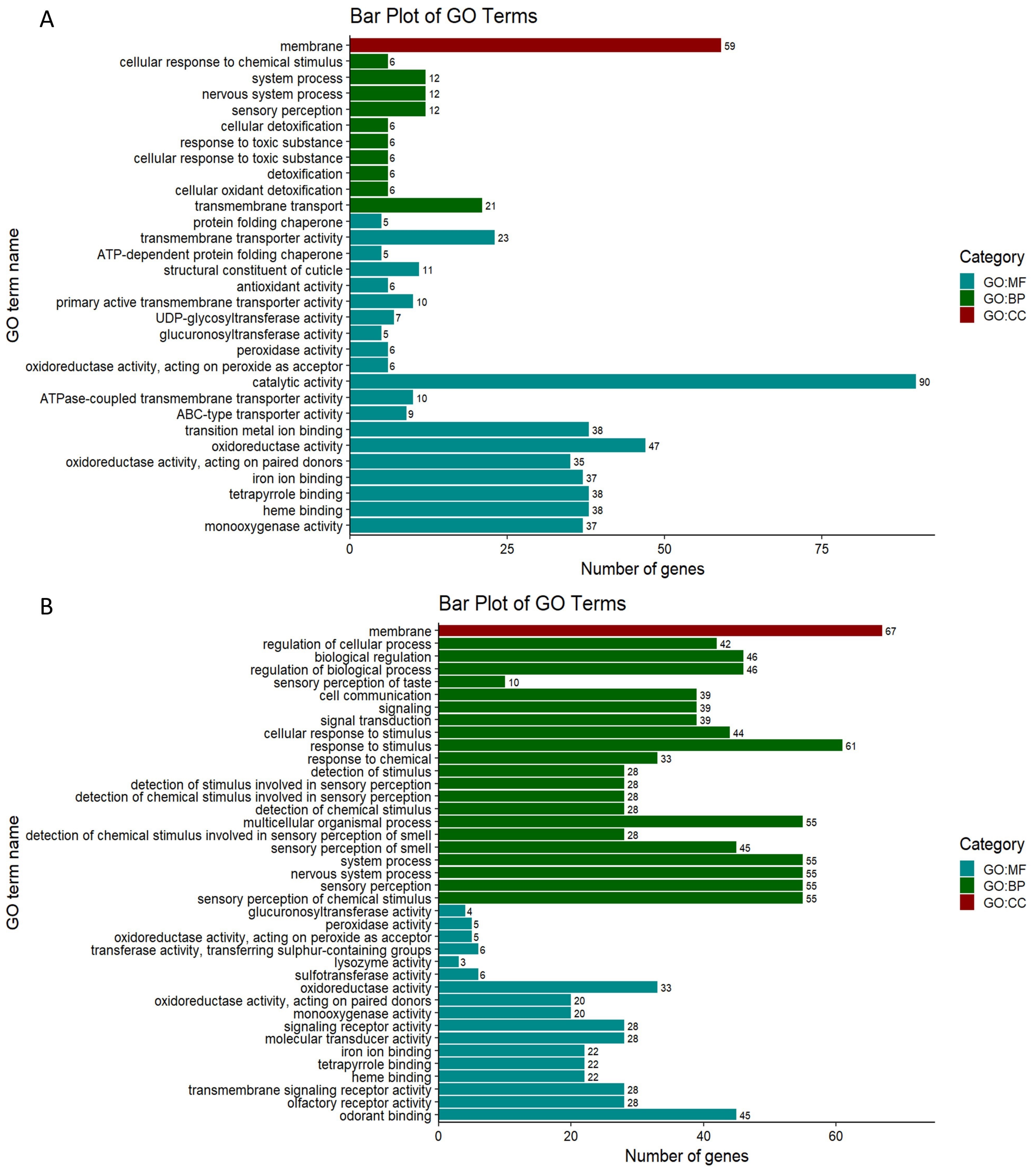

Supplementary figure 5: Gene ontology (GO) of the commonly overexpressed genes in *An. funestus* across Africa (A) and dose response in Malawian mosquitoes (B).

MF, BP and CC stand for Molecular function, biological process and Cellular component, respectively.

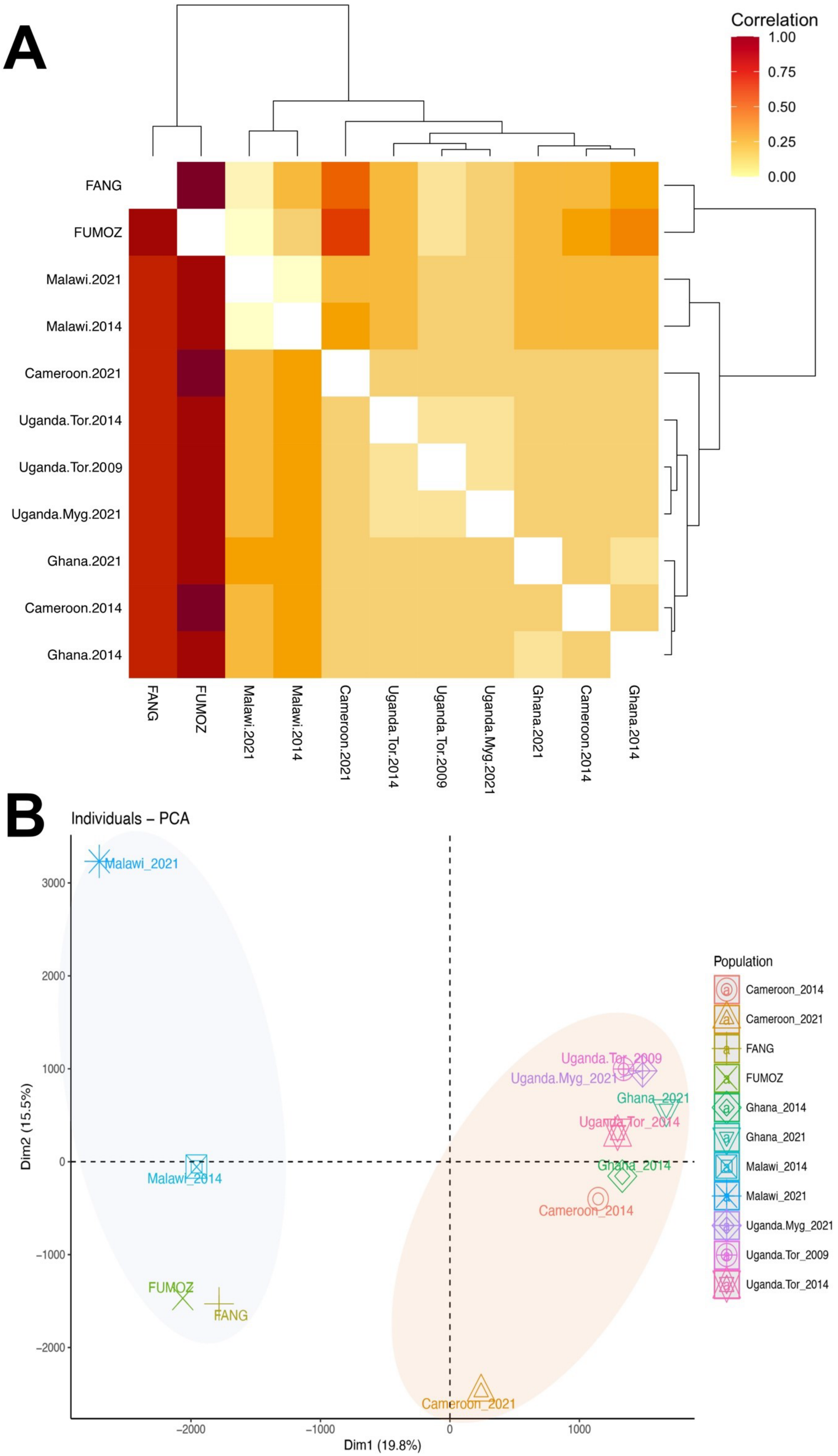

A
Correlation
1.00
0.75
0.50
0.25
0.00
FANG
FUMOZ
Malawi.2021
Malawi.2014
Cameroon.2021
Uganda.Tor.2014
Uganda.Tor.2009
Uganda.Myg.2021
Ghana.2021
Cameroon.2014
Ghana.2014
B
Individuals - PCA
Dim1 (19.8%)
Dim2 (15.5%)
Population
Cameroon_2014
Cameroon_2021
FANG
FUMOZ
Ghana_2014
Ghana_2021
Malawi_2014
Malawi_2021
Uganda.Myg_2021
Uganda.Tor_2009
Uganda.Tor_2014

Supplementary figure 6: Population structure and correlation plots of *An. funestus* across Africa.

(A) Correlation plot of *An. funestus* collected across Africa between 2009 and 2021 (with 2014 samples from Weedall et al paper (Weedall et al., 2020) included for temporal comparison). The correlation plot was computed using genome-wide F_{ST} values from different comparisons between populations across Africa. The variation in color is depicted using a soft yellow-yellow-dark red colour scale, where soft yellow indicates low correlation (<0.25), yellow represents moderate correlation (0.25-0.75), and red dark denotes high correlation between populations (>0.75). (B) PCA supplemented with laboratory strains FANG and FUMOZ which originated from Southern Africa.

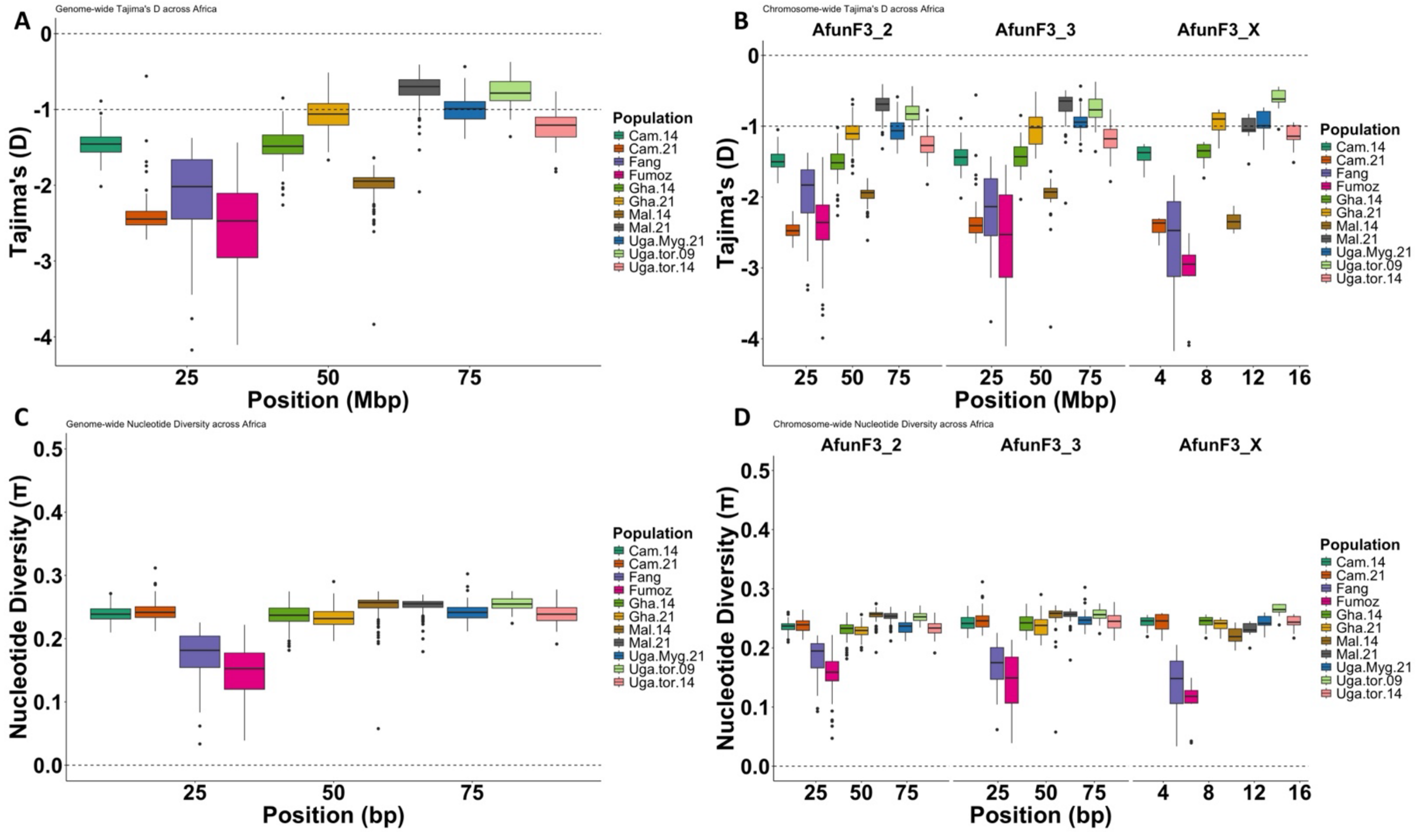

Supplementary figure 7: Genome-wide patterns of genetic diversity and Tajima's D across *An. funestus* populations in Africa.

The plots present genome-wide analyses of Tajima's D (A–B) and nucleotide diversity (π) (C–D) across An. funestus mosquitoes sampled from Cameroon (2014, 2021), Ghana (2014, 2021), Malawi (2014, 2021), Uganda (Tororo 2009, Tororo 2014, Mayuge 2021), as well as the FUMOZ laboratory strain. Tajima's D values (A, B) were calculated in non-overlapping windows of 50 kb across chromosomes 2, 3, and X, with values ranging from 0 to -4. Nucleotide diversity (π) (C, D) was estimated in the same windows, ranging from 0 to 0.3, with lower values reflecting reduced genetic variation. In the plots, the y-axes represent Tajima's D or nucleotide diversity, while the x-axes show genomic positions in megabase pairs (Mbp).

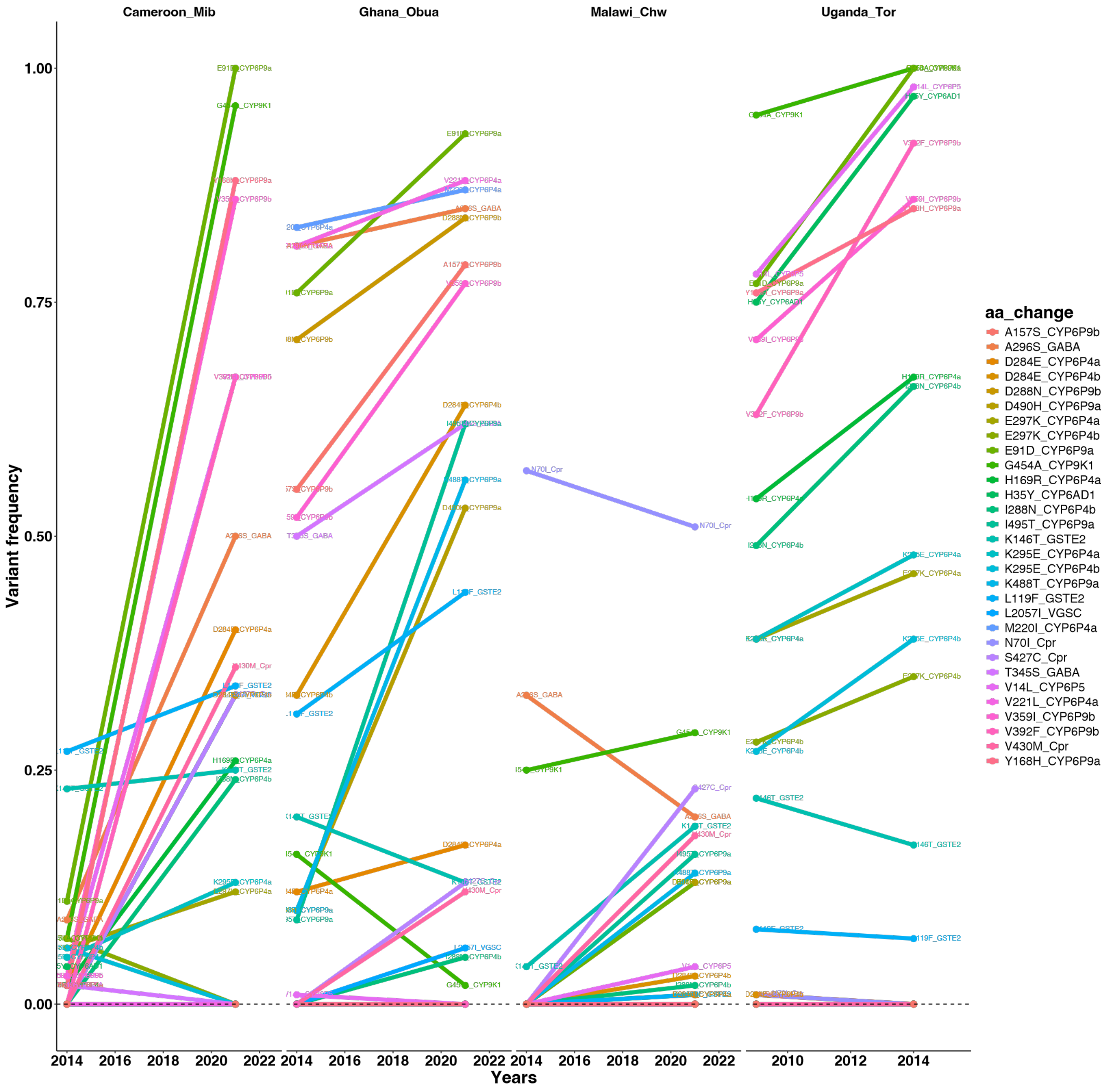

Supplementary figure 8: Temporal evolution of key variants in *An. funestus* across Africa.

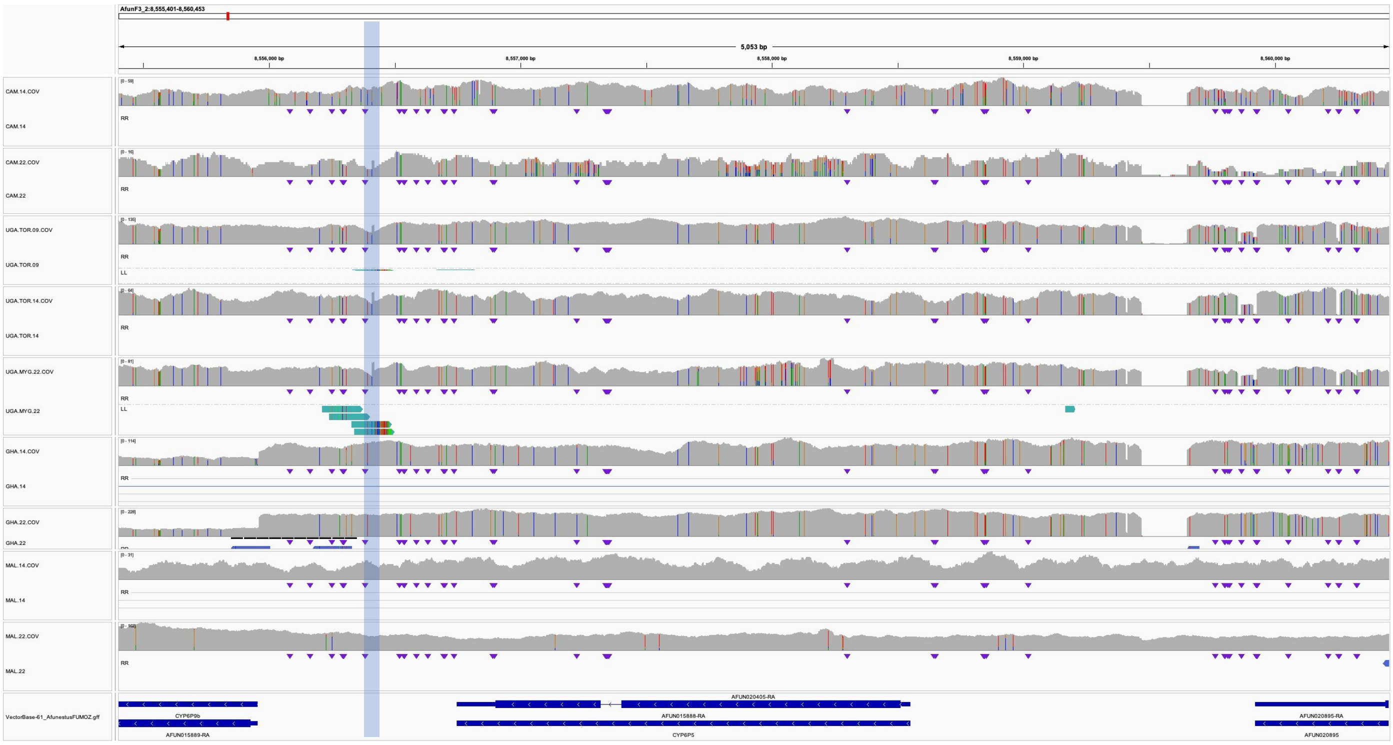

Supplementary figure 9: IGV screenshot of the alignment around the CYP6 region showing a pattern characteristic of a transposon insertion of 4.3kb located in the intergenic region of *CYP6P9b* and *CYP6P5*. The blue box indicates the transposon insertion for each sample.

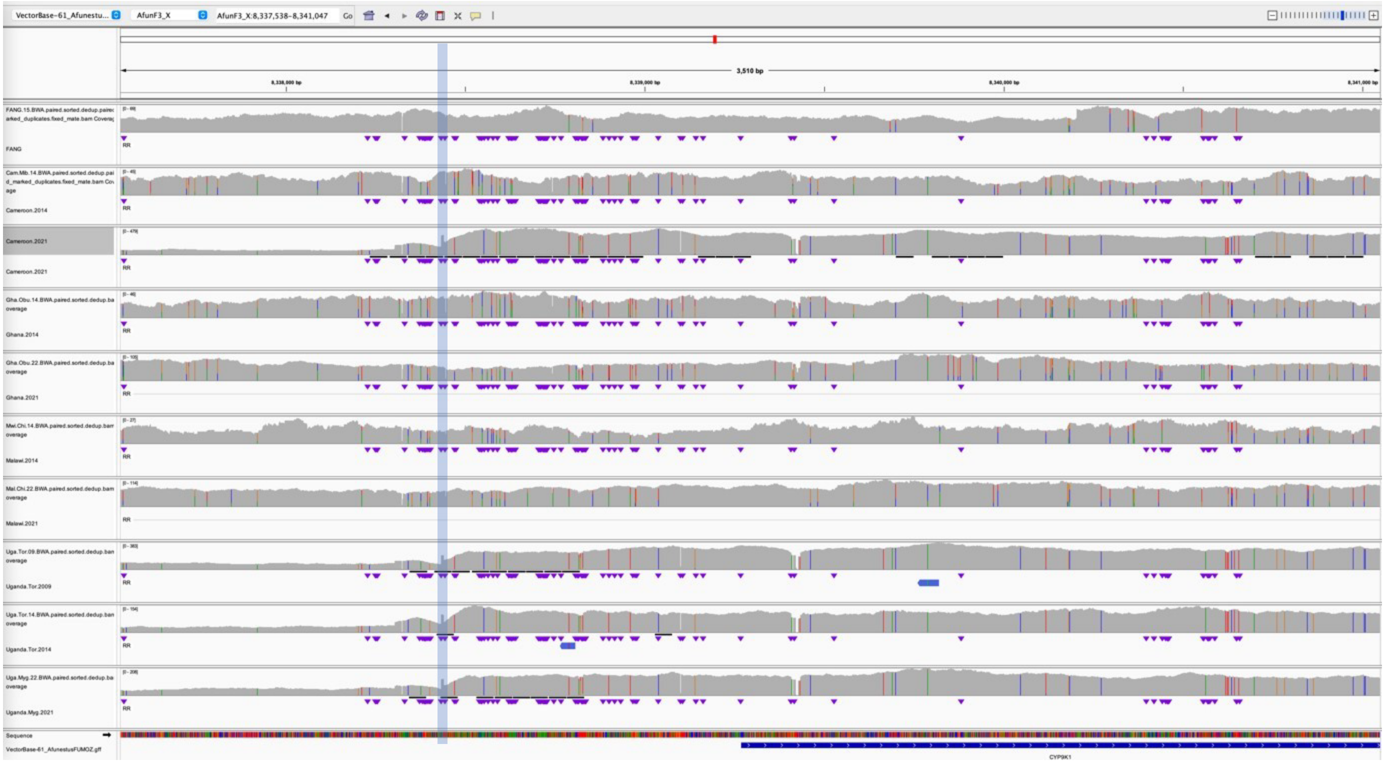

Supplementary figure 10: IGV screenshot of the alignment around the CYP9 region showing a pattern characteristic of a transposon insertion of unknown size located upstream *CYP9K1* gene.

The blue box indicates the transposon insertion for each sample.

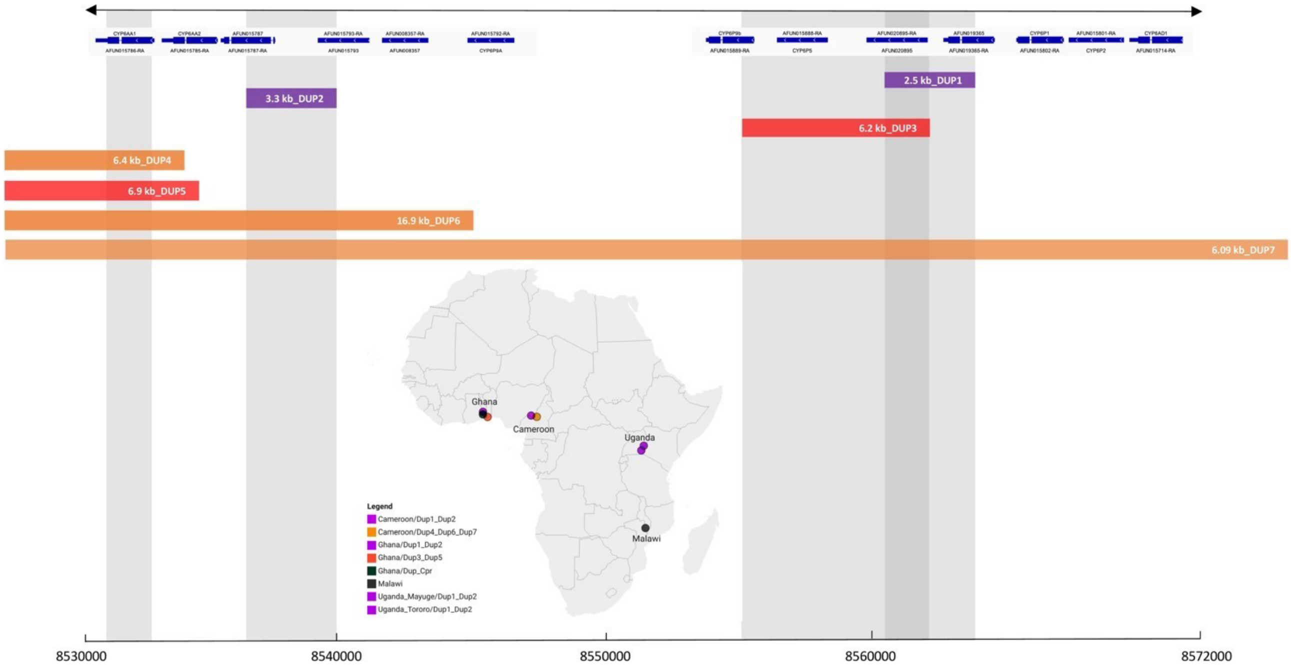

Supplementary figure 11: Schematic representation of the duplication events around the CYP6 locus on chromosome 2R and X in *An. funestus* across Africa.

The duplications are highlighted with horizontal bars and different colours on the Figure.

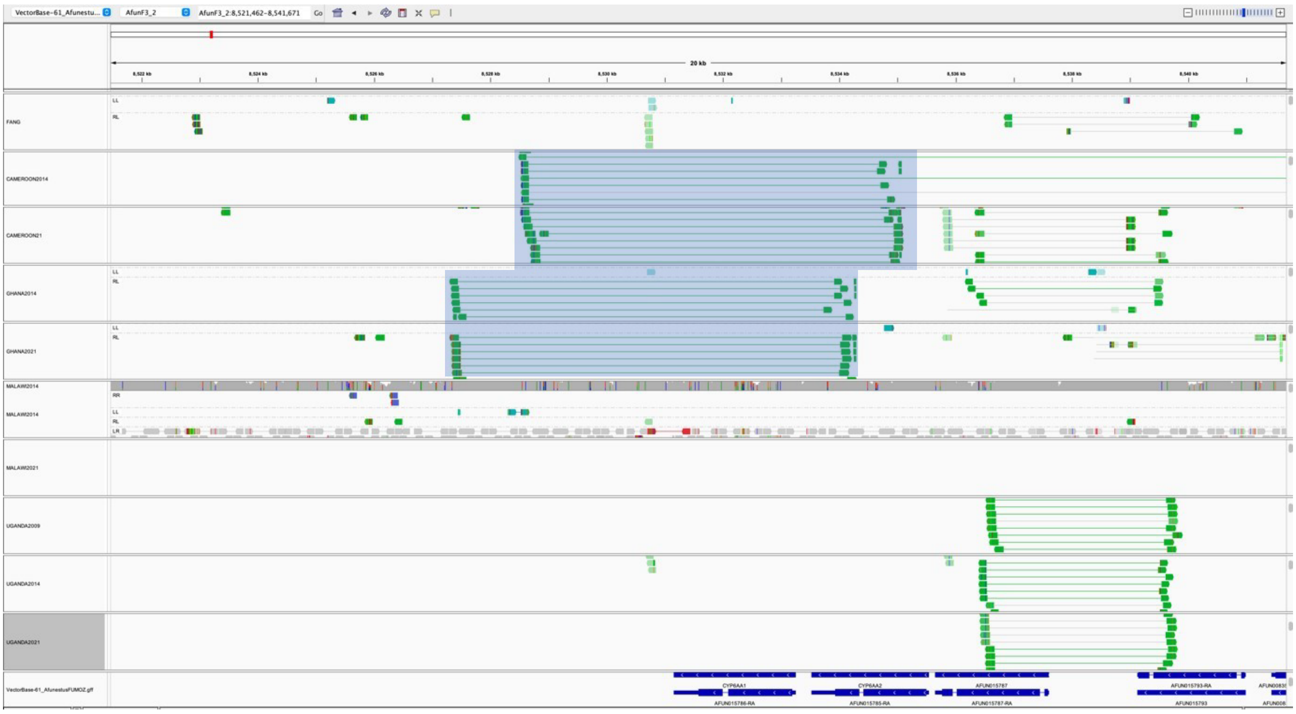

Supplementary figure 12: IGV screenshot of the alignment around the CYP6 region showing DUP4, DUP6 and DUP7 spanning *CYP6AA1*, *CYP6AA2*, 2x carboxylesterases and P450 *AFUN008357*.

The blue box indicates the extend of the duplications.

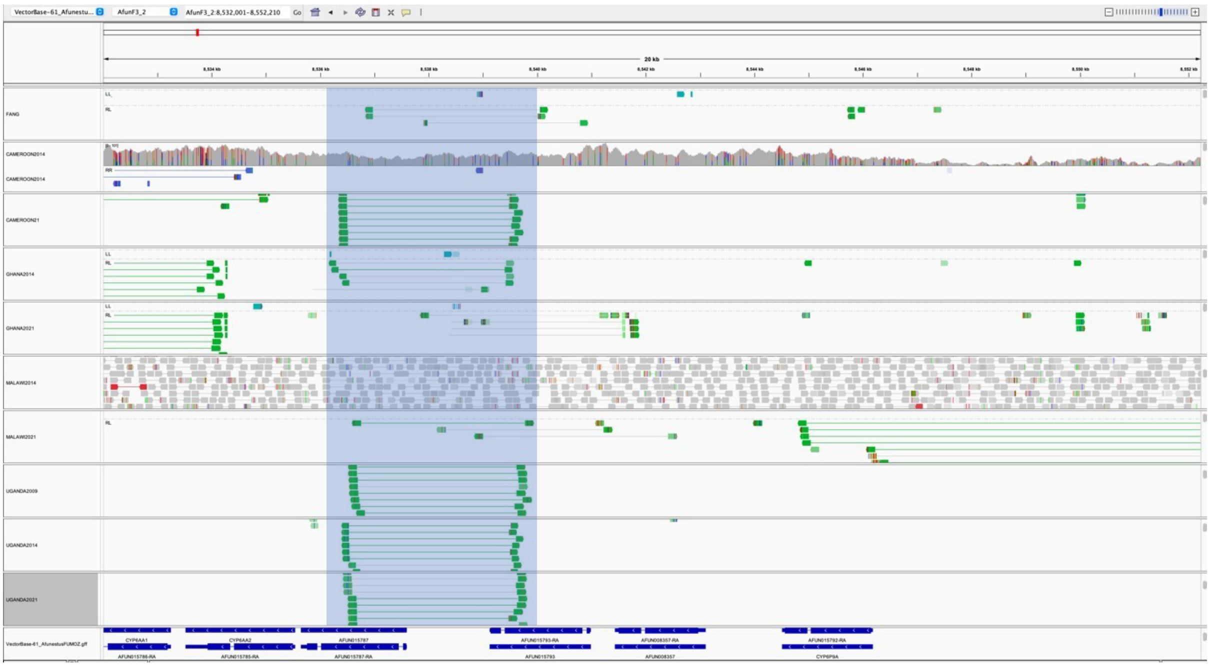

Supplementary figure 13: IGV screenshot of the alignment around the CYP6 region showing DUP2 spanning 2x carboxylesterases.

The blue box indicates the extend of the duplication.

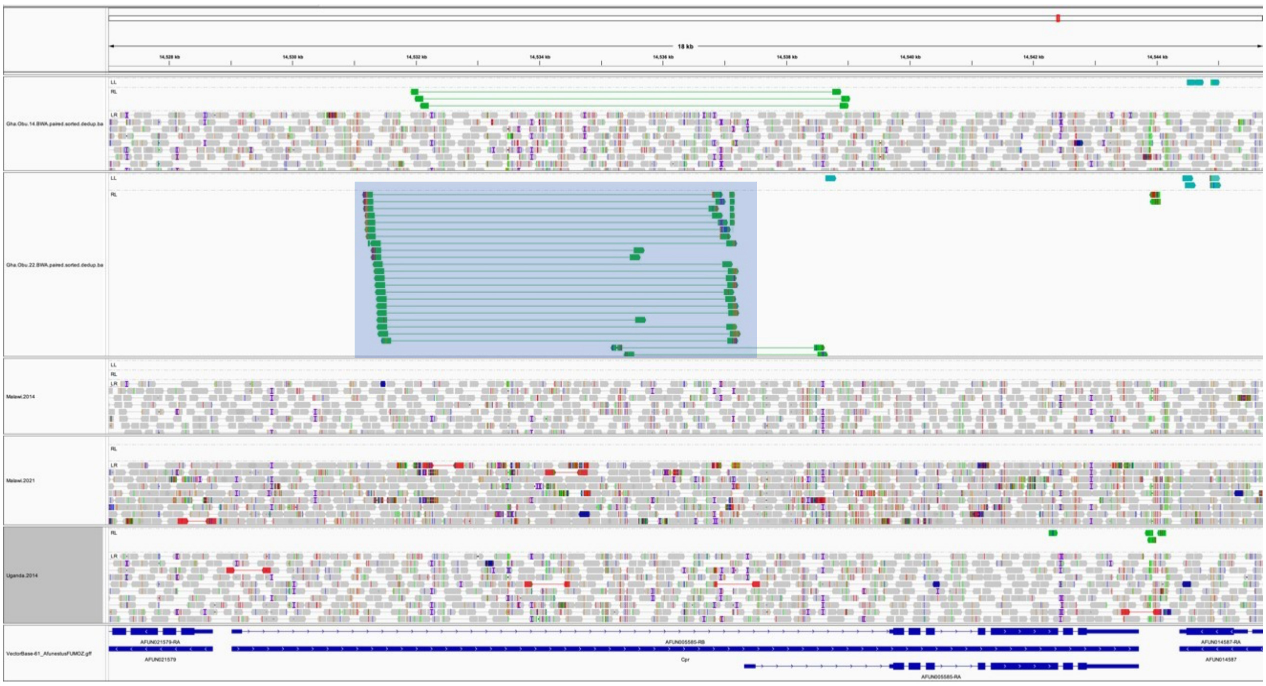

Supplementary figure 14: IGV screenshot of the alignment around the CYP9 region showing *CPR* duplication spanning the upstream promoter region.

The blue box indicates the extend of the *CPR* duplication.

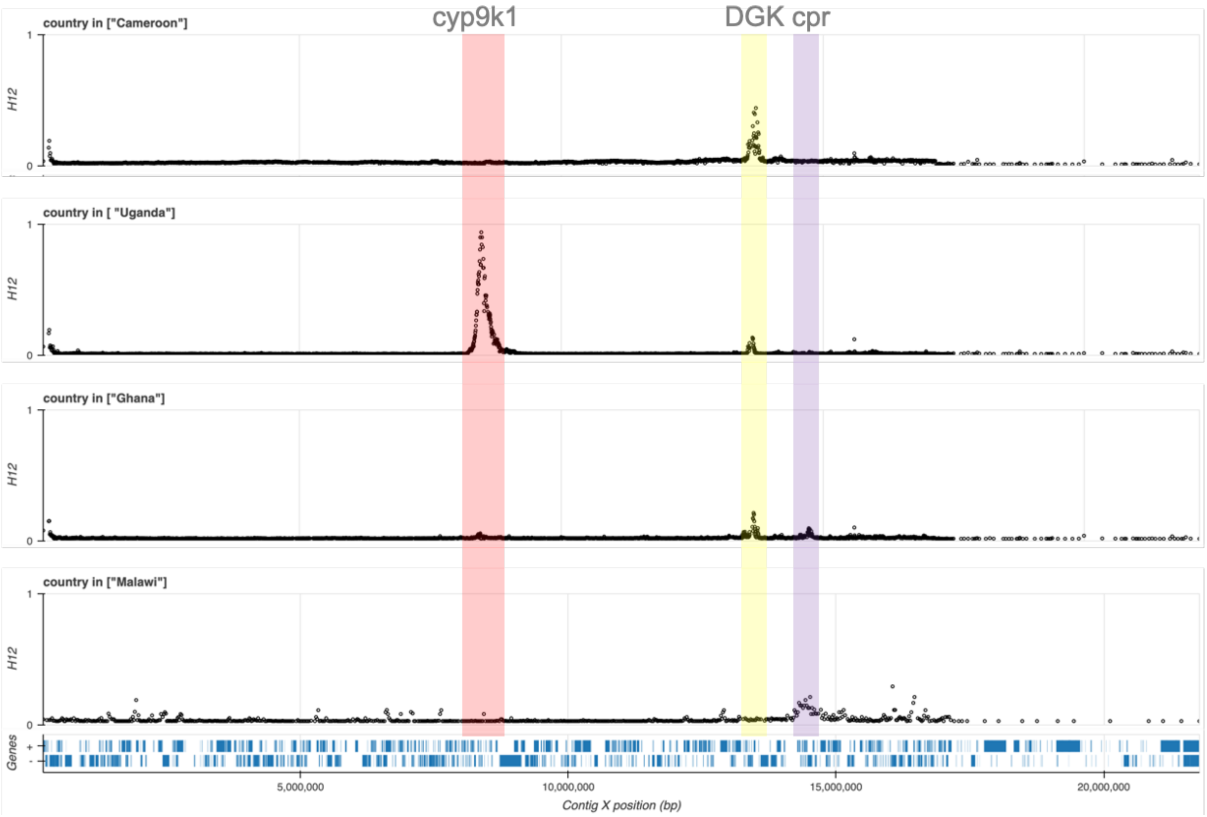

Supplementary figure 15: H_{12} signal of recent selection spanning the X chromosome in *An. funestus* across Africa.

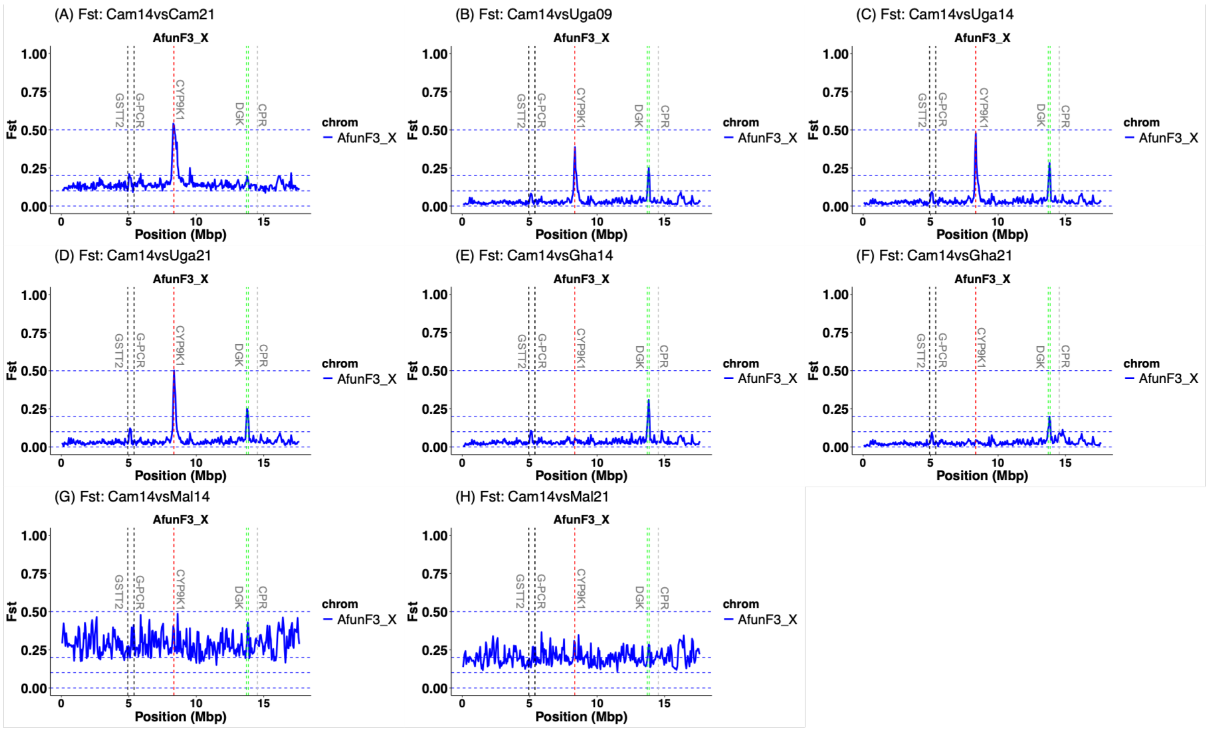

Supplementary figure 16: Temporal F_{ST} signal of genetic differentiation spanning the X chromosome in *An. funestus* PoolSeq data across Africa.

GSTT2 is a member of the glutathione S-transferase T class, involved in detoxification processes. *G-PCR* stands for G protein-coupled receptor, which has been associated with modulating P450

enzyme activity. The *CYP9K1* gene represents a major cytochrome P450 locus linked to insecticide resistance across Africa. *DGK* refers to the diacylglycerol kinase gene, which plays a role in lipid signaling pathways, while *Cpr* denotes the cytochrome P450 reductase gene, an essential partner in P450-mediating metabolic processes.

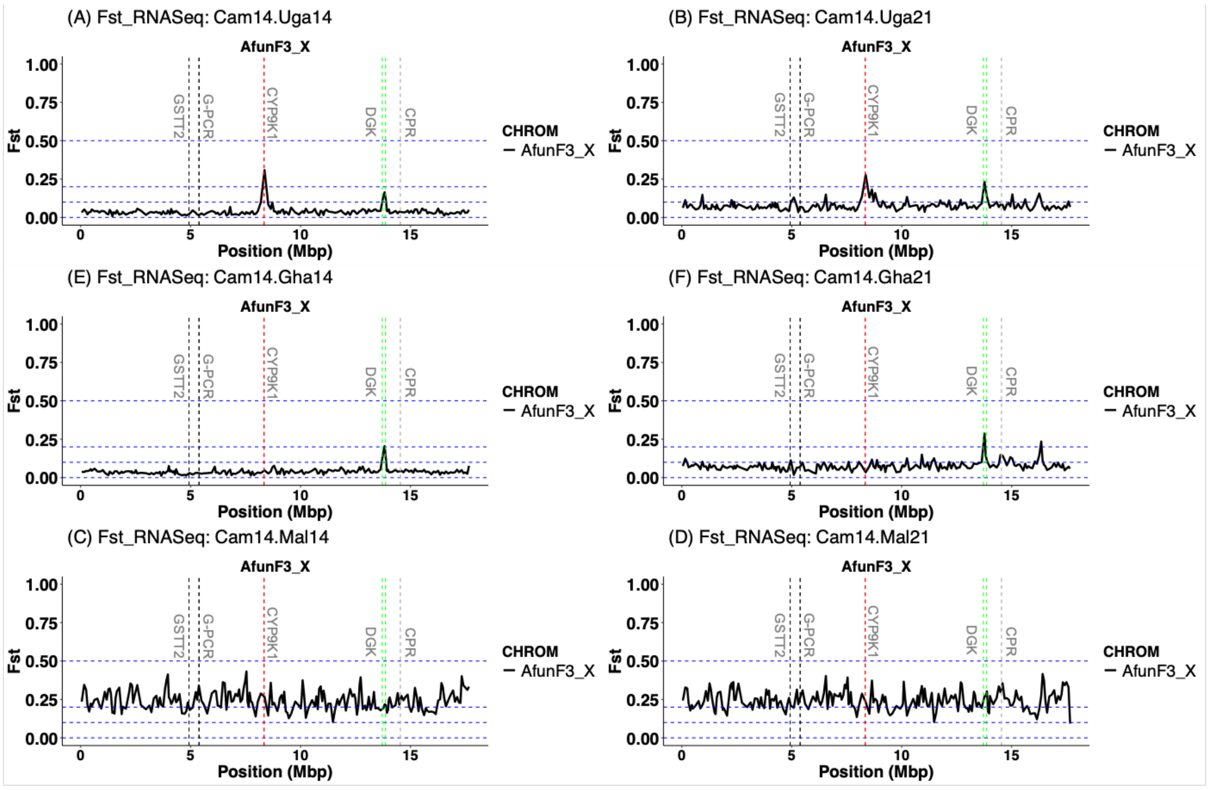

Supplementary figure 17: F_{ST} signal of genetic differentiation spanning the X chromosome in *An. funestus* RNAseq data across Africa.

GSTT2 is a member of the glutathione S-transferase T class, involved in detoxification processes. *G-PCR* stands for G protein-coupled receptor, which has been associated with modulating P450 enzyme activity. The *CYP9K1* gene represents a major cytochrome P450 locus linked to insecticide resistance across Africa. *DGK* refers to the diacylglycerol kinase gene, which plays a role in lipid signaling pathways, while *Cpr* denotes the cytochrome P450 reductase gene, an essential partner in P450-mediated metabolic processes.

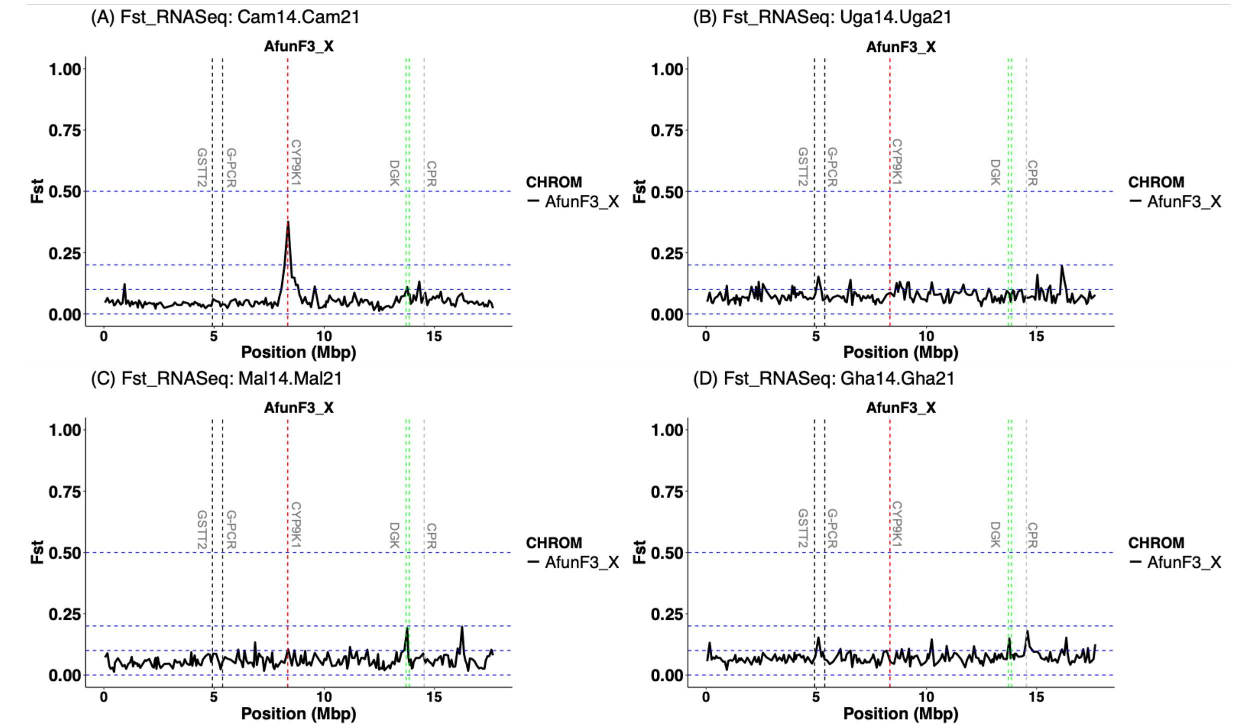

Supplementary figure 18: Temporal F_{ST} signal of genetic differentiation spanning the X chromosome in *An. funestus* RNAseq data across Africa.

GSTT2 is a member of the glutathione S-transferase T class, involved in detoxification processes. *G-PCR* stands for G protein-coupled receptor, which has been associated with modulating P450 enzyme activity. The *CYP9K1* gene represents a major cytochrome P450 locus linked to insecticide resistance across Africa. *DGK* refers to the diacylglycerol kinase gene, which plays a role in lipid signaling pathways, while *Cpr* denotes the cytochrome P450 reductase gene, an essential partner in P450-mediated metabolic processes.

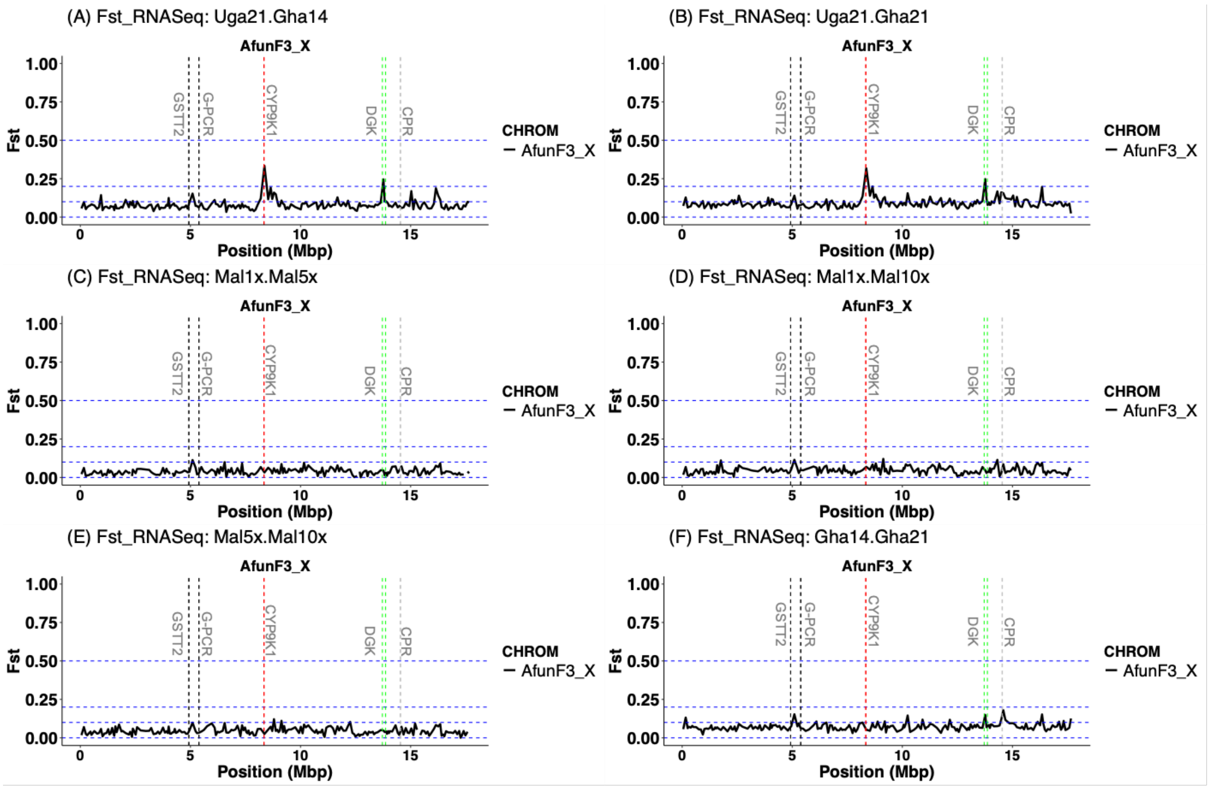

Supplementary figure 19: F_{ST} signal of genetic differentiation spanning the X chromosome in *An. funestus* RNAseq data across Africa.

GSTT2 is a member of the glutathione S-transferase T class, involved in detoxification processes. *G-PCR* stands for G protein-coupled receptor, which has been associated with modulating P450 enzyme activity. The *CYP9K1* gene represents a major cytochrome P450 locus linked to insecticide resistance across Africa. *DGK* refers to the diacylglycerol kinase gene, which plays a role in lipid signaling pathways, while *Cpr* denotes the cytochrome P450 reductase gene, an essential partner in P450-mediated metabolic processes.

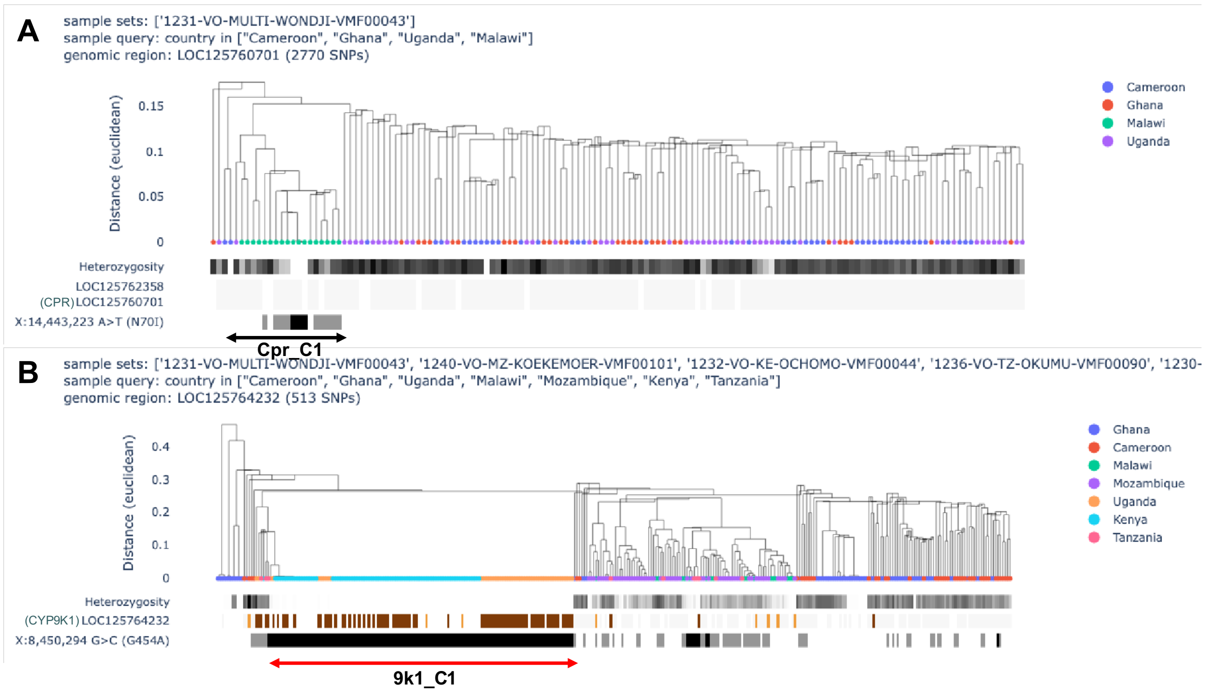

Supplementary figure 20: Diplotype clustering at *CPR* and *CYP9K1* loci in *An. funestus* across Africa.

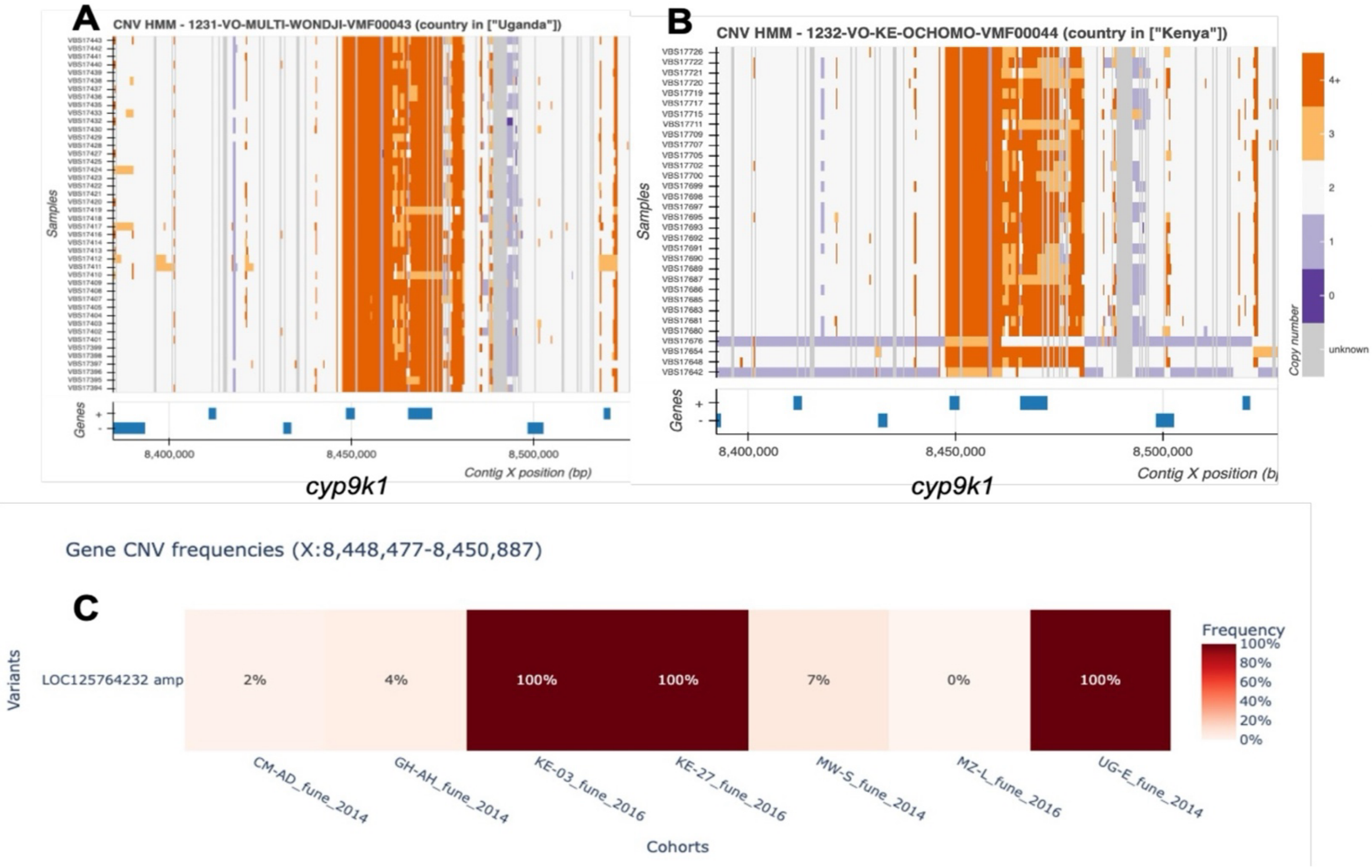

Supplementary figure 21: Frequency of *CYP9K1* duplication in *An. funestus* across Africa.

A. CNV HMM model showing samples affected by *CYP9K1* CNV in Uganda population. B. CNV HMM model showing samples affected by *CYP9K1* CNV in Kenya population and C. heatmap showing *CYP9K1* Copy number allele frequency in *An. funestus* populations across Africa. The vertical color bar represents discrete copy number states used in CNV (Copy Number Variation) heatmaps. Each color corresponds to a specific copy number value for each sample: dark orange indicates high-level amplification (copy number ≥ 4), medium orange represents moderate amplification (copy number = 3), white denotes normal diploid copy number (copy number = 2), light purple reflects single-copy loss (copy number = 1), dark purple signifies homozygous deletion (copy number = 0), and gray indicates unknown or missing copy number data.

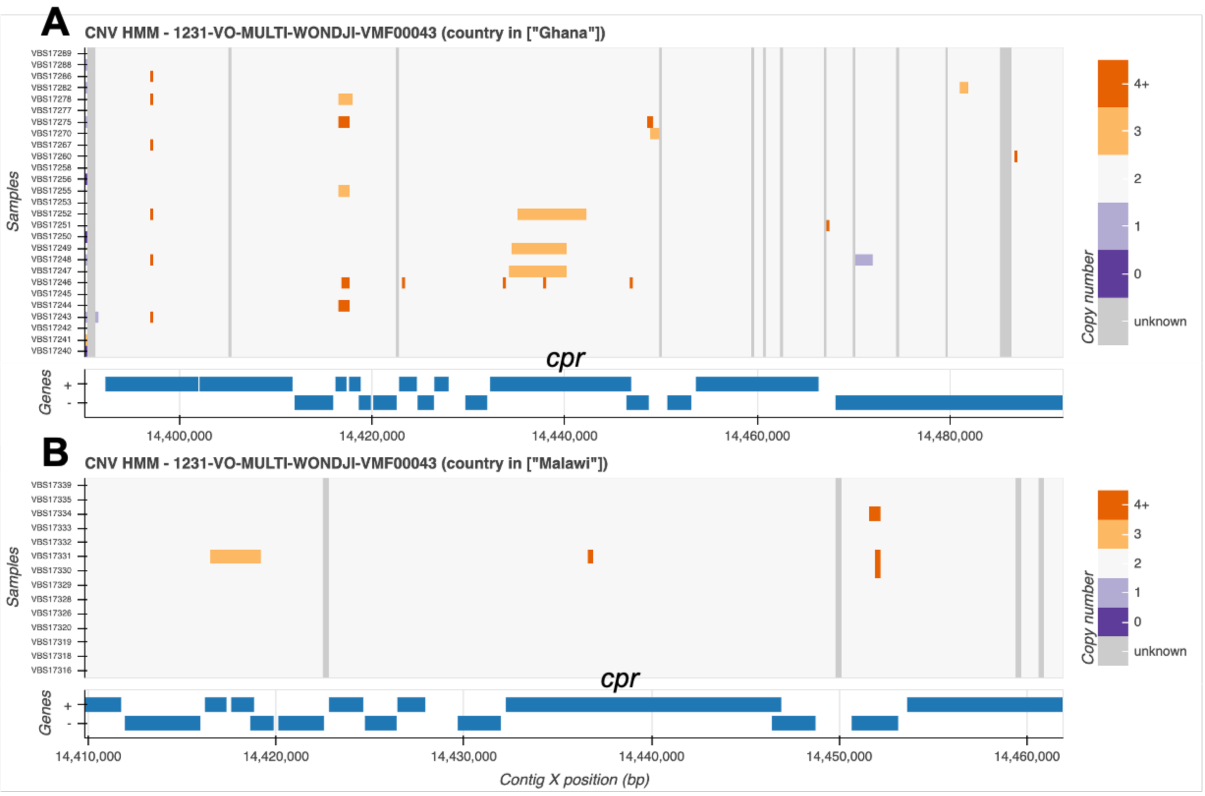

Supplementary figure 22: CNV HMM plot showing copy number state in *An. funestus* population from Ghana. The vertical color bar represents discrete copy number states used in CNV (Copy Number Variation) heatmaps. Each color corresponds to a specific copy number value for each sample: dark orange indicates high-level amplification (copy number ≥ 4), medium orange represents moderate amplification (copy number = 3), white denotes normal diploid copy number (copy number = 2), light purple reflects single-copy loss (copy number = 1), dark purple signifies homozygous deletion (copy number = 0), and gray indicates unknown or missing copy number data.

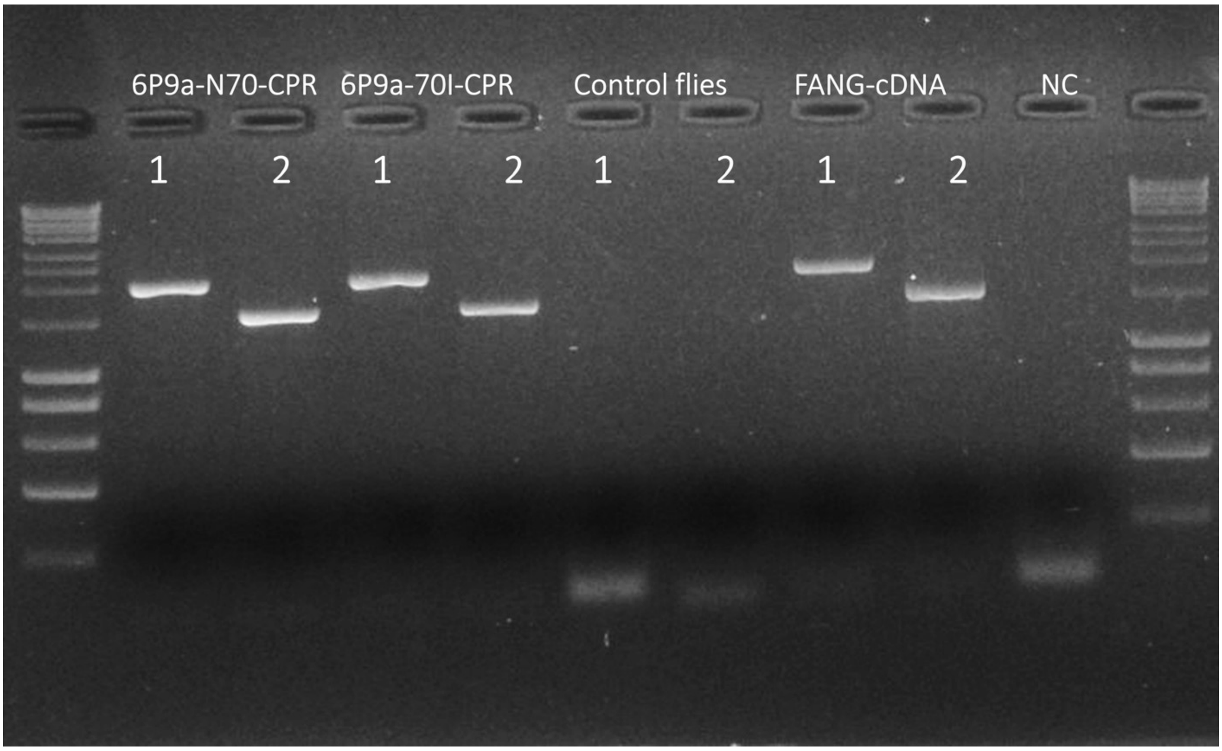

Supplementary figure 23: Confirmation of *CYP6P9a* and *CPR* genes expression in 6P9a-N70-CPR and 6P9a-70I-CPR transgenic Drosophila, and absence of expression in control flies, trough semiquantitative PCR.

Band 1 (~2000 bp) indicates the presence of the CPR gene; Band 2 (~1500 bp) indicates the presence of the CYP6P9a gene. No amplification was observed in the control Drosophila. "CN" represents the negative control (no template). cDNA from the Anopheles funestus susceptible laboratory strain (FANG) was used as a positive control.

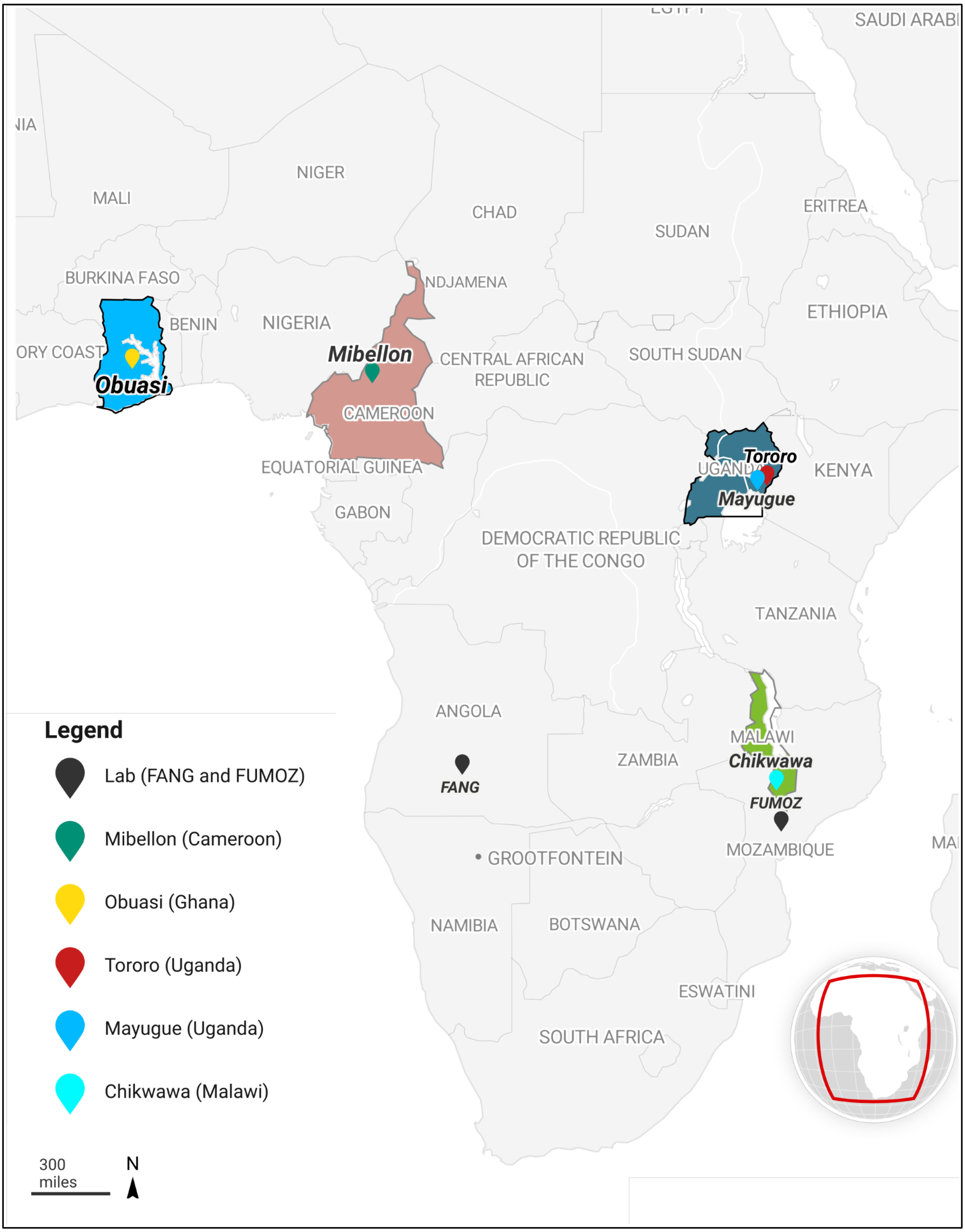

Supplementary figure 24: Study sites map.

Supplementary Tables

Supplementary table 1: Temporal overexpressed genes associated with pyrethroid resistance escalation in *An. funestus* across Africa. 20
Supplementary table 2: Genes overexpressed in dose response associated with pyrethroid resistance escalation in *An. funestus* in Southern Africa. 20
Supplementary table 3: Alignment metrics for Pool-seq data. 20
Supplementary table 4: Coverage statistics of PoolSeq data. .... **Error! Bookmark not defined.**
Supplementary table 5: Non-synonymous polymorphisms associated with selective sweeps in *An. funestus* across Africa. 21
Supplementary table 6: Duplications associated with selective sweeps in *An. funestus* across Africa. 21
Supplementary table 7: Polymorphism pattern at *CPR* gene in *An. funestus* across Africa. 21
Supplementary table 8: Genotype-Phenotype association of N70I variants in An. funestus in Malawi. 21
Supplementary table 9: Metadata of mosquito samples used for Pool-seq experiments. 23
Supplementary table 10: List of primers used for the functional validation 24

Supplementary table 1: Temporal overexpressed genes associated with pyrethroid resistance escalation in An. funestus across Africa.

This table presents a list of overexpressed genes in *An. funestus* across Africa taking into account various temporal comparisons (excel format).

Supplementary table 2: Genes overexpressed in dose response associated with pyrethroid resistance escalation in *An. funestus* in Southern Africa.

This table presents a list of overexpressed genes in *An. funestus* across Africa taking into account various comparisons (excel format)

Supplementary table 3: Alignment metrics for Pool-seq data.

Sample	Raw Reads (R1+R2+R0)	Aligned Reads, filtered (%)	Aligned in Pair (%)	Properly Paired (%)	Improper Paired (%)
**Cameroon_Unx_2021**	175255352	156650142 (89%)	155830786 (99%)	155830786 (99%)	9985670 (0.06%)
**Ghana_2021**	184445579	134084673 (73.61%)	132728190 (98.98%)	125078247 (94.23%)	7649943 (5.70%)
**Uganda_Tor_2009**	228232419	209075310 (93.36%)	208359842 (99.65%)	197210142 (94.64%)	11149700 (5.33%)

**Uganda_Myg_2021**	170588606	98461619 (58.14%)	96564426 (98.07%)	90127617 (93.33%)	6436809 (6.53%)
**Malawi_2021**	181468083	156017023 (87.28%)	153984850 (98.70%)	145087903 (94.22%)	8896947 (5.70%)

Supplementary table 4: Coverage statistics of PoolSeq data.

Sample	Coverage	meandepth	meanbaseq	meanmapq
**Cameroon_Unx_2021**	**98.91**	80.25	35.50	46.47
**Ghana_2021**	98.85	70.08	35.43	46.49
**Uganda_Tor_2009**	99.05	105.28	35.50	44.90
**Uganda_Myg_2021**	98.83	51.03	35.50	46.57
**Malawi_2021**	99.14	80.47	35.53	46.85

Supplementary table 5: Non-synonymous polymorphisms associated with selective sweeps in *An. funestus* across Africa.

This table presents a list of the key non-synonymous point mutations located within the major selective sweeps identified in *Anopheles funestus* across Africa (excel format).

Supplementary table 6: Duplications associated with selective sweeps in *An. funestus* across Africa.

This table presents the key duplications detected within the major divergent genomic loci in *Anopheles funestus* across Africa (excel format).

Supplementary table 7: Polymorphism pattern at *CPR* gene in *An. funestus* across Africa.

Sample	*N*	*S*	*H*	*Hd*	*Pi*	*Tajima's D*	*Fu and Li's D**
Cam_alive	10	31	10	1	0.004	-1.03 ns	-1.03 ns
Cam_dead	10	27	10	1	0.004	-0;54 ns	-0.36 ns
**Cam.Al_De**	**20**	**41**	**20**	**1**	**0.004**	**-1.10 ns**	**-1.37 ns**
Uga_alive	10	29	10	1	0.004	-0.64 ns	-0.22 ns
Uga_dead	10	28	9	0.98	0.004	-0.47 ns	-0.14 ns
**Uga_Al_De**	**20**	**40**	**19**	**0.99**	**0.004**	**-0.88 ns**	**-0.88 ns**
Mal_alive	18	13	9	0.80	0.001	-1.64 ns	-1.98 ns
Mal_dead	10	4	5	0.77	0.0008	0.56 ns	0.45 ns
**Mal_Al_De**	**28**	**14**	**11**	**0.77**	**0.001**	**-1.61 ns**	**-2.69 ns**
Fang	10	11	3	0.64	0.002	1.69 ns	0.79 ns
Fumoz	10	0	0	0	0	0	0
**Total**	**88**	**78**	**54**	**0.96**	**0.004**	**-1.61 ns**	**-3.51 ****

Supplementary table 8: Genotype-Phenotype association of N70I variants in *An. funestus* in Malawi.

Genotype	Alive	Dead	Combination	OR [95% CI]	P-value

**I/I**	2	3	I/I vs N/N	0.53 [0.03 - 8.30]	1
**N/I**	12	4	N/I vs N/N	2.17 [0.22 - 20.70]	0.63
**N/N**	4	3	I/I vs N/I	0.24 [0.01 - 2.91]	0.28

P > 0.28

Supplementary table 9: Metadata of mosquito samples used for Pool-seq experiments.

Country	Location	GPS coordinates	Year	N (Pooled genomes)	Month	Season	Climate	Interventions	Main activities
**Cameroon**	Mibellon	6°46′ N, 11°70′E	2021	40	July	Wet	Tropical/Equatorial	Bed nets	Agriculture
**Cameroon ***	Mibellon	6°46′ N, 11°70′E	2014-2015	40	February	Dry	Tropical/Equatorial	Bed nets	Agriculture
**Ghana***	Obuasi	5°56′N, 1°37′W	2014	40	March	Dry	Tropical/Equatorial	Bed nets	Agriculture
**Ghana**	Obuasi	5°56′N, 1°37′W	2021	40	July	Wet	Tropical/Equatorial	Bed nets	Agriculture
**Uganda***	Tororo	0°45’N, 34°5’E	2014	40	March	Wet	Tropical/Equatorial	Bed nets + IRS	Agriculture
**Uganda***	Tororo	0°45’N, 34°5’E	2009	40	March	Wet	Tropical/Equatorial	Bed nets + IRS	Agriculture
**Uganda**	Mayuge	0◦23′10.8” N, 33◦37′16.5” E	2021	40	October	Wet	Tropical/Equatorial	Bed nets	Agriculture
**Malawi**	Chikwawa	16°1’S, 34°47’E	2021	40	June	Dry	Tropical	Bet nets	Agriculture
**Malawi***	Chikwawa	16°1’S, 34°47’E	2014	40	January	Wet	Tropical	Bed nets	Agriculture

* represents samples already published in weedall et al., 2020 used for temporal analysis. N represents sample size used for PoolSeq experiments.

Supplementary table 10: List of primers used for the functional validation.

Genes	Primer's name	Sequences
CYP6P9A	6P9a/b BamHI Forward	CTCGAGTCACAATTTTTCCACCTTCAAGT
	6P9a XhoI Reverse	CTCGAGTTACACCTTTTCTACCTTCAAGT
CPR	CPR BglII Forward	AGATCTATGGACGCCCAGGCAGAAAT
	CPR Nhel Reverse	GCTAGCTTAGCTCCACACATCCGCCGA
PC5-KanP2A	PC5 Forward	AAGCAGCAGATTACGCGCAG
	PC5 Reverse	GCCATCACGAGATTTCGATTCC
	pC5Kan-P2A_internal F	CTTCAGCAGGGAGAAGTTGGTG
	pC5Kan-P2A_internal R	ACGTGGAGGAGAACCCCG
pUAS	pUAS Forward	ATTGCGATTCTATGCGGAAC
	pUAS Reverse	CCCATTCATCAGTTCCATAGGTTG
