# Supplementary material for: Genomic Drivers of Pyrethroid Resistance Escalation in the Malaria Vector Anopheles funestus Across Africa
Source: Mol Biol Evol. 2025 Oct 24;42(10):msaf251. doi: 10.1093/molbev/msaf251 (PMC12550562; doi:10.1093/molbev/msaf251)
Supplement: msaf251_Supplementary_Data [file msaf251_supplementary_data.zip › Supplementary_Text.pdf]

Supplementary results

Genes commonly and temporarily overexpressed in three countries

The genes commonly overexpressed in three countries showed low fold changes as compared to the genes commonly upregulated across the four countries. Also, a difference in the number of genes shared between countries varying considerably, with 12 genes that were shared between Cameroon, Malawi and Ghana, while 4 genes were shared between Cameroon, Malawi and Uganda, and only one gene was specific to Cameroon, Uganda and Ghana.

The 12 genes commonly overexpressed in Cameroon, Malawi and Ghana included two cytochrome P450s *CYP4J9* (FC: 2.1-6.5), *CYP6AD1* (FC: 2.0-2.8); cuticular protein *AFUN004576* (FC: 2.0-2.6), zinc finger protein *AFUN010686* (FC: 2.1-3.1) and CLIP domain serine protease *AFUN022134* (FC: 2.0-2.7). Further, two odorant-binding proteins, Obp2 (FC: 2.2-3.5) and *AFUN007246* (FC: 2.2-2.3), the gustatory receptor Gr53 (FC: 2.1-4.1), a carboxylic ester hydrolase *AFUN000775* (FC: 2.3-3.1), a transcription factor *AFUN006721* (FC: 2.2-2.7), an ornithine decarboxylase *AFUN010220* (FC: 2.2-2.9) and the vitellogenic carboxypeptidase-like protein *AFUN003727* (FC: 2.1-2.3).

In Cameroon, Malawi and Uganda four overexpressed genes were commonly shared out of which two cytochrome P450s *CYP6M2* (FC: 2.0-6.4) and *CYP6N1* (FC: 2.2-3.1), the glutathione S transferase microsomal *GSTMS1* (FC: 2.7-3.8) and the heat shock protein *AFUN019570* (FC: 4.2-11.1).

Between Cameroon, Uganda and Ghana only one key gene, the chemosensory protein CSP3 was commonly overexpressed with fold change ranging from 2.2-5.1. In fact, sensory appendance protein from the mosquito leg has been shown to drive pyrethroids resistance in *Anopheles gambiae* by sequestering the insecticide, thus reducing the concentration penetrating the insect body [6].

Genes commonly and temporarily overexpressed in two countries

Looking side by side for genes commonly overexpressed in two countries, we notice that Cameroon shared the greatest number of genes (48) with three other countries (Malawi: 27, Uganda: 13 and Ghana: 8), followed by Malawi which shared 12 genes with Uganda

(7) and Ghana (5). In addition, nine genes were found commonly overexpressed between Uganda et Ghana.

Among the 27 overexpressed genes between Cameroon and Malawi, three heat shock proteins were the top with fold changes ranging from 4.6-10.6, 4.1-7.1 and 3.2-3.9 for *AFUN019513*, *AFUN019289* and *AFUN019671*, respectively. Further, six cuticular proteins were evident: *AFUN008930* (FC: 3.0-5.2), *AFUN018956* (FC: 3.7-4.0), *AFUN019455* (FC: 2.8-3.9), *AFUN004759* (FC: 2.9-3.4), *AFUN021421* (FC: 2.3) and *AFUN021424* (FC: 2.3-2.6), two cytochrome P450s with roughly 2.2 FC for both *AFUN020179* and *CYP4G17*, two ABC transporters *AFUN016633* (FC: 2.4-2.6) and *AFUN002834* (FC: 2.1-2.4). Other overexpressed genes belong to several gene families including glutathione peroxidase, solute carrier family, gustatory receptor, odorant biding proteins, glycine N-methyltransferase, carboxylesterase, V-type proton ATPase and F type H+-transporting ATPase.

Many genes belonging to different enzyme families were commonly overexpressed temporally in Cameroon and Uganda. Overall, 13 genes were overexpressed with a predominant expression of the cytochrome P450 *CYP6P4b* (*AFUN019365*) with fold changes ranging from 4.6 to 12.9 in Uganda and Cameroon, respectively, followed by the cuticular protein *AFUN005218* with FC of 2.4 and 4.4 and the glucuronosyl transferase (*AFUN019845*) (FC: 3.2-3.5). The Aquaporin 1 (*AFUN000995*) and the salivary gland protein SG6 (*AFUN003883*) were also overexpressed to a small extend with fold changes of 2.2 to 2.5. The remaining genes showed an expression ranging from 2.0 to 3.7-fold changes including but not limited to the ionotropic receptor, serine protease and chitinase3-like protein.

Among the 9 genes commonly overexpressed in Cameroon and Ghana, the cytochrome P450 *CYP9K1* was by far the most overexpressed gene with a massive increased expression of 25.7 and 4.9-fold changes in Cameroon and Ghana, respectively. The second in rank was the Carboxylic ester hydrolase *AFUN016265* showing expression varying from 2.3 to 4.6-fold changes. Beside the genes, we noticed an overexpression but at a lower extent (FC: 2.0-3.9) of the P450 *AFUN019426*, the ABC transporter *AFUN020240*, the odorant receptor *AFUN015737*, the peroxidase *AFUN021932*, an ionotropic receptor *AFUN011601* and the solute carrier family *AFUN005963*.

Between Malawi and Uganda mosquito populations, seven genes were commonly overexpressed out of which two cytochrome P450; *CYP6AA1* (FC: 3.0) and *CYP6AA2* (FC: 2.0), the heat shock protein *AFUN021277* (FC: 2.5-2.8), the solute carrier family *AFUN000622* (FC: 2.2) and ABC transporter *AFUN015978* (FC: 2.0). Interestingly, we found the histone H2A *AFUN015753* overexpressed with fold changes varying from 2.1-2.3 in Uganda and Malawi, respectively.

Mosquitoes from Malawi and Ghana shared four overexpressed genes, including the membrane-bound transcription factor site-1 protease (*AFUN008650*) with fold changes of 2.2 and 2.0, respectively, and *AFUN011620* which is an orthologue of cuticular protein in *An. gambiae* (FC: 2.7–2.9). Additionally, a zinc finger protein, *AFUN009976* (FC: 2.7 and 2.2), and the thymus-specific serine protease *AFUN003520* (FC: 2.2–2.8) were also commonly overexpressed in both populations.

In Uganda and Ghana populations, a total number of 9 genes were commonly overexpressed. This list of genes was dominated by 4 genes belonging to the odorant binding protein family: *AFUN018886*, *AFUN006832*, *AFUN018679* and *AFUN008835* with fold changes ranging from 2.0 to 3.6, the ABC transporter *AFUN007483* (FC: 2.0 - 2.1) and the cytochrome P450 *AFUN019523* (FC: 2.6 - 3.2). Other gene families comprised the aldehyde dehydrogenase *AFUN008118*, the UDP-glucuronosyltransferase (*AFUN016158*) and the pancreatic lipase-related protein (*AFUN021120*), all with fold change varying from 2.1 to 2.6.

Genes temporarily over-expressed in each country

Attention was given to locally and temporally differentially expressed genes (DEGs) in each country to capture the transcriptomes associated with increased insecticide resistance in *Anopheles funestus* over time. Unlike the genes commonly overexpressed across different African settings that may be used for subregional interventions, the potential candidate genes identify here can be used to design targeted vector control interventions. However, compared to the commonly overexpressed genes, the expression changes in the unique genes were less pronounced, with fold changes

ranging from 2.0 to 5.7. Overall, 35, 22, 5 and 2 genes were uniquely increased in expression over time in Cameroon, Malawi, Uganda and Ghana, respectively.

The temporal transcriptome analysis in Cameroon revealed overexpression of several genes, including nine cytochrome P450s (*CYP307A1*, *CYP6Z1*, *CYP6Z3*, *CYP4J5*, *AFUN001382* and *AFUN001383*, *CYP9J5*, *CYP6P9A*, *CYP6P9b*, and *CYP4D22*), three ABC transporters (*AFUN009304*, *AFUN002180*, and *AFUN009878*), and one carboxylesterase (*AFUN016119*). Additionally, there was overexpression of a histone lysine N-methyltransferase (*AFUN000188*, FC = 2.11), a transcription factor BTF3 (*AFUN002724*, FC = 2.07), two alkaline phosphatases (*AFUN002611* and *AFUN019935*, FC = 5.7-3.8), two solute carrier families (*AFUN021791* and *AFUN016000*, FC = 2.4-3.1), two thioredoxin peroxidases (*TPX4* and *AFUN018693*, FC = 2.1-2.6), a heme peroxidase 2 (*HPX*2, FC = 2.34), two xanthine dehydrogenases (*AFUN021193* and *AFUN020670*, FC = 2.1-2.3), and a probable salivary secreted peptide (*AFUN016112*, FC = 2.37) (supplementary table S1).

The temporal transcriptome analysis in Malawi identified 22 overexpressed genes with fold changes ranging from 2.0 to 3.1. These genes included several metabolic enzymes such as cytochrome P450s (*AFUN021098*, *CYP6AH1*, *AFUN008357*, *CYP305A1*, *CYP4AR1*, *CYP6N2*), the ABC transporter *AFUN016185*, Glutathione S-transferase *GSTU1* (*AFUN009234*), carboxylesterase *AFUN016367*, and *UGT* (*AFUN011189*). Additionally, two serine/threonine-proteins, two glycogen proteins, two F-type H+transporting ATPases, two ATP synthase subunit beta, one cuticular protein, one lipase, one ubiquinol-cytochrome c reductase iron-sulfur subunit, and Glycogenin-1 were also temporally overexpressed (supplementary table S1).

In Uganda, the temporal transcriptome analysis highlighted four overexpressed genes with fold changes ranging from 2.0 to 2.47. These genes include a histone acetyltransferase type B subunit (*AFUN006190*), a serine/threonine-protein (*AFUN019189*), a carboxylesterase (*AFUN000373*), and a peptide-O-fucosyltransferase (*AFUN021578*) (supplementary table S1).

In the Ghana population, only two genes were temporally overexpressed. These genes are Vitellogenin-3 (*AFUN022070*) with a fold change of 2.02, and Zinc finger protein (*AFUN020257*) with a fold change of 2.5 (supplementary table S1).

Gene ontology and pathway enrichment analyses

Temporal change

we examined 166 transcripts, which were found to be commonly overexpressed in multiple countries or in at least one country. These transcripts underwent gene ontology enrichment analysis, revealing associations with terms across three key aspects: molecular function, biological process, and cellular component.

In the molecular function, the most overrepresented terms included heme binding (GO:0020037; adjusted p-value: 5.4x10^-36), representing compounds comprising an iron-complexed porphyrin ring, and monooxygenase activity (GO:0004497; adjusted pvalue: 9.6x10-36), which catalyses the incorporation of molecular oxygen into compounds while reducing the other atom of oxygen to water. Other significant terms included iron ion binding (GO:0005506; adjusted p-value: 5.0x10^-34), odorant binding (GO:0005549; adjusted p-value: 6.6x10^-10), and glutathione transferase activity (GO:0004363; adjusted p-value: 2.6x10^-5), known for catalysing reactions involving the release of glutathione compounds (supplementary figure S5).

In the biological process aspect, enrichment was observed for terms such as sensory perception of smell (GO:0007608; adjusted p-value: 6.0x10-10), response to stimulus (GO:0050896; adjusted p-value: 1.6x10-2), aerobic electron transport chain (GO:0019646; adjusted p-value: 3.8x10^-2), and mitochondrial ATP synthesis coupled electron transmembrane transport (GO:0042775) (Supplementary figure S5).

In the cellular component compartment, three significantly enriched terms were identified: membrane (GO:0016020), cytochrome complex (GO:0070069), and mitochondrial respirasome (GO:0005746). The mitochondrial respirasome is particularly associated with the mitochondrial inner membrane, consisting of protein complexes involved in the mitochondrial electron transport system (the respiratory chain) (supplementary figure S5A).

Dose response in Malawi

From the 197 genes submitted to gene ontology annotation, most of the terms were enriched in the molecular function and biological process (supplementary figure S5). Looking at the molecular function, the GO:0005549 odorant binding was the most significant enriched term (adjusted p-value: 2.87x10-45) with 45 genes. Further, the olfactory receptor activity (GO:0004984, adjusted p-value: 7.25x10-31), the transmembrane signalling receptor activity (GO:0004888, adjusted p-value: 8.42x10-12), and the heme binding (GO:0020037, adjusted p-value: 1.37x10-11) were enriched with 28, 28 and 22 genes respectively. However, the monooxygenase activity (GO:0004497, adjusted p-value: 2.23x10-10) comprised only 20 genes. Other enriched terms included the tetrapyrrole binding (GO:0046906), iron ion binding (GO:0005506), the molecular transducer activity (GO:0060089), the signalling receptor activity (GO:0038023), oxidoreductase activity, acting on paired donors, with incorporation or reduction of molecular oxygen (GO:0016705) and the oxidoreductase activity (GO:0016491) with 20 to 32 genes in each term.

In the Biological process, five main terms were substantially enriched including the sensory perception of chemical stimuli (GO:0007606, adj. p-value: 3.73x10-54), the sensory perception (GO:0007600, adj. p-value: 4.07x10-51), the nervous system process (GO:0050877, adj. p-value: 7.33x10-50), the system process (GO:0003008, adj. p-value: 1.28x10-49) and the sensory perception of smell (GO:0007608, adj. p-value: 5.60x1048) with 55 genes in each except the latest with 45 genes.

The G0:0016020 corresponding to membrane (adj. p-value: 1.40x10-6) was the unique significant enriched term in the cellular component including 67 genes (supplementary figure S5B).

Polymorphisms associated with resistant loci in *An. funestus* populations across Africa

GSTs cluster

Whitin the *GSTs* locus, we found multiple missense mutations at moderate to high frequencies, widespread all over the *GST* genes with same at increase allelic frequencies. The most relevant SNPs occurred on *GSTD11* (P178T) and *GSTT1* (A224T) approaching fixation and at increasing frequencies in Central, East and West Africa but not in Southern

Africa. Other SNPs are found at moderate to high frequencies across the continent but interestingly, we found the famous mutation on *GSTe2* (L119F) conferring resistant to pyrethroids and DDT in Cameroon and Benin at moderately increasing frequencies in Western population compared to elsewhere over time (supplementary table S5).

GABA and Ace1

Scanning the *GABA* receptor yields two major SNPs at increasing frequencies over time in Central (A296S) and Western Africa and not elsewhere *GABA* (A296S, T345S) (supplementary table S5). The A296S have been shown to confer resistance to dieldrin (*rdl*) in *An. funestus* populations whereas the T345S was detected for the first time under selection in Ghana population [7], co-occurring with the former. Looking at the *Ace1* gene, no impactful SNPs were found across populations but some of them were at lower frequencies in central Africa, *Ace1* (N214T, S88A).

VGSC

Searching for knock down resistance SNPs around the voltage-gated sodium channel (VGSC) indicate evidence of several SNPs at low frequencies spread across the continent (supplementary table S5). The most pertinent one present at increase frequency over time was found in Central Africa, VGSC (L2057I) and in Eastern at lower frequency but not elsewhere (supplementary table S5). More importantly, other SNPs were found at higher frequencies in Central and Eastern African but not elsewhere. These SNPs included G793C, I768L, Q959H, N91S, N317S, I360K and V2031G (supplementary table S5).

Polymorphism pattern of *CPR* gene using SureSelect data across Africa

The genetic diversity analysis of *An. funestus CPR* across Cameroon, Uganda, Malawi, and laboratory strains (FANG and FUMOZ) reveal moderate variation with haplotype diversity (Hd) generally high across most populations and phenotypes (Hd = 1 or close to 1), except for Malawi and FANG (supplementary table S7), where it is slightly lower (Hd

= 0.77–0.80 for Malawi and 0.64 for FANG). Nucleotide diversity (Pi) is consistently low across all groups (Pi ≈ 0.004), with Malawi populations showing the lowest diversity, especially in the Mal_dead sample (Pi = 0.0008). The number of segregating sites (S) is moderate for Cameroon and Uganda populations (27–41), but markedly reduced in the Malawi samples, particularly Mal_dead (S = 4). FUMOZ shows no diversity (S = 0, H = 0), which is expected for a lab-maintained colony. Neutrality tests, including Tajima's D and Fu and Li's D*, mostly show nonsignificant results, with negative values across many populations/phenotypes, suggesting potential population expansion or purifying selection, though not statistically significant. However, the combined dataset (all populations) shows a significant Fu and Li's D* value (-3.51**), indicating a potential deviation from neutrality, possibly due to selection or demographic events impacting the population as a whole. Phylogenetic analysis indicates that the Malawi populations are different from other populations as Malawi alive and dead which clustered on their own whereas other highly diverse populations clustered together (Fig. 8C).

Variant calling at the *CPR* gene using PoolSeq data across our samples revealed major replacement polymorphisms with low to moderate allele frequencies overall (supplementary table S5). The most significant SNP, located at position 70, involves the substitution of asparagine (N) with isoleucine (I). This SNP, located near the *CPR* gene active site, was observed to increase in frequency from 0% in 2002 to 51% in 2021 in the Malawian *An. funestus* population (supplementary table S5).

Supplementary references

1. Daniels, R. W., Rossano, A. J., Macleod, G. T. & Ganetzky, B. Expression of Multiple Transgenes from a Single Construct Using Viral 2A Peptides in Drosophila. *PLOS ONE* **9**, e100637 (2014).
2. Tang, W. *et al.* Faithful expression of multiple proteins via 2A-peptide self-processing: a versatile and reliable method for manipulating brain circuits. *J Neurosci* **29**, 8621–8629 (2009).
3. Riveron, J. M. *et al.* A single mutation in the GSTe2 gene allows tracking of metabolically based insecticide resistance in a major malaria vector. *Genome Biol* **15**, R27 (2014).
4. González, M. *et al.* Generation of stable Drosophila cell lines using multicistronic vectors. *Scientific Reports* **1**, 75 (2011).
5. Markstein, M., Pitsouli, C., Villalta, C., Celniker, S. E. & Perrimon, N. Exploiting position effects and the gypsy retrovirus insulator to engineer precisely expressed transgenes. *Nature Genetics* **40**, 476–483 (2008).
6. Ingham, V. A. *et al.* A sensory appendage protein protects malaria vectors from pyrethroids. *Nature* **577**, 376–380 (2020).
7. Grau-Bové, X. *et al.* Evolution of the Insecticide Target Rdl in African Anopheles Is Driven by Interspecific and Interkaryotypic Introgression. *Mol Biol Evol* **37**, 2900–2917 (2020).
